# Influence of Virtual Reality Illusions on Balance Performance and Immersive User Experience in Young Adults: A Within-Subject Experimental Study

**DOI:** 10.2196/70376

**Published:** 2025-06-27

**Authors:** R Achintha M Abayasiri, Antonio Padilha Lanari Bo, Taylor J M Dick, Nilufar Baghaei

**Affiliations:** 1 School of Electrical Engineering and Computer Science, Faculty of Engineering, Architecture and Information Technology, The University of Queensland, Brisbane, Australia; 2 Department of Mechanical, Electrical and Chemical Engineering, Faculty of Technology, Art and Design, OsloMet – Oslo Metropolitan University, Pilestredet 35, Oslo, 0166, Norway, 47 67236635; 3 School of Biomedical Sciences, Faculty of Health, Medicine and Behavioural Sciences, The University of Queensland, Brisbane, Australia

**Keywords:** VR Illusions, center of pressure, user experience, balance, virtual reality

## Abstract

**Background:**

Rehabilitation interventions to improve standing balance are often tedious and complex, limiting user engagement and increasing the burden of the clinicians delivering them. Virtual reality (VR) has been incorporated into such practices as a solution and VR illusions have emerged as a method for perturbing balance within interventions. However, the influence of VR illusions on balance performance, such as center of pressure (CoP), and user experience metrics remain under explored.

**Objective:**

This study aimed to evaluate the impact of the VR illusions on standing balance and immersive user experience in young adults.

**Methods:**

Young healthy adults (N=15, aged 18‐35 years) played a VR table tennis game while standing on a force plate and were provided with eight directional and magnitude-based VR illusions scaled according to participants’ heights. VR illusions were generated by offsetting the position of the playing hand in VR and were provided through 8 trials for each participant. Each VR illusion was delivered throughout final 50 seconds of each 70-second trial. Absolute CoP displacements, directional tendency of CoP displacement, and game performance were analyzed to evaluate the impact of the VR illusions. Responses to the User Experience Questionnaire, Slater-Usoh-Steed Presence Questionnaire, NASA Task Load Index, and Virtual Reality Sickness Questionnaire were analyzed to assess the immersive user experience.

**Results:**

Both the magnitude of VR illusion and changes in VR illusion direction led to significantly greater CoP displacements, with high illusion magnitudes, and anterior and posterior directional illusions associated with higher CoP displacements. Conversely, those illusion magnitudes and directions were associated with low game performance. The directional tendency of the CoP displacements varied across the illusion directions but showed a significant association with the illusion directions. Questionnaire responses showed that participants had moderate to high immersive user experience within the VR illusion paradigm.

**Conclusions:**

This study provides a novel approach for the future development of more effective VR-based balance rehabilitation interventions. The results provide inspiration for the development of future VR-based exergames that can perturbate CoP direction and magnitude. By adjusting the difficulty level through directional and magnitude changes in VR illusions, exergames could provide a personalized rehabilitation experience.

## Introduction

Standing balance deficits pose significant challenges to activities of daily living. Balance rehabilitation paradigms have been developed as an effective approach to address balance impairments. A fundamental aspect of standing balance rehabilitation is the use of perturbations, which challenge postural control and promote improvement in balance performance [1-4]. Center of pressure (CoP) displacement is widely recognized as a key measure of balance performance [5,6], with minimal displacement in response to perturbations indicating improved postural control [7,8]. However, these perturbations are often delivered physically, and thus, demand substantial therapist involvement as well as constant supervision. Yet, the repetitive and monotonous nature of such activities can lead to decreased patient engagement over time [9,10].

Virtual reality (VR)–based standing balance rehabilitation interventions have emerged as a promising alternative to reduce therapist workload while enhancing patient engagement. By creating interactive and immersive environments, VR systems offer a more engaging approach than traditional methods, potentially increasing patient motivation and adherence to rehabilitation programs [9]. In addition, VR systems allow for precise control over rehabilitation exercises, enabling therapists to tailor interventions to individual patient needs and remotely monitor progress [10,11]. In this context, incorporating VR illusions to balance rehabilitation interventions to create perturbation effects, has gained attention as a method for challenging postural control during rehabilitation [12,13]. These VR perturbations are typically generated by manipulating the visual field, such as rotating the virtual environment’s field of view, which can induce transient effects on balance [14-18]. Another common VR technique involves the manipulation of environmental optic flow, where adjustments in virtual motion patterns affect postural control [19-23]. While optic flow speed has been shown to induce temporary postural instability, Ketterer et al [19] cautioned that prolonged exposure to such VR illusions may have negative consequences for postural stability.

Despite these advancements, maintaining participant adherence to VR-based perturbation interventions remains a challenge. Enhancing the perceived realism of VR illusions is crucial to ensuring their effectiveness in inducing the desired postural adjustments in participants. Immersive user experience plays a critical role in enhancing realism and acceptability of VR illusions for balance rehabilitation interventions [10,24]. Key components of immersive user experience include immersion [25,26], comfort [27], sickness [28], and satisfaction [29,30], all of which contribute to the overall success of a rehabilitation program [24,31-33]. Furthermore, studies have shown that gamified interventions not only enhance patient adherence through increased motivation but also improve balance outcomes [34-40]. However, the existing VR illusion–based balance rehabilitation interventions have largely overlooked the impact on user experience, an essential factor for long-term adherence to rehabilitation programs [10,24]. Some studies have noted that certain populations, particularly young, healthy adults, often disregard the VR illusions, undermining the intended perturbation effects [41].

It is apparent that the perturbation effect and immersive user experience should go hand in hand for a successful VR illusion–based balance rehabilitation intervention. Thus, in this study, we aimed to advance the understanding of how VR illusions impact standing balance and immersive user experience. By leveraging the brain’s ability to adapt to discrepancies between sensory inputs and motor outputs, known as sensorimotor adaptation [34-37], we generated VR illusions by offsetting the virtual hand position in a commercially available VR table tennis game. The choice of this game was influenced by several factors, including its real-life relatability [38,39], the ability to be played in a standing posture [32], simple game-mechanics, and easily adjustable parameters. In addition, the game’s haptic and auditory feedback had the potential to enhance immersive user experience, further supporting our decision to use it [24,33]. Participants played the game standing on a force plate while being prompted to recalibrate their movements to account for the hand offsets. Additionally, we examined the impact of this intervention on immersive user experience through questionnaire responses. Finally, by conducting a multiscale analysis that incorporates both biomechanics and human-computer Interaction metrics, we sought to draw inferences for future exergame designs and VR-based balance rehabilitation interventions.

## Methods

### Participants

A total of 15 healthy young adults (8 male and 7 female participants; mean age 27.7, SD 3.8 years; mean height 1.7, SD 0.1 m; mean weight 73.62, SD 16.9 kg) were recruited through advertisements posted on university notice boards, social media, and by word of mouth. Once participants expressed their interest, the research team contacted them via email to provide detailed information about the study and confirm their participation. All the recruited participants were reported to be right-hand dominant.

### Experimental Setup

**Figure 1.**
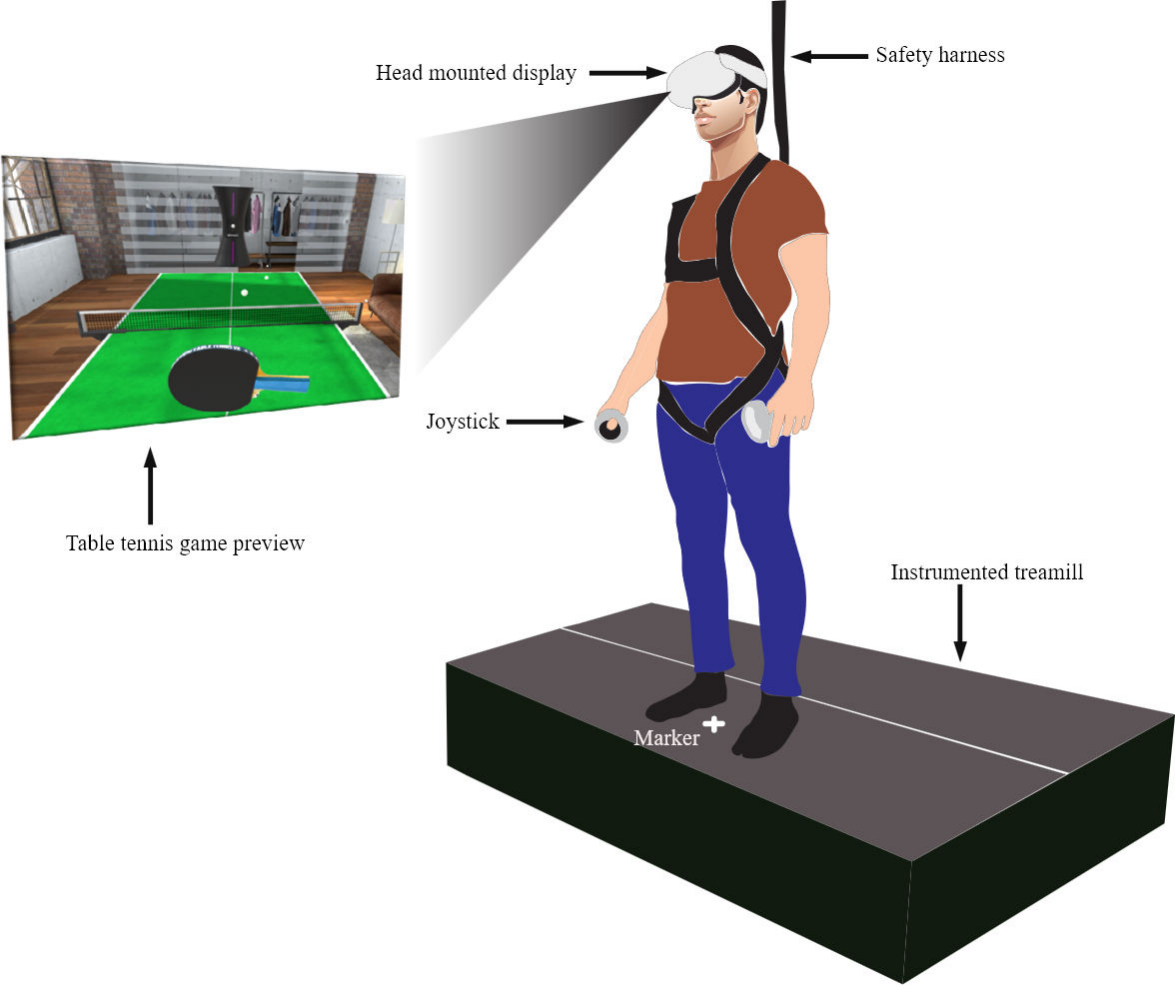
Experimental setup, in which participants stood on a static instrumented treadmill to play a virtual reality table tennis game. They were given instructions to keep their feet apart so that the marker on the treadmill would be in between their feet. They were asked to use the joystick in their dominant hand to hit the ball while keeping their feet steady on the treadmill.

The experimental setup was built inside the Neuromuscular Biomechanics laboratory of The University of Queensland (Figure 1). It consisted of immersive VR system based on a Head Mounted Display (HMD) (Quest 2; Meta, formerly Oculus), instrumented split belt treadmill (FIT5; Bertec), a safety harness, a personal computer, and a mobile phone camera (iPhone 14; Apple Inc) to record the participants’ gameplay on the monitor of the personal computer. Furthermore, the treadmill has force plates to measure ground reaction forces. During the experiment, treadmill was held static, and the force plate data were acquired at a frequency of 120 Hz and were processed in Qualisys Track Manager system (version 2022.2; Qualisys AB) to extract CoP data. CoP data were further filtered with second-order low-pass Butterworth filters at 6 Hz in MATLAB software (Release 2024a; The MathWorks Inc). Furthermore, a marker was placed on one of the belts of the treadmill to maintain the consistency of the feet placements of the participants.

### Experimental Protocol

**Figure 2.**
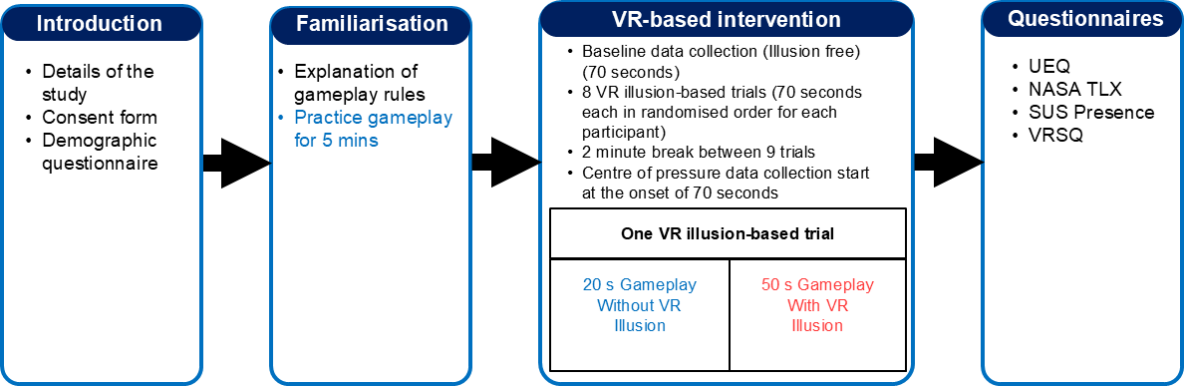
Overview of the experimental protocol. Experiment consists of 4 stages: introduction, familiarization, virtual-reality–based intervention, and post-gameplay data collection. NASA TLX: NASA task load index; SUS: Slater-Usoh-Steed; UEQ: User experience questionnaire; VRSQ: Virtual reality sickness questinnaire.

Experimental protocol consists of four stages: introduction, familiarization, data collection during VR-based intervention, and post data collection (Figure 2).

#### VR Game Configuration

For this study, the Ball Machine mode of the Eleven Table Tennis VR game (version 1.0; for Fun Labs) was used. The ball feeder mode was set to “default” to feed the ball through the centerline of the table at all times. The time scale was set to “1×” to feed 1 ball every 2 seconds. An independent application programming interface was used together with the game to enable offsetting the virtual representation of the participants’ hands with respect to the virtual body. Both the VR game and the external application programming interface were launched simultaneously on the SteamVR (version 2.6; Valve Corporation) platform.

#### Protocol

Participants were attached to the safety harness when they got onto the treadmill. They were instructed that they could not move their feet while playing the game. They kept their feet shoulder-width apart (approximately 40 cm), centered on the cross mark, and wore the HMD while staring straight ahead to align with the table in the game. Then, they were instructed to return the ball according to the general table tennis rules and to play without moving the feet from their starting position. In addition, participants were instructed that they could stop playing the game if they felt any discomfort, such as dizziness or nausea.

**Figure 3.**
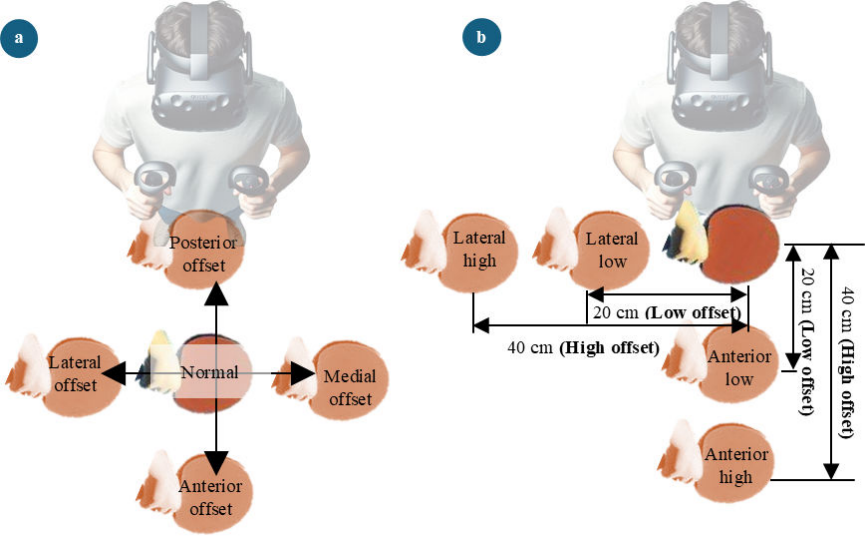
Types of virtual reality illusions. (**A**) Directions of the illusions and (**B**) construction of the Illusions combining both magnitudes and directions. Illusion magnitudes were scaled according to the participant’s height. (ie, if participant height is 180 cm, low and high values were equal to 20 cm and 40 cm, respectively). Here, examples are depicted for the construction of anterior-low, anterior-high, lateral-low and lateral-high type illusions.

All participants played the table tennis game for 5 minutes initially to become comfortable with the game environment and the rules of the gameplay (familiarization stage). Then, participants were asked to remove the HMD and rest for 2 minutes. After that, gameplay with VR illusions was started. In total, eight types of VR illusions were provided for the participants during this stage (Figure 3). These illusions were created by offsetting the virtual hand used to hit the ball in the VR table tennis game, in 4 directions, namely anterior, posterior, medial, and lateral. In addition, two levels of magnitude (low and high) were incorporated into the illusions. Furthermore, these magnitudes were scaled according to the height of the participants to ensure the uniformity of the applied illusions. Before the onset of this gameplay stage, participants were given further instructions about the gameplay for this stage. They were informed that they would encounter challenges to the usual gameplay but were still required to continue returning the ball to the other side of the table. Each participant played nine 70-second long trials of gameplay. Out of the nine trials, the first trial was conducted to collect the baseline data. Rest of the trials were based on the eight types of virtual illusions. Each virtual illusion–based trial used one type of virtual illusion and the order of applied virtual illusions for trials was randomized for each participant.

Before starting data collection for a trial, the experimenter waited until the participant completed 5 successful consecutive ball returns. Once this was observed, a timer and the collection of data from the force plate of the static treadmill, CoP were started for the trial. For the baseline trial, force data were recorded for 70 seconds. During the virtual illusion–based trials, the experimenter applied the virtual illusion throughout the last 50 seconds of the 70-seconds trial.

#### Post Gameplay Data Collection

After finishing the 9 VR gameplay trials including the baseline line trial, participants were asked to answer 4 questionnaires. Those questionnaires were Virtual Reality Sickness Questionnaire (VRSQ) [40], User Experience Questionnaire (UEQ) [42], NASA Task Load Index (NASA-TLX) [43], and Slater-Usoh-Steed (SUS) presence questionnaire [44].

### Statistical Analysis

All statistical analyses were performed using R software (version 4.4.1; R Foundation for Statistical Computing). Paired *t* tests were used to evaluate the significance of differences in absolute CoP displacement measures before and after illusion stages for different illusion types. In addition, paired *t* tests were used to assess the statistical significance of mean differences in absolute CoP displacement measures and the number of successful ball returns (gameplay score) across different illusion magnitude levels.

To examine variations in absolute CoP measures and gameplay scores based on the factors of illusion magnitude and direction, linear mixed-effect (LMEs) models were specified, including participants as a random factor, and interaction terms. Tukey post hoc tests were conducted to further analyze the differences between the levels of main effects. In addition, paired *t* tests were conducted to compare absolute CoP displacements between successful and unsuccessful ball returns. Significance was determined at the *P*<.05 level for all the aforementioned statistical tests. We analyzed absolute CoP displacement to capture CoP excursions [45], as considering directional CoP values would only reflect the tendency of movement direction. Furthermore, both mediolateral and anteroposterior directional absolute CoP displacements were considered in the analyses.

To investigate the association between directional tendency and illusion directions, the Fisher exact test, with simulated *P* values based on 10^6^ replicates generated through the Monte Carlo simulation was used. The Chi-square test was applied to assess the statistical significance of the relationship between illusion direction and gameplay status (successful or unsuccessful). In both cases, associations were considered significant at *P*<.05.

### Ethical Considerations

This study was conducted in the Neuromuscular Biomechanics Laboratory at The University of Queensland, Australia. All study procedures were approved by The University of Queensland Human Research Ethics Committee (approval number 2023/HE001569). Written informed consent was obtained from all participants before their involvement in the study. Participants had no history of leg injuries or fractures within the past 6 months and no neurological disorders affecting visual, cognitive, or vestibular function. Participation was voluntary and no compensation was provided. All recorded data were deidentified using a coding system to ensure confidentiality.

## Results

### Mean Absolute Center of Pressure Displacement

**Figure 4.**
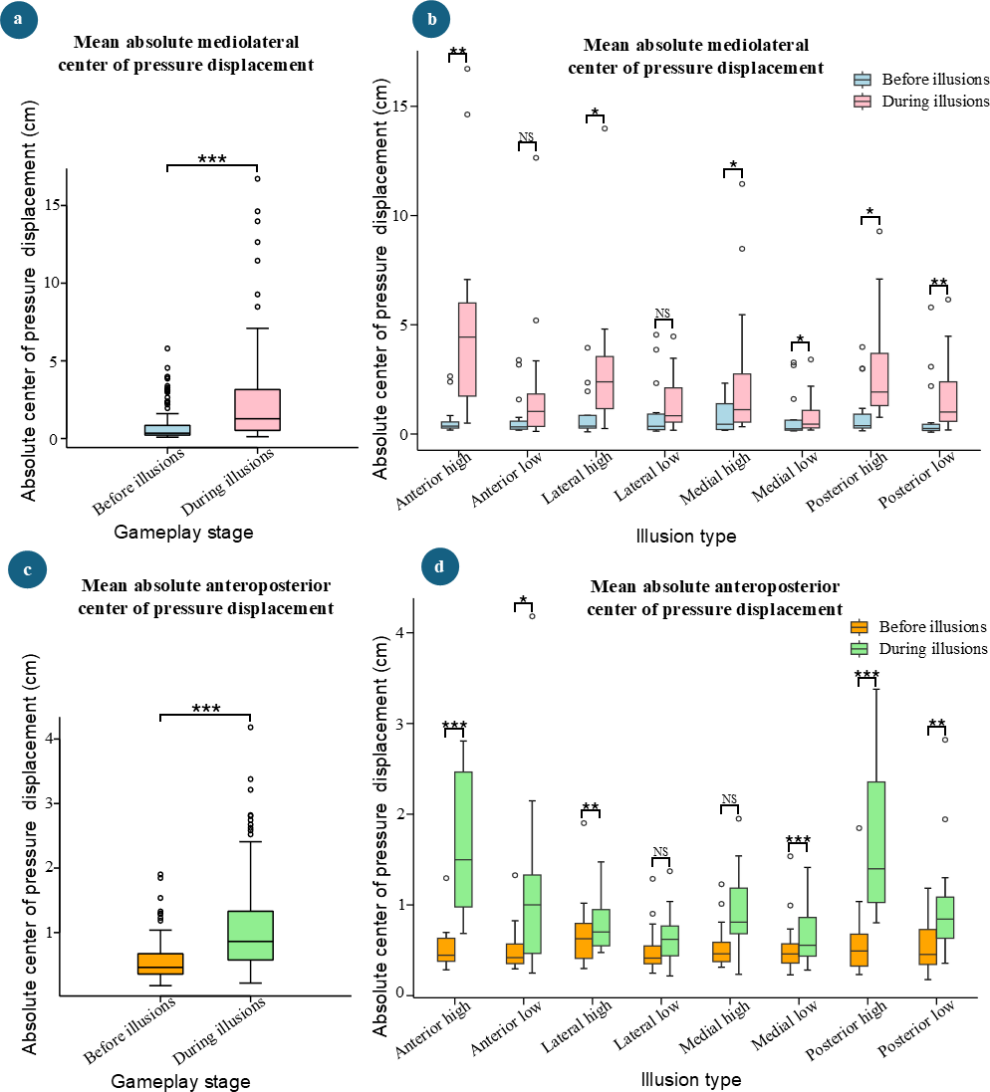
Comparison of absolute mean center of pressure displacement before and during illusions. (**A**) Absolute mean mediolateral center of pressure displacement comparison in gameplay stages. (**B**) Absolute mean mediolateral center of pressure displacement comparison across different types of virtual illusions. (**C**) Absolute mean anteroposterior center of pressure displacement comparison in gameplay stages. (**D**) Absolute mean anteroposterior center of pressure displacement comparison across different types of virtual illusions ( NS: *P* > 0.05, *P< 0.05, **P < 0.01, ***P < 0.001).

Figure 4 illustrates the comparisons of mean absolute CoP displacement in both the mediolateral and anteroposterior directions. There were significant increases in mean absolute CoP displacements during illusions compared with before the illusion stages for both mediolateral (*P*<.001, mean difference 1.62 cm) and anteroposterior (*P*<.001, mean difference 0.5 cm; Figure 4A and C) directions implying that the VR illusions were able to affect the mediolateral and anteroposterior stability of the participants.

Further comparisons of mean absolute mediolateral CoP displacement showed significant increases during illusions compared with before the illusions for several types of illusions: anterior high (*P*=.002, mean difference 4.4 cm), lateral high (*P*=.048, mean difference 1.8 cm), medial high (*P*=.025, mean difference 2.1 cm), medial low (*P*=.045, mean difference 0.4 cm), posterior high (*P*=.015, mean difference 2 cm), and posterior low (*P*=.03, mean difference 0.8 cm; Figure 4B). In contrast, no significant differences were observed for mean absolute mediolateral CoP displacement for anterior low (*P*=.093, mean difference 1.25 cm) and lateral low (*P*=.450, mean difference 0.1 cm) illusion types implying that the participants’ balance was not significantly impacted by these illusion types.

For mean absolute anteroposterior CoP displacement, significant increases were observed during the illusion stages compared with before the illusion stages for the following types: anterior high (*P*<.001, mean difference 1.1 cm), anterior low (*P*=.012, mean difference 0.6 cm), lateral high (*P*<.001, mean difference 0.3 cm), medial low (*P*<.001, mean difference 0.4 cm), posterior high (*P*<.001, mean difference 1.2 cm), and posterior low (*P*=.007, mean difference 0.5 cm; Figure 4D). However, no significant differences were observed for lateral low (*P*=.073, mean difference 0.1 cm) and medial high (*P*=.174, mean difference 0.1 cm) illusion types.

### Maximum Absolute Center of Pressure Displacement

**Figure 5.**
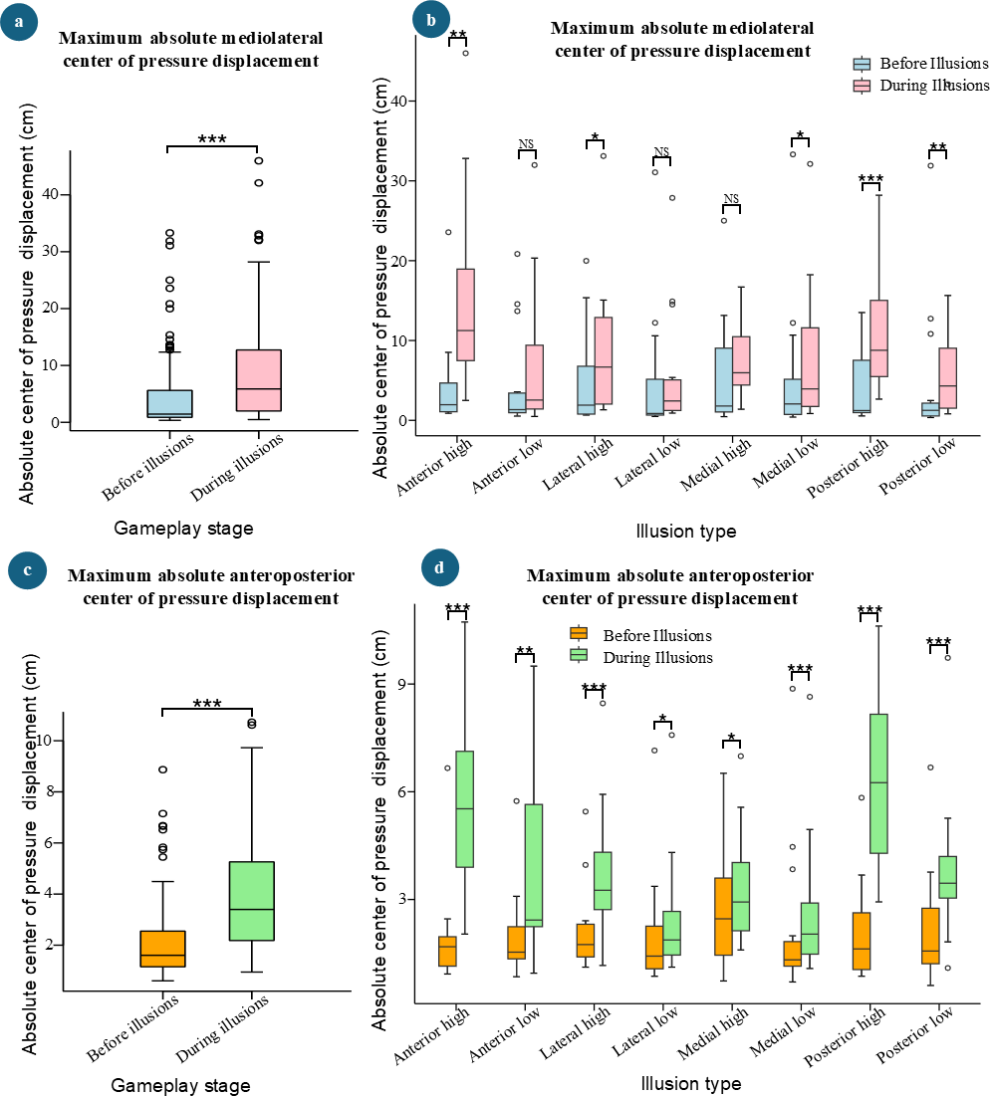
Comparison of absolute mean center of pressure displacement before and during illusions. (**A**) Absolute mean mediolateral center of pressure displacement comparison in gameplay stages. (**B**) Absolute mean mediolateral center of pressure displacement comparison across different types of virtual illusions. (**C**) Absolute mean anteroposterior center of pressure displacement comparison in gameplay stages. (**D**) Absolute mean anteroposterior center of pressure displacement comparison across different types of virtual illusions ( NS: *P* > 0.05, *P < 0.05, **P < 0.01, ***P < 0.001).

Figure 5 shows the comparisons of maximum absolute CoP displacement. Similar to the behavior of mean absolute CoP displacement, there were significant increases in maximum absolute CoP displacement during illusions compared with before the illusion stages in both the mediolateral (*P*<.001, mean difference 4 cm) and anteroposterior (*P*<.001, mean difference 1.9 cm) directions (Figure 5A and C).

Further analysis of maximum absolute mediolateral CoP displacement before and during the illusion stages for different illusion types revealed significant increases for the following illusion types: anterior high (*P*=.001, mean difference 10.7 cm); lateral high (*P*=.041, mean difference 3.8 cm); medial low (*P*=.048, mean difference 2.5 cm); posterior high (*P*<.001, mean difference 6.1 cm); and posterior low (*P*=.003, mean difference 3.5 cm; Figure 5B). Similar to the mean absolute mediolateral CoP comparisons, there were no significant differences in maximum absolute mediolateral CoP displacement for anterior low (*P*=.078, mean difference 2.7 cm) and lateral low (*P*=.496, mean difference 0.8 cm) illusion types. In addition, no statistically significant increase was observed for the medial high (*P*=.074, mean difference 2.3 cm) illusion type.

In contrast to the behavior of maximum absolute mediolateral CoP displacement, significant increases were observed in maximum absolute anteroposterior CoP displacement during illusion stages compared with before the illusion stages for all types of illusions: anterior high (*P*<.001, mean difference 3.8 cm); anterior low (*P*=.004, mean difference 2 cm); lateral high (*P*<.001, mean difference=1.6 cm); lateral low (*P*=.024, mean difference 0.5 cm); medial high (*P*=.014, mean difference 0.7 cm); medial low (*P*<.001, mean difference 0.6 cm); posterior high (*P*<.001, mean difference 4.2 cm), and posterior low (*P*<.001, mean difference 1.6 cm; Figure 5D).

### Linear Mixed Effects Models for Absolute Center of Pressure Displacements

**Figure 6.**
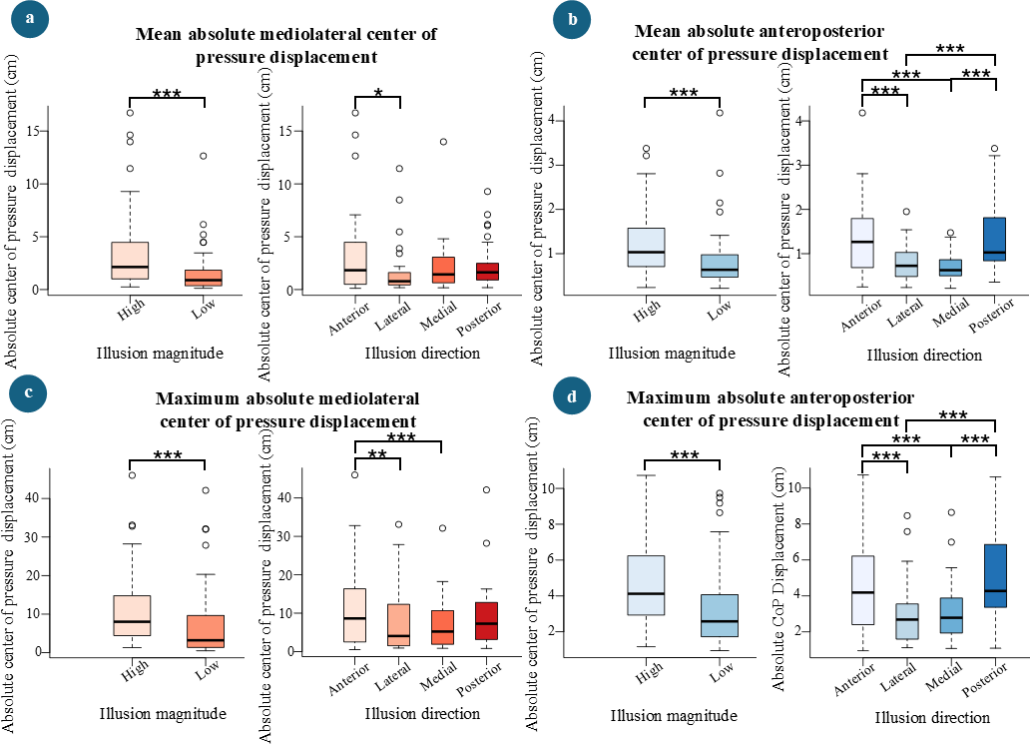
Absolute center of pressure displacement across identified main effects from the linear mixed-effects models. (**A**) Mean absolute mediolateral center of pressure displacement across different levels of main effects: illusion magnitude and illusion direction. (**B**) Mean absolute anteroposterior center of pressure displacement across different levels of main effects: illusion magnitude and illusion direction. (**C**) Maximum absolute mediolateral center of pressure displacement across different levels of main effects: illusion magnitude and illusion direction. (**D**) Maximum absolute anteroposterior center of pressure displacement across different levels of main effects: illusion magnitude and illusion direction. (*P < 0.05, **P < 0.01, ***P < 0.001)

ANOVA analyses on the LMEs found no significant interaction effect of illusion direction and magnitude for either mean absolute mediolateral CoP displacement (*F_3,98_*=0.81, *P*=.492) or mean absolute anteroposterior CoP displacement (*F_3,98_*=1.79, *P*=.155). However, illusion magnitude was identified as a significant main effect, contributing to increased mean absolute mediolateral (*F_1,98_*=18.24, *P*<.001) and anteroposterior (*F_1,98_*=19.30, *P*<.001) CoP displacements. In addition, illusion direction was found to significantly increase mean absolute CoP displacement in both the mediolateral (*F_3,98_*=3.12, *P*=.029) and anteroposterior (*F_3,98_*=14.04, *P*<.001) directions. Similarly, illusion magnitude was a significant main effect in increasing maximum absolute mediolateral(*F_1,98_*=12.25, *P*<.001) and anteroposterior (*F_1,98_*=28.08, *P*<.001) CoP displacements. Additionally, illusion direction significantly increased maximum absolute CoP displacement in both mediolateral (*F_3,98_*=3.13, *P*=.029) and anteroposterior (*F_3,98_*=14.51, *P*<.001) directions. A significant interaction effect was observed for maximum absolute mediolateral CoP displacement (*F_3,98_*=2.77, *P*=.046), but no such effect was found for maximum absolute anteroposterior CoP displacement (*F_3,98_*=1.77, *P*=.158). Comparison between the high and low illusion magnitude levels revealed that there were significant increases in both mean absolute mediolateral CoP displacement (*P*<.001, mean difference 1.9 cm) and mean absolute anteroposterior CoP displacement (*P*<.001, mean difference 0.4 cm) compared with the low magnitude level. High illusion magnitude levels also resulted in significant increases in maximum absolute mediolateral (*P*<.001, mean difference 3.4 cm) and maximum absolute anteroposterior (*P*<.001, mean difference 1.5 cm) CoP displacements compared with the low illusion magnitude levels (Figure 6A and B).

Pairwise comparisons using Tukey post hoc tests for illusion directions revealed a significant difference for the anteriorlateral pair (*P*=.015, estimate 1.8 cm) in mean mediolateral CoP displacement, while no other pairwise comparisons for mean mediolateral CoP displacement showed significant differences. For mean absolute anteroposterior CoP displacement, significant differences were observed for the following illusion direction pairs: anterior-lateral (*P*<.001, estimate 0.6 cm), anterior-medial (*P*<.001, estimate 0.7 cm), posterior-lateral (*P*<.001, estimate 0.6 cm), and posterior-medial (*P*<.001, estimate 0.7 cm). For maximum mediolateral CoP displacement, significant differences were found for the anterior-lateral (*P*=.005, estimate 6.2 cm) and anterior-medial (*P*<.001, estimate 7 cm) pairs, for the maximum absolute anteroposterior CoP displacement, the results were consistent with those for mean absolute anteroposterior displacement, revealing significant differences for the anterior-lateral (*P*<.001, estimate 1.8 cm), anterior-medial (*P*<.001, estimate 1.8 cm), posterior-lateral (*P*<.001, estimate 1.9 cm), and posterior-medial (*P*<.001, estimate 2 cm) pairs.

### Directional Tendency of Center of Pressure Displacement

**Figure 7.**
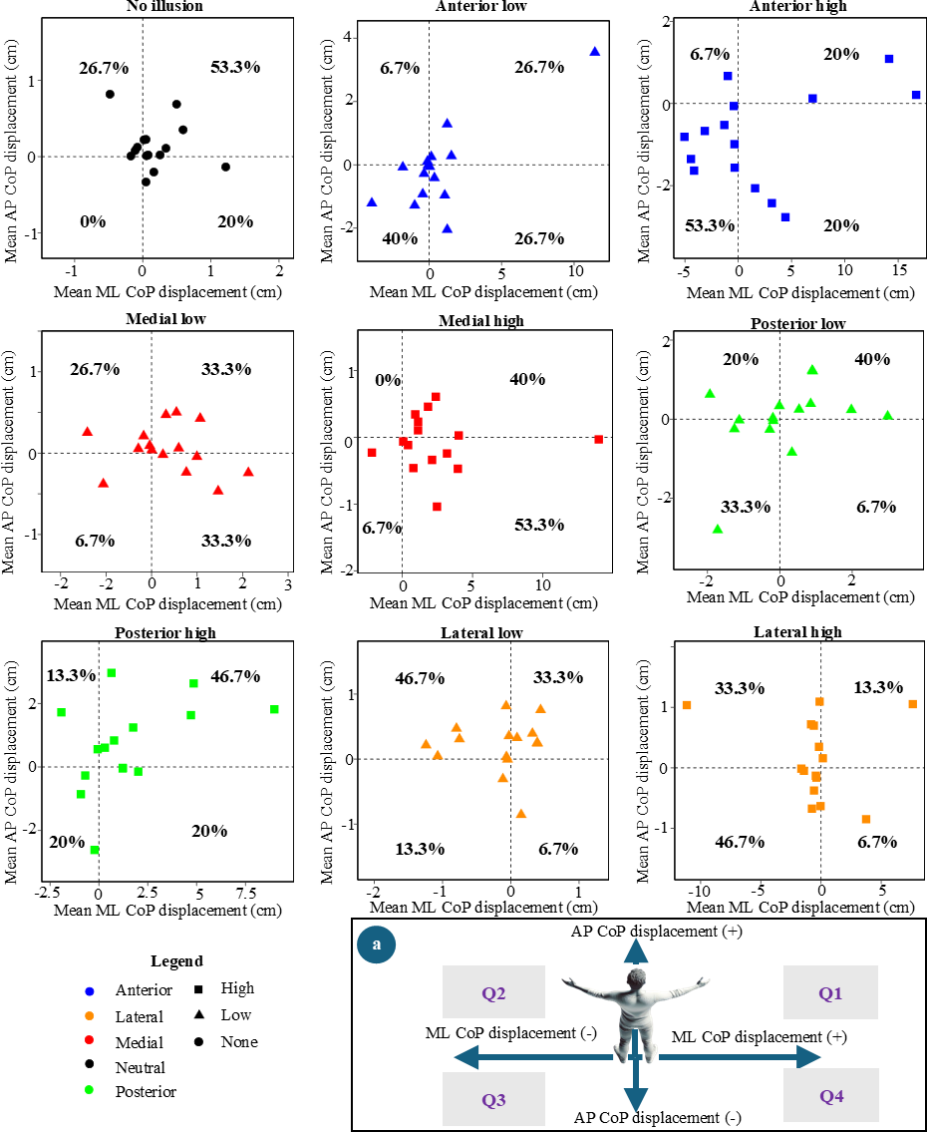
Directional tendency of mean center of pressure displacement across all illusion types (including no illusions) and the percentage distribution of center of pressure within each quadrant. (**A**) Definition of the quadrants. Q1-4: quadrants 1-4. AP: Anteroposterior; CoP: Center of pressure; ML: Mediolateral.

Figure 7 illustrates the directional tendency of CoP displacement, where the the mean values of CoP displacement were considered to reveal the direction of the CoP movement. Depending on the direction of the CoP displacement, quadrants were defined (Figure 7A).

For the no illusion stage (53.3%), posterior low illusion (40%), and posterior high illusion (46.7%), the majority of participants’ mean CoP displacements were observed in quadrant 1. Notably, the lateral low illusion (46.7%) showed the highest percentage of directional tendency in quadrant 2. For the anterior low illusion (40%), anterior high illusion (53.3%), and lateral high illusion (46.7%), the highest percentage of directional tendency occurred in quadrant 3. The medial high illusion (53.3%) revealed quadrant 4 as having the highest percentage for directional tendency. During the medial low illusion stage, participants exhibited a similar directional tendency in both quadrant 1 and quadrant 3, with 33.3% of the participants’ mean CoP displacements falling in each quadrant.

The directional tendency of the mean CoP displacement was further categorized by illusion directions (Table 1). The analysis revealed that quadrant 1 exhibited a higher directional tendency when the illusion direction was posterior (43.3%) and when there was no illusion (53.3%), compared with other illusion directions. Quadrant 2 showed the highest directional tendency during lateral directional illusions (40%), while quadrant 3 had the highest tendency for anterior directional illusions (46.7%). Finally, quadrant 4 ranked highest in directional tendency during medial directional illusions (43.3%). It was found that there is a significant association (*P*<.001) between illusion direction and the directional tendency of the mean CoP displacement (quadrant).

**Table 1.** Percentage of data points for mean center of pressure displacement by illusion direction and quadrant (quadrants were defined in Figure 7A).

Illusion direction	Quadrant 1 (%)	Quadrant 2 (%)	Quadrant 3 (%)	Quadrant 4 (%)
No illusion	53.3	26.7	0	20
Anterior	23.3	6.7	46.7	23.3
Posterior	43.3	16.7	26.7	13.3
Medial	36.6	13.3	6.7	43.3
Lateral	23.3	40	30	6.7

### Gameplay Performance versus Center of Pressure Displacement

**Figure 8.**
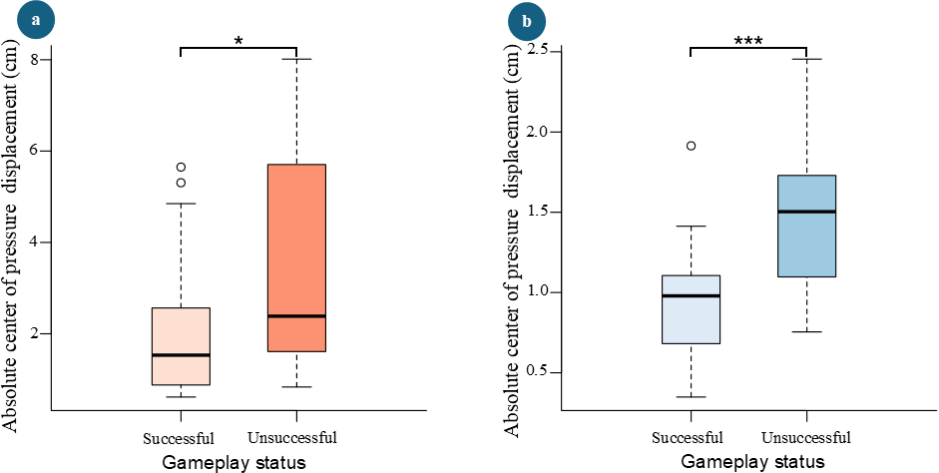
Mean absolute center of pressure displacement comparison across gameplay statuses. (**A**) In the mediolateral direction. (**B**) In the anteroposterior direction. (*P < 0.05, **P < 0.01, ***P < 0.001)

Figure 8 illustrates how mean absolute CoP displacement varied based on whether participants successfully returned the ball to the other side of the table or not.

Mean absolute CoP displacements in both the mediolateral (2.3, SD 1.8) cm and anteroposterior directions (0.9, SD 0.4) cm were lower when participants were successful, compared with unsuccessful attempts, where mean absolute CoP displacements in the mediolateral direction measured 3.5 (SD 2.6) cm and in the anteroposterior direction 1.4 (SD 0.5) cm. Furthermore, paired *t* test results indicated that the difference in absolute CoP displacements between successful and unsuccessful attempts was statistically significant in both the mediolateral (*P*=.031) and anteroposterior (*P*<.001) directions.

**Figure 9.**
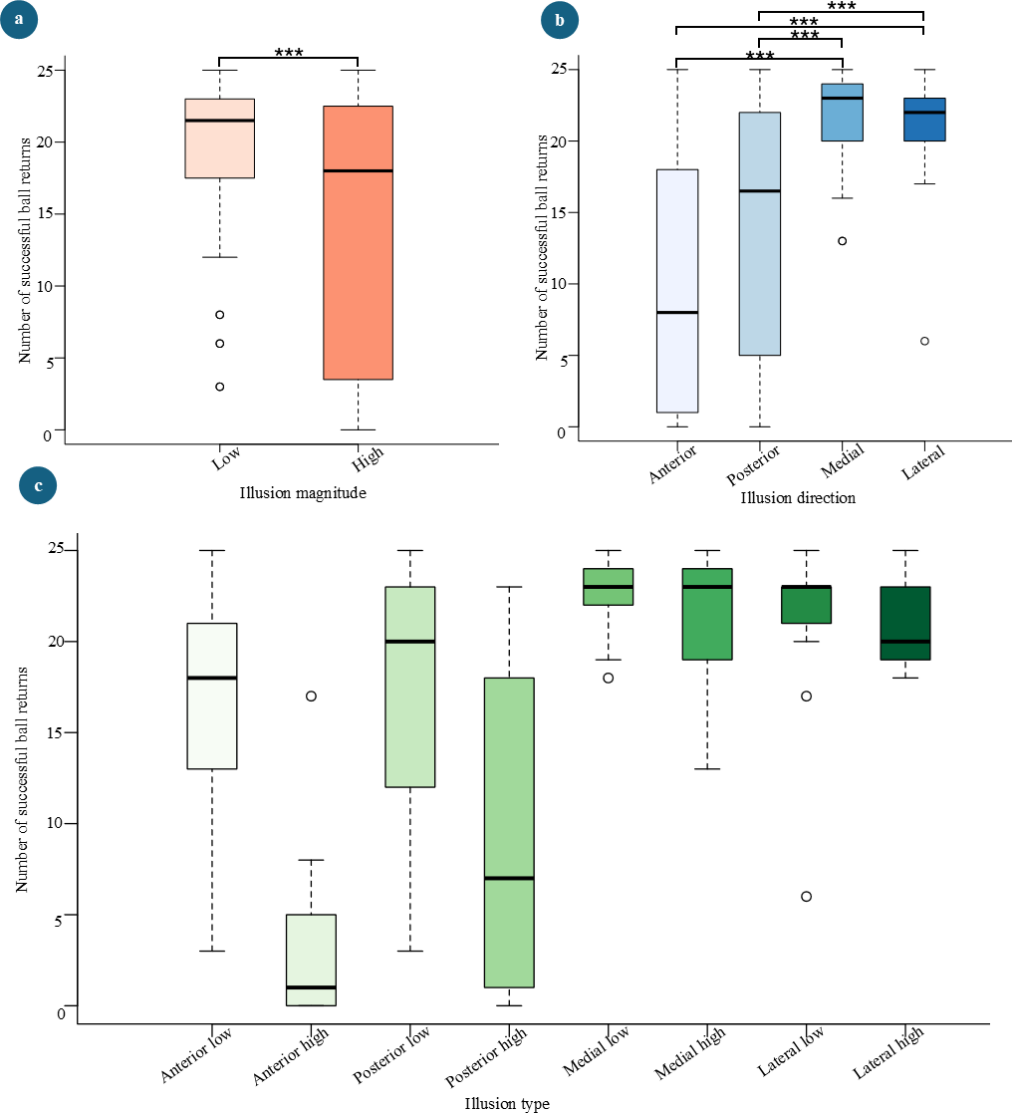
Number of successful returns of the balls. (**A**) Across illusion magnitude levels. (**B**) Across illusion direction levels. (**C**) Across illusion types. (*P < 0.05, **P < 0.01, ***P < 0.001)

ANOVA analysis conducted on the LME built for the gameplay score revealed that illusion magnitude (*F_1,84_*=28.64, *P*<.001) and illusion direction (*F_3,84_*=31.99, *P*<.001) were significant main effects in decreasing the score. In addition, a significant interaction effect between illusion magnitude and direction (*F_3,84_*=8.07, *P*<.001) was found, further contributing to a decrease in score. A significant difference between the scores during high and low illusion magnitude levels (*P*<.001) was reported, with an average decrease of 6 successful returns for the high magnitude condition (Figure 9A). Furthermore, significant differences were found in the following pairs of illusion directions: lateral-anterior (*P*<.001, estimate 11 hits), medial-anterior (*P*<.001, estimate 12 hits), lateral-posterior (*P*<.001, estimate 7 hits), and medial-posterior (*P*<.001, estimate 7 hits; Figure 9B).

A significant association between illusion direction and gameplay success (*P*<.001). Gameplay success was further categorized across the different illusion directions (Table 2) to quantify participants’ performance. Participants’ success percentages were lower during both anterior direction illusions (38.3%) and posterior direction illusions (55.4%) compared with the other 2 directions.

**Table 2.** Percentage of data points for gameplay status by illusion direction.

Illusion direction	Successful (%)	Unsuccessful (%)
Anterior	38.3	61.7
Posterior	55.4	44.6
Medial	86.3	13.7
Lateral	83.8	16.2

### Questionnaire Responses

#### User Experience Questionnaire

**Figure 10.**
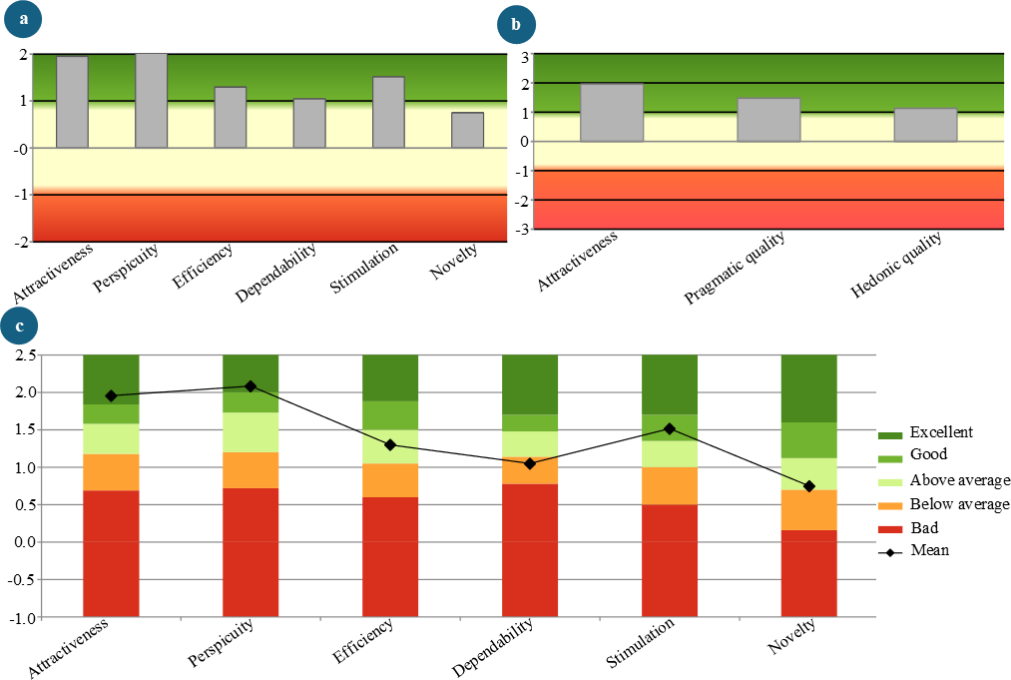
Distribution of user experience questionnaire responses. (**A**) Across user experience questionnaire scales. (**B**) Across attractiveness, pragmatic quality, and hedonic quality. (**C**) User experience questionnaire results in comparison with benchmark data.

The UEQ Data Analysis Tool (version 12) was used to analyze the UEQ responses [46]. The results indicate generally positive user perceptions (Figure 10). Attractiveness scored 1.96 (SD 0.42), suggesting that participants found the experiment appealing, while perspicuity, which reflects ease of use and learning, received the highest score of 2.08 (SD 0.46). Efficiency, measuring task performance ease, scored 1.30 (SD 0.72), and dependability scored 1.05 (SD 0.69). Stimulation, which reflects how engaging participants found the experiment, received a score of 1.52 (SD 0.54), while novelty, indicating perceived innovation, had the lowest score at 0.75 (SD 0.89; Figure 10A). Overall, pragmatic quality, which assesses task support, scored 1.48, and hedonic quality, reflecting enjoyment, was 1.13 (Figure 10B).

The UEQ benchmark results show strong performance in several areas (Figure 10C). Attractiveness and perspicuity received “Excellent” ratings, placing them within the top 10% of results. Efficiency was rated “Above Average,” with 25% of results performing better and 50% performing worse. Dependability was rated “Below Average,” with 50% of results performing better and 25% performing worse. Stimulation achieved a “Good” rating, with 10% of results outperforming it and 75% performing worse. Novelty was rated “Above Average,” with 25% of results better and 50% worse.

#### Slater-Usoh-Steed Presence Questionnaire

**Figure 11.**
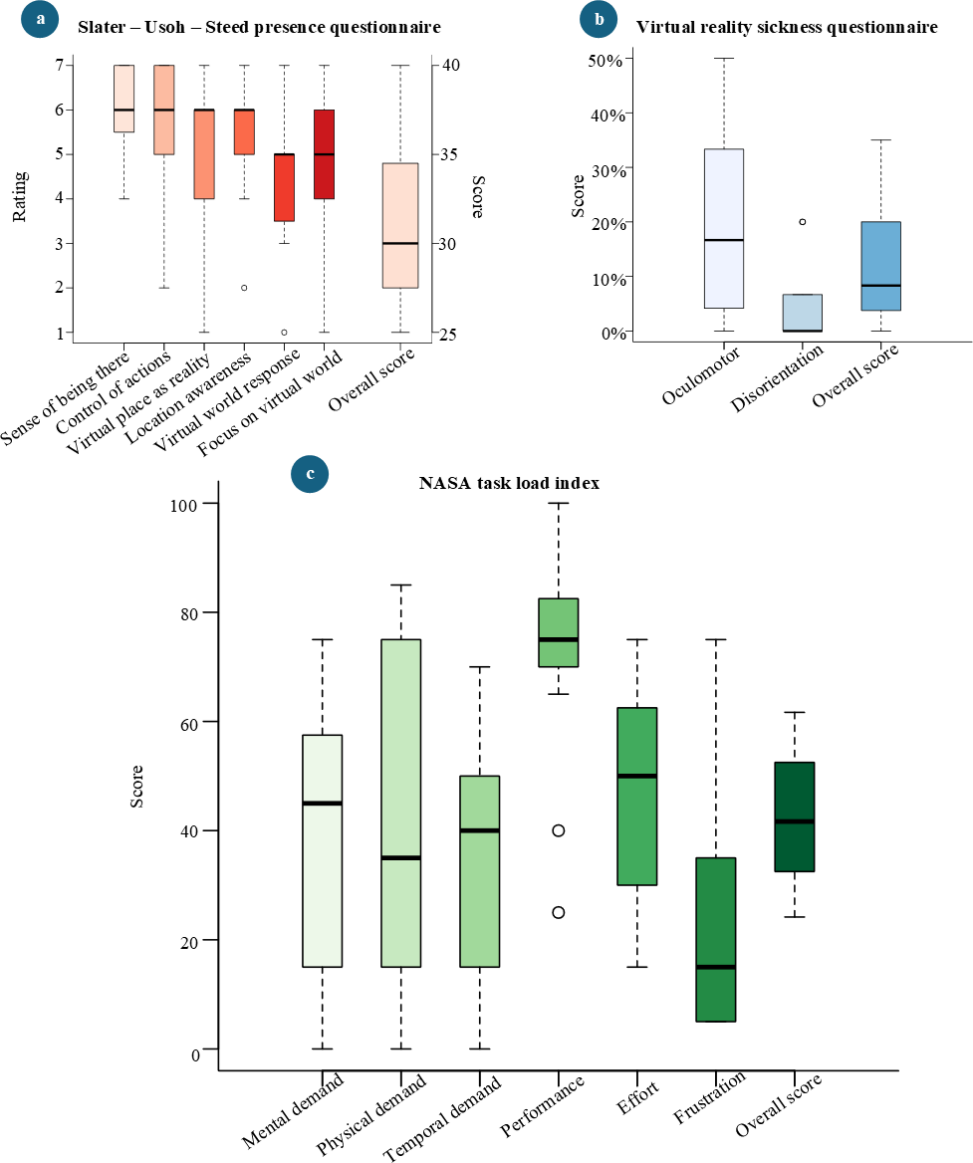
Distribution of answers for remaining questionnaires. (**A**) Slater-Usoh-Steed Questionnaire. (**B**) Virtual Reality Sickness Questionnaire. (**C**) NASA Task Load Index.

Participants’ responses to the SUS Questionnaire indicated varying levels of perceived presence across the six key aspects (Figure 11A). The question regarding whether the virtual environment felt like reality received a mean score of 6 (SD 0.93), suggesting a high sense of presence. For the question asking whether the environment felt more like a place visited than images seen, the mean was 5.73 (SD 1.49). Awareness of the real-world setting during the experience was rated at 4.93 (SD 1.71). Consistency between the virtual and real-world experiences was scored at 5.33 (SD 1.23). The question about whether the virtual environment overwhelmed participants’ senses had a mean score of 4.4 (SD 1.5), and for the question regarding whether the environment felt more like a place visited than observed, the mean was 4.8 (SD 1.7). Overall, the mean presence score across all 6 questions was 31.2 (SD 4.62), reflecting a moderate to high level of perceived presence.

#### Virtual Reality Sickness Questionnaire

Participants’ responses to the VRSQ indicated varying levels of symptoms (Figure 11B). The mean score for oculomotor-related symptoms was 17.78 (SD 16.63), suggesting moderate discomfort related to eye strain or visual issues. For disorientation symptoms, the mean score was lower, at 4.89 (SD 6.89), indicating relatively mild disorientation experiences. The overall mean score across all symptoms was 11.33 (SD 10.76), reflecting a low level of virtual reality sickness symptoms experienced by participants.

#### NASA Task Load Index

Participants reported varying levels of perceived workload across different task dimensions (Figure 11C). The mean score for mental demand was 36.67 (SD 24.90), while physical demand averaged 41.33 (SD 30.56), indicating moderate to high physical effort. Temporal demand, reflecting time pressure, had a mean score of 35.33 (SD 23.03). Participants rated their performance relatively high, with a mean score of 72.67 (SD 19.44). The effort required to complete tasks was rated at 47.33 (SD 19.81), and frustration levels were relatively low, with a mean score of 24.33 (SD 23.29). The overall workload, combining all dimensions, had a mean score of 42.95 (SD 11.96).

## Discussion

### Principal Findings

In this study, we incorporated VR illusions generated by offsetting the position of the playing hand within the VR table tennis game. Our selection of the VR illusions was primarily influenced by sensorimotor adaptation phenomena and other theories derived from it. In previous literature, it was highlighted that the users are able to adapt according to the modifications made to the embodiment in VR [47,48]. According to the rubber hand illusion theory, humans can be prompted to accept the rubber hand as their own even though there is a proprioceptive drift from the visual feedback [49-52]. Furthermore, humans have the ability rescale the perceptual-motor system to account for the mismatches and changes in the spatial variables [53-55]. Action-perception coupling was also considered when selecting the suitable VR illusion, as it explains brain’s ability to recalibrate the motions according to the feedback of the action [56-58]. This could also explain the fact that participants were able to recalibrate their body posture when they hit or missed the ball. Our hypothesis that posture recalibrations due to the encountered VR illusions could impact the CoP displacements was successfully proven through the obtained results.

#### Impact on Center of Pressure

CoP displacement was more pronounced during VR illusion stages compared with stages without illusions. Further analyses of CoP displacement revealed that the VR illusions had varying effects on absolute CoP displacements in both the mediolateral and anteroposterior directions.

In previous research, it was implied that the difficulty level and complexity of the VR gameplay could affect the balance performance [59,60]. However, this relationship remains insufficiently characterized. Participants exhibited higher absolute CoP displacements in both the mediolateral and anteroposterior directions when they were unsuccessful in returning the ball, compared with when they were successful (Figure 8). Participants were allowed to familiarize themselves with the game rules before the data collection. The experimenter ensured that each participant scored at least 5 consecutive hits before data collection began. As a result, the first 10 hits, representing the before illusion stage, were recorded with an almost 100% success rate across all participants. This suggests that the increased difficulty of gameplay introduced by the VR illusions could be the primary factor driving the differences in absolute CoP displacements before and during the VR illusion stages.

LMEs revealed that VR illusion magnitude is a significant main effect (*P*<.001) in increasing absolute CoP displacements in both the mediolateral and anteroposterior directions. In addition, the increase in illusion magnitude was significantly associated with a decrease in gameplay scores (*P*<.001). Figure 9A illustrates that the scores were higher during the low-magnitude level, indicating that participants found the low VR illusion magnitude more adaptable compared with the high magnitude level. Conversely, mean and maximum absolute CoP displacements in both mediolateral and anteroposterior directions were higher during high VR illusion magnitude levels compared with low magnitude levels (Figure 6). This observation further supports the inversely proportional relationship between gameplay performance (score) and absolute CoP displacement. Furthermore, it emphasizes that the difficulty level of the gameplay can be adjusted by changing the magnitude of the illusion. This finding would allow the therapists to control the level of perturbation effect on the patients in this type of interventions.

Post hoc tests revealed that the scores during anterior and posterior illusion directions differed significantly from the scores during medial and lateral directional VR illusions. Table 2 shows that the success rate of participants in returning the ball dropped to 38.3% and 55.4% during anterior and posterior directional VR illusions, respectively. Based on these observations and the relationship discussed earlier, we would expect the absolute CoP displacement during anterior and posterior directional illusions to be higher than during lateral and medial directional illusions. As anticipated, this pattern was observed in post hoc comparisons for both mean and maximum absolute anteroposterior CoP displacement (Figure 6).

However, this trend was only partially observed for mean and maximum absolute mediolateral CoP displacement. Significant differences were found between anterior and lateral directional illusions for both absolute mediolateral CoP displacement measures. In addition to that, significant differences were reported between anterior and medial directional illusions for maximum absolute mediolateral CoP displacement. The pronounced effect of anterior directional VR illusions may be explained by the fact that participants could easily perceive virtual hand offsets in the anterior direction during gameplay. In contrast, posterior directional VR illusions moved the virtual hand closer to the body, potentially causing the hand to be occluded from the participant’s field of view. As a result, participants may have shifted their body in the anteroposterior direction to see the hand properly and hit the ball. The intuitiveness of this adaptation might have caused the higher success percentage during the posterior illusions compared to the anterior illusions.

In this study, participants were instructed to keep their feet stationary, which may have hindered their ability to compensate for the lack of movement freedom when hitting the ball. However, some VR illusion types might have helped them to compensate for the lack of freedom for body movements. This could explain why no significant differences in absolute CoP displacements were observed between the VR illusion stages and the pre-illusion stages in the comparisons of absolute CoP displacement measures. This finding aligns with the higher scores reported for certain illusion types (Figure 9C), where the differences between before and during illusion stages were not statistically significant for the absolute CoP measures.

Higher F-statistics were reported for illusion direction in LMEs for absolute anteroposterior CoP displacements compared with those for absolute mediolateral CoP displacements, suggesting that directional illusions had a stronger impact on postural control in the anteroposterior direction. This difference could also account for the varying behaviors of absolute mediolateral and anteroposterior CoP measures, as depicted in Figures 4 and 5.

The directional tendencies of participants were analyzed to determine their movement preferences. Fisher exact test confirmed that the tendencies observed in Table 1 were not random but followed a statistically significant pattern. In addition, since all participants in this study were right-handed, it could be hypothesized that these directional tendencies would be mirrored in left-handed participants.

#### Immersive User Experience Considerations

In this study, we use the term “immersive user experience” to encompass the dimensions captured by the UEQ, SUS, NASA-TLX, and VRSQ questionnaires. Analysis of the UEQ responses showed that participants found the intervention attractive, easy to learn, easy to perform, engaging, and innovative. This was further confirmed by the moderate to high scores in both pragmatic and hedonic qualities. However, the dependability scale scored below average in the benchmark results, suggesting that participants may have found the task predictable. This predictability could be attributed to the consistent ball-feeding method used throughout the experiment.

The SUS presence questionnaire yielded moderate to high scores, indicating that participants felt a strong sense of presence within the VR environment. Crucially, they accepted the virtual hand offsets as part of their virtual body representation, recalibrating their behavior accordingly. Furthermore, moderate NASA-TLX scores, coupled with low overall VRSQ ratings, suggest that the intervention was manageable in terms of perceived workload and induced minimal virtual reality sickness symptoms, making the experience both feasible and user-friendly for participants.

In existing literature on VR illusions for balance, the focus has predominantly been on VR sickness as a user experience dimension [18], and the dimensions such as senses, emotions, and cognition of immersive user experience in VR-based balance interventions is often overlooked [17,61]. Consequently, it was uncertain whether participants would willingly engage in such interventions if asked. Our study’s moderate to high immersive user experience ratings suggest that participants are likely to prefer and engage with our intervention for extended periods.

To enhance our current intervention, future improvements could include diversifying ball feeding methods (eg, serve or dash) and varying ball serving directions. These modifications would address the predictable nature of the game, potentially improving scores on the dependability scale of the UEQ and increasing overall user engagement.

#### Implications for Standing Balance Rehabilitation Interventions

In balance rehabilitation interventions, CoP displacement is a crucial indicator of balance performance [4,62-64]. Effective balance rehabilitation often involves perturbing postural stability to stimulate improvements [65]. This study demonstrates that our VR-based intervention not only impacts CoP displacement but also allows for predictability in the direction of CoP movements based on VR illusion direction for some extent.

Our intervention enables controlled perturbation effects by adjusting the direction and magnitude of VR illusions, offering flexibility that could benefit clinical populations with compromised balance. In addition, the system requires only a safety harness, a VR HMD, and a VR game, making it a cost-effective and efficient alternative to traditional standing balance interventions, which often demand high therapist involvement. The simple setup allows for easy implementation in both clinical and home-based settings. With this flexibility and ease of implementation, therapists can tailor the VR intervention to design personalized rehabilitation paradigms that enhance engagement and address specific patient needs, potentially leading to improved balance outcomes.

Furthermore, throughout all the eight VR illusion based trials participants responded to the provide VR illusions unlike in some research in literature where participants neglected the VR illusions even though they initially responded to the illusions [19]. This sustained responsiveness suggests that our approach may overcome the sensory reweighting phenomenon observed in other studies, where visual dependency decreases over time as participants adapt to visual perturbations [66,67]. The consistency in participant responses also indicates that our VR paradigm effectively maintains sensorimotor engagement throughout the intervention period, potentially due to the ecological validity of our visual stimuli [68]. This finding aligns with research by Slater and colleagues [69], who demonstrated that contextually relevant visual perturbations sustain postural responses more effectively than abstract visual flows. In addition, the immersive nature of modern VR technology likely contributes to this sustained effect, as higher levels of presence have been correlated with stronger postural responses in virtual environments [70,71]. Future research should investigate the long-term sustainability of these responses during extended rehabilitation protocols, as Keshner and Kenyon [72] suggest that maintaining responsiveness to visual perturbations over multiple sessions is crucial for meaningful balance improvements in clinical populations.

The highest mean difference between the before and during illusion stages for mean absolute mediolateral CoP displacement was 4.4 cm, while it was 1.1 cm for mean absolute anteroposterior CoP displacement. These values suggest that individuals with compromised balance might experience significant postural challenges, potentially pushing their limits [73]. However, in a controlled virtual environment, these perturbations can be carefully managed, allowing users to safely engage with challenging balance tasks. The ability to adjust both the direction and magnitude of CoP displacement through VR illusions provides an opportunity to incrementally challenge a user’s postural control while mitigating the risk of overexertion or injury. Thus, our intervention offers a dynamic and adaptable platform for balance training. This adaptability is particularly beneficial for rehabilitation in clinical populations, allowing for gradual improvement in postural stability while minimizing excessive strain.

It is also important to note that some participants used the aid harness to maintain their balance during gameplay, indicating that the VR-induced perturbations posed a greater challenge for certain individuals. While the harness ensured safety, its use highlights the need to tailor the intervention to varying balance abilities. This safety feature allows for controlled difficulty adjustments, making the intervention suitable for a wider range of clinical populations. Over time, the goal would be to reduce reliance on external support as balance improves and confidence increases.

### Limitations and Future Directions

This study was conducted with a small but relatively balanced sample of 15 healthy young adults. While the findings may be generalizable within this demographic, caution should be taken when extending them to older adults or clinical populations. While our results provide valuable insights into VR-based balance rehabilitation interventions, future research should include diverse demographic groups, such as older adults and clinical populations, to explore confounding factors unique to these groups.

In addition to including a more diverse sample across different age groups and clinical backgrounds, future studies should investigate other aspects of lower limb rehabilitation. Specifically, the influence of VR illusions during treadmill walking should be explored, as treadmill-based rehabilitation is widely used in clinical settings. Furthermore, the feasibility of implementing home-based therapeutic interventions will be examined in future work.

### Conclusions

We proposed a novel approach to standing balance rehabilitation interventions by leveraging VR illusions to influence CoP displacement while maintaining a high level of immersive user experience. By systematically varying the direction and magnitude of the VR illusions, we successfully induced perturbations that challenged participants’ balance, leading to notable differences in CoP displacement across various illusion types. This innovative intervention not only impacted balance performance but also provided valuable insights into participants’ responsiveness to different VR conditions. Our findings indicate that the intervention was engaging, with participants reporting a moderate perceived workload, suggesting that the tasks were both manageable and stimulating. Minimal virtual reality sickness was observed, further confirming the feasibility of the intervention. In addition, participants experienced a moderate to high sense of presence, which is crucial for the effectiveness of VR-based interventions. Overall, this study supports the use of VR illusions as a dynamic and adaptable tool for standing balance rehabilitation interventions, offering a controlled yet enjoyable environment to progressively challenge and enhance postural stability. Furthermore, our insights into the relationships between game performance and balance performance, as well as the directional tendencies of CoP movements in response to illusion direction, provide a solid foundation for designing effective exergames for balance rehabilitation interventions.

## Supplementary material

10.2196/70376 Multimedia Appendix 1 Data analyses and related data files.

## Abbreviations

CoP: center of pressure
HMD: head mounted display
LME: linear mixed-effects model
NASA-TLX: NASA Task Load Index
SUS: Slater-Usoh-Steed
UEQ: User Experience Questionnaire
VR: virtual reality
VRSQ: Virtual Reality Sickness Questionnaire
