# Supplementary material for: Influence of Virtual Reality Illusions on Balance Performance and Immersive User Experience in Young Adults: A Within-Subject Experimental Study
Source: JMIR Serious Games. 2025 Jun 27;13:e70376. doi: 10.2196/70376 (PMC12226963; doi:10.2196/70376)
Supplement: Multimedia Appendix 1 [file games-v13-e70376-s001.zip › Multimedia Appendix/Data Files/Questionnaire_Data/VRSQ/VRSQ_Paper.pdf]

Applied Ergonomics 69 (2018) 66–73

Contents lists available at ScienceDirect

Applied Ergonomics

journal homepage: www.elsevier.com/locate/apergo

Virtual reality sickness questionnaire (VRSQ): Motion sickness measurement index in a virtual reality environment

Hyun K. Kim[a], Jaehyun Park[b,*], Yeongcheol Choi[b], Mungyeong Choe[b]

[a] Department of Industrial and Management Engineering, Pohang University of Science and Technology (POSTECH), Cheongam-ro 77, Nam-gu, Pohang, Gyeongbuk 37673, Republic of Korea

[b] Department of Industrial and Management Engineering, Incheon National University (INU), Academy-ro 119, Yeonsu-gu, Incheon 22012, Republic of Korea

ARTICLE INFO

Keywords:
Virtual reality
Motion sickness
Simulator sickness questionnaire

ABSTRACT

This study aims to develop a motion sickness measurement index in a virtual reality (VR) environment. The VR market is in an early stage of market formation and technological development, and thus, research on the side effects of VR devices such as simulator motion sickness is lacking. In this study, we used the simulator sickness questionnaire (SSQ), which has been traditionally used for simulator motion sickness measurement. To measure the motion sickness in a VR environment, 24 users performed target selection tasks using a VR device. The SSQ was administered immediately after each task, and the order of work was determined using the Latin square design. The existing SSQ was revised to develop a VR sickness questionnaire, which is used as the measurement index in a VR environment. In addition, the target selection method and button size were found to be significant factors that affect motion sickness in a VR environment. The results of this study are expected to be used for measuring and designing simulator sickness using VR devices in future studies.

1. Introduction

Virtual reality (VR) devices generate a virtual environment to replicate a real environment and provide users with a variety of experiences (Chryssolouris et al., 2000). The concept of VR was proposed several decades ago (Sutherland, 1968), and thus far, several studies have been conducted on VR (Mon-Williams et al., 1998; Stanney et al., 1998). A VR headset is a related technology that has recently become more widely available (Statista, 2016; Steed and Julier, 2013). The VR headset is a lightweight device that displays stereoscopic images to a user, thus creating the illusion of a 3D environment.

There are two major areas of research related to VR in the human–computer interaction (HCI) field: Hardware, to produce a more realistic environment using new technologies and functions (Bolton et al., 2014; Hwang et al., 2006; LaValle et al., 2014; Westerman et al., 2001), and theory, to develop and verify new types of interfaces and interactions in an implemented environment (Aslandere et al., 2015; de Haan et al., 2006; Jankowski and Hachet, 2015; Morimoto et al., 2007; Stanney et al., 2003a; Steed, 2006; Sun et al., 2015). With the rapid development of technology, new types and mechanisms of devices emerge frequently. Thus, interfaces and interactions that are considered optimal often change.

There have been many studies on motion sickness in VR environments (Dziuda et al., 2014; Kolasinski, 1995; Lo and So, 2001; Moss and Muth, 2011), and new technologies, which were not previously available, were applied to reduce motion sickness and develop interaction mechanisms and interfaces. For example, the resolution can be lowered or deliberately blurred, to reduce motion sickness and increase the sense of reality (Blum et al., 2010; Carnegie and Rhee, 2015).

Although there are studies on motion sickness in VR environments, there has been relatively little research on the motion sickness index for VR devices. The simulator sickness questionnaire (SSQ) (Balk et al., 2013; Kennedy et al., 1993) is used as a specialized method for measuring motion sickness for simulator systems; however, the SSQ may not be an ideal index for measuring VR sickness. There were significant differences between the simulators and VR systems in terms of the SSQ scores and the order of the contributions to total sickness (Drexler, 2006; Kennedy et al., 2010). They did not analyze a new index for measuring VR sickness; however, the results of previous studies demonstrated the importance of a new index for VR devices.

This study aims to develop and derive a VR sickness questionnaire (VRSQ) for use as a motion sickness measurement index specialized in VR environments. To develop VRSQ, the traditionally and widely used SSQ was modified to meet the VR environment criteria. In our experiment, 24 participants performed the target selection task using a VR headset and the SSQ was administered after each task. Two types of

* Corresponding author.
E-mail addresses: emokubi@postech.ac.kr (H.K. Kim), jaehpark@inu.ac.kr (J. Park), yc.choi@inu.ac.kr (Y. Choi), mgyeong@inu.ac.kr (M. Choe).

https://doi.org/10.1016/j.apergo.2017.12.016
Received 12 March 2017; Received in revised form 10 October 2017; Accepted 29 December 2017
Available online 16 January 2018
0003-6870/ © 2018 Elsevier Ltd. All rights reserved.

factor analysis were used: exploratory and confirmatory. In addition, the study also found two significant factors that affect motion sickness in VR environments using the analysis of variance (ANOVA) method—the target selection method and button size.

2. Simulator sickness questionnaire

A motion sickness questionnaire (MSQ) (Frank et al., 1983) was originally used to evaluate motion sickness in various forms of transport (e.g., cars, buses, ships, airplanes, etc.). Gianaros et al. (2001) identified four dimensions of motion sickness: gastrointestinal, central, peripheral, and sopite-related. Although the symptoms of simulator sickness are quite similar to those of motion sickness, they are usually much less severe. Therefore, there was a limit to evaluating the simulator sickness using MSQ. Lane et al. (1988) reported the SSQ, which is actively used in various fields as a simulator sickness measurement index, in a government document, which is available at the US Defense Documentation Center (DDC), and Kennedy et al. (1993) reported it in the open literature.

Kennedy et al. (1993) proposed a portion of the 28 MSQ items as the SSQ. By eliminating questionnaire items that overlapped sufficiently with other questionnaire items in the simulator sickness measurements, 16 items were retained in the SSQ. After factor analysis, the SSQ was divided into three categories: nausea, oculomotor, and disorientation (Table 1).

Formulas for scoring each component on the SSQ were also proposed (Table 2). Essentially, each SSQ item can have a score between 0 and 3. Thus, it is possible to obtain four SSQ scores: nausea, oculomotor, disorientation, and total.

The SSQ has been widely used to evaluate and reduce simulator sickness and to explore any significant effects (e.g., age, gender, equipment features, etc.) on simulator sickness. Previous studies have been conducted to measure and reduce simulator sickness for training devices (Dziuda et al., 2014), and to study simulator sickness caused by the field of view and image delay of head-up displays (Alshaer et al., 2017; Meehan et al., 2003). The SSQ is also used to provide an optimal virtual environment for head-mounted displays, which has recently received significant attention. Women experienced increased motion sickness than men in the VR system (Munafo et al., 2017) and increasing the root mean square velocity of visual oscillations while keeping the amplitude constant increased motion sickness (Chen et al., 2016). In addition to the work shown in Table 3, the SSQ was used to study motion sickness in the user interface of head-mounted displays.

Table 1
Symptoms in SSQ.

SSQ items	Nausea	Oculomotor	Disorientation
1. General discomfort	O	O	
2. Fatigue		O	
3. Headache		O	
4. Eyestrain		O	
5. Difficulty focusing		O	O
6. Increased salivation	O		
7. Sweating	O		
8. Nausea	O		O
9. Difficulty concentrating	O	O	
10. Fullness of head			O
11. Blurred vision		O	O
12. Dizzy (eyes open)			O
13. Dizzy (eyes closed)			O
14. Vertigo			O
15. Stomach awareness	O		
16. Burping	O		
Total	[1]	[2]	[3]

Table 2
Computation of SSQ scores.

SSQ components	Computation
Nausea	[1] × 9.54
Oculomotor	[2] × 7.58
Disorientation	[3] × 13.92
Total	([1] + [2] + [3]) × 3.74

Table 3
Previous studies of SSQ.

Authors	Devices		
	Simulator	HUD	HMD
Brooks et al. (2013)	O		
Moss and Muth (2011)		O	
Carnegie and Rhee, 2015			O
Jang et al. (2002)			O
Webb et al. (2009)	O		

3. Case study

3.1. Experimental design

3.1.1. Participants

The study group consisted of 12 female and 12 male participants (average age: 23 years; standard deviation: 1.2 years) with no physical or visual health problems. They were recruited through a university's intranet. All the participants were Asian and they were given an incentive to encourage participation. 21 of these participants who wore glasses removed them during the experiments, and they had no visual difficulties performing the VR tasks without the glasses. Among all participants, 6 had previous experience in using VR, and 3 owned a VR device. Thus, this was an experimental group with little experience using VR.

3.1.2. Apparatus

The VR environment was configured using a Samsung Gear VR (SM-R322NZWAXAR) and Samsung Galaxy S7. There are several versions of Gear VR, and the device used in this experiment is an early version with a field of view of 96°. The resolution of the screen was 1280 × 1440 for each eye. The head movement was tracked using the smartphone's inertial measurement unit (IMU) sensor, and the contents on the screen was changed to reflect the head movement. The motion-to-photon (MTP) latency was less than 20 ms.

To measure VR sickness, VR headsets were used to perform typical target selection tasks. The first method involves direct target selection by physically pressing the touch button on the right side of the headset when the cursor is over the desired object. The second method involves automatic target selection by gazing at the target for a certain length of time, when the cursor is over the desired object. Both methods used the head-tracking signal to move the cursor.

3.1.3. Tasks

VR sickness was measured considering target selection tasks widely used in VR headsets. The target selection task consists of repeatedly selecting a specific button using the cursor. A total of nine buttons are present for the panel in the form of a 3 × 3 array. The diagonal field of view of the panel was set as 27° 7′ 12″ considering two conditions: 1) a 32.2–36.2 cm distance from the eyes to the device, which is common in everyday mobile phone use (Bababekova et al., 2011), and 2) 14.4 × 7.3 cm (length × width), which is the mean smartphone size in South Korea (Lee et al., 2016) (Fig. 1).

Two sizes were used for the buttons. Small buttons were based on

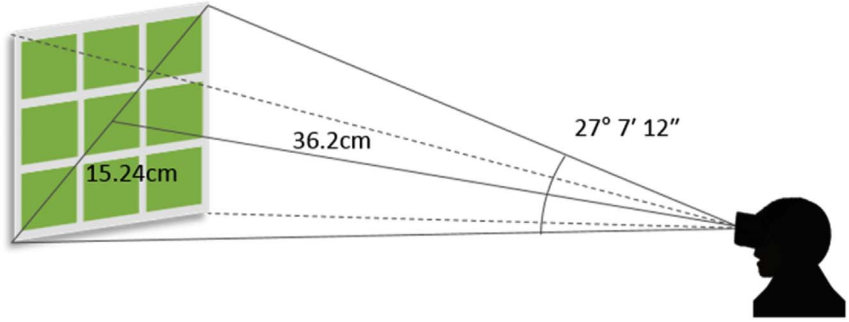

Fig. 1. Field of view considered during the experiment.

the length of the large side of the 3 × 4 keyboard on the smartphone or feature phone, setting the field of view as 1° 55′ 4”. In addition, large buttons were used to check the performance differences between both button sizes. The large buttons were set at twice the size of the small buttons to avoid any selection difficulty. The large buttons field of view was set as 3° 49′ 48″.

3.1.4. Procedure

Participants were asked to complete the SSQ by performing tasks consisting of two selection methods (i.e., direct and automatic selection) and two button sizes (i.e., large buttons and small buttons). Experiments were conducted on applications implemented using Samsung Gear VR equipped with Galaxy S7 smartphones.

The experiment lasted approximately 90 min, including break time. The procedures of the specific experiment were as follows.

First, the purpose and contents of the experiment were introduced and the SSQ instruments were explained. The informed consent was also explained to the participants, including the fact that 1) the questionnaire is anonymous, 2) the experiment will last approximately 90 min, 3) the purpose of the experiment is to measure motion sickness, therefore, the participants can take a rest whenever they want to, and 4) participants can finish the experiment freely when they feel severe motion sickness.

Second, participants performed tasks consisting of the four treatment conditions mentioned above (2 selection methods × 2 button sizes = 4 treatment conditions). Each treatment condition consisted of ten sets, and one set consisted of selecting randomly highlighted buttons four times; therefore, a total of 160 button selections occurred (4 treatment conditions x 4 random button selections x 10 sets = 160 tasks). To minimize the effect of motion sickness from the previous treatment condition on the next treatment condition, the participants were given a 2-min break between each treatment condition. The reason for the break time is that motion sickness has often been reported to be severe as the exposure duration increases (Stanney et al., 2003b), and has been remarkably reported to be severe after using the VR device for a maximum of 15 min (Munafo et al., 2017).

To minimize the effect of the treatment condition order, the Latin-square design was used. After each treatment condition, the participants evaluated motion sickness through the SSQ using a 4-point Likert scale (0 = not at all, 1 = slightly, 2 = moderately, and 3 = very).

Finally, when the experiment was completed, a debriefing questionnaire was administered.

3.2. Results

The SSQ profiles for the VR device was analyzed (Fig. 2). The SSQ proportional scores of nausea, oculomotor, and disorientation were 0.18, 0.39, and 0.42, respectively. Among the three categories (nausea, oculomotor, disorientation), the SSQ proportional score of nausea was the lowest.

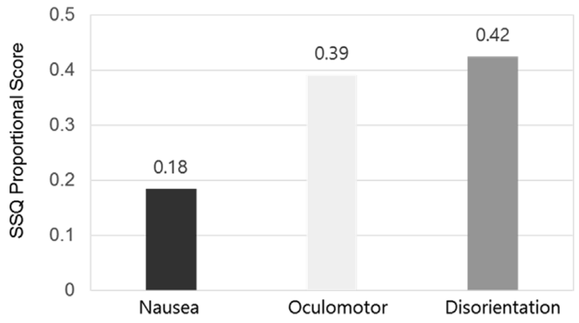

Fig. 2. SSQ profiles for VR device.

3.2.1. ANOVA with SSQ

Using the results of the above experiment, the SSQ was scored in terms of nausea, oculomotor, disorientation, and total score. ANOVA analysis was performed to determine whether participants were grouped according to selection methods or button sizes (Table 4).

Significant differences were found in all scores for nausea, oculomotor, disorientation, and total according to the button sizes and selection methods (α = 0.05). In Figs. 3 and 4, the four scores of the SSQ were arranged into two groups according to the selection methods and button sizes. The level of motion sickness was significantly lower for direct selection and larger buttons.

3.2.2. Exploratory and confirmatory factor analysis

The existing SSQ is a motion sickness measurement method designed in 1993, when virtual environments were most commonly generated using a simulator. Therefore, the method for measuring motion sickness in VR headsets should be revised. In this study, an SSQ was verified based on the data obtained from the experiment and was modified to suit the VR environment. An exploratory factor analysis was conducted to reconstruct the questionnaire items and a confirmatory factor analysis was used to verify the supplementary questionnaire items.

The identity matrix test of Kaiser–Meyer–Olkin (KMO) and Bartlett was performed. The KMO value showed that the total correlation matrix of the data performing the factor analysis is suitable for that analysis. Data is considered suitable for factor analysis if it is greater than 0.5. The Bartlett value, which indicates the significance of the

Table 4
Results of ANOVA on SSQ scores.

SSQ Component	Selection method	Button size
Nausea	*0.008	*0.006
Oculomotor	*0.001	*0.007
Disorientation	*0.003	*0.049
Total	*0.001	*0.01

*p < .05.

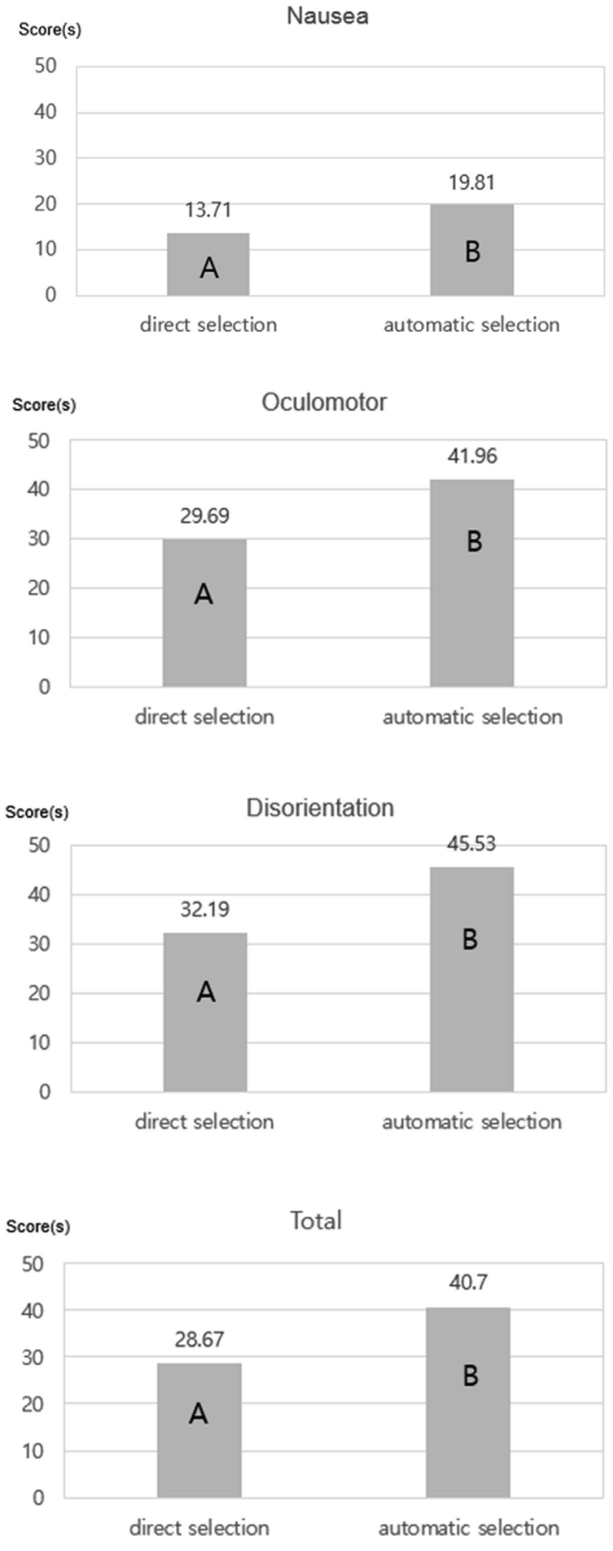

Fig. 3. SSQ scores for each dimension according to selection methods (Different letters indicate a statistically significant difference).

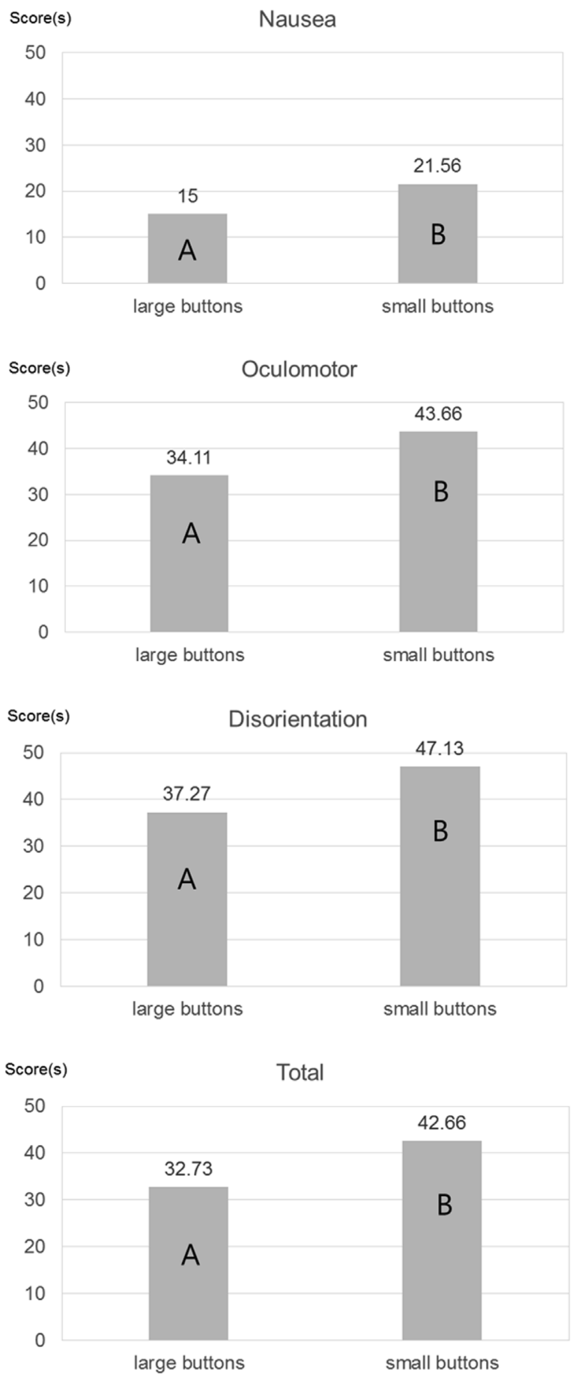

Fig. 4. SSQ scores for each dimension according to button sizes (Different letters indicate a statistically significant difference).

correlation matrix, is considered suitable for factor analysis if it is significant (Snedecor and Cochran, 1989). In this study, the KMO value was “0.869” and the Bartlett's significance was “0.001.”

Based on the above results, exploratory factor analysis was performed (Costello and Osborne, 2005) using the principal component analysis method based on the Kaiser rule (Kaiser, 1960), along with varimax rotation. Duplicated factors and factors with less than 0.5 factor loadings were removed for each component. Finally, if the number of factors included in the result from remaining factors by the preceding elimination criteria was less than 2, the corresponding component was removed. Thus, as shown in < Table 5 > , the number of items was reduced from 16 to 10. In addition, the conventional three components of nausea, oculomotor, and disorientation were reduced to two components.

Two components were named as oculomotor and disorientation. The oculomotor component consists of general discomfort (item 1), fatigue (item 2), eye strain (item 4), and difficulty in focusing (item 5). The disorientation component consists of headache (item 3), fullness of head (item 10), blurred vision (item 11), dizziness with eyes open (item 12), dizziness with eyes closed (item 13), and vertigo (item 14). Item 3

Table 5
Principle component analysis.

SSQ Items	Component 1	Component 2	Cronbach alpha
ITEM 1	0.71	0.39	0.847
ITEM 2	0.798	0.232	
ITEM 4	0.878	0.228	
ITEM 5	0.687	0.373	
ITEM 3	0.289	0.771	0.886
ITEM 10	0.165	0.86	
ITEM 11	0.279	0.649	
ITEM 12	0.451	0.701	
ITEM 13	0.306	0.742	
ITEM 14	0.331	0.729	

was the only factor for which the third component was included. Cronbach-α was 0.847 for the oculomotor axis and 0.886 for the disorientation axis. A value of 0.7 or higher indicates that internal consistency was sufficient (Cronbach, 1951).

Finally, the verification process was conducted using confirmatory factor analysis. In this process, dizziness with eyes open (item 12) was deleted from the disorientation component due to a high covariance value. Compared to the existing SSQ, the number of components was two and the number of items was nine. To verify the fit of the derived model, confirmatory factor analysis results are shown in Table 6. Results showed that all criteria were met.

The resulting VR sickness questionnaire (VRSQ) is shown in Table 7. In the VRSQ, the nausea component in SSQ was eliminated, and the 16 items in the SSQ were reduced to 9. Unlike the existing SSQ, one item was included only in one component as opposed to placing questionnaire items in several components. Table 8 shows the formula for calculating the VRSQ score and the total score of the oculomotor and disorientation components, which are components of the VRSQ. A simple averaging method was used for each questionnaire.

3.2.3. *ANOVA with VRSQ*

We derived the VRSQ scores from the data collected from these experiments. Analysis of variance (ANOVA) was performed to determine whether there was a significant difference between the two components of the VR target selection method and button size. Hence, apart from the disorientation score as per the button size as in < Table 9 > , there was a marked difference at the significance level $\alpha = 0.05$. In Fig. 5, the three scores of the SSQ were arranged into two groups according to the selection methods. In Fig. 6, the scores of the oculomotor and total motion sickness were arranged into two groups according to the button sizes. There were no significant differences in the disorientation score according to the button sizes.

4. Discussion

4.1. *Virtual reality sickness questionnaire (VRSQ)*

In this study, we attempted to measure motion sickness in a VR environment. As VR devices become popular, many studies on the development and verification of interfaces and interactions have been conducted, but research on motion sickness in VR is relatively limited; specifically, there are few viable tools for measuring motion sickness in a VR environment. SSQ, which has been used in existing simulator environments, is used to measure motion sickness that occurs in VR environments; however, some items in the SSQ are not relevant for measuring sickness in a VR environment.

Table 6
Confirmatory factor analysis.

Model fit	Criteria	Value
CMIN/DF	< 5	3.523
CFI	> 0.8	0.927
GFI	> 0.95	0.91
AGFI	> 0.8	0.844
RMSEA	< 0.1	0.115

Table 7
Virtual reality sickness questionnaire (VRSQ).

VRSQ symptom	Oculomotor	Disorientation
1. General discomfort	O	
2. Fatigue	O	
3. Eyestrain	O	
4. Difficulty focusing	O	
5. Headache		O
6. Fullness of head		O
7. Blurred vision		O
8. Dizzy (eyes closed)		O
9. Vertigo		O
Total	[1]	[2]

Table 8
Computation score of VRSQ.

SSQ components	Computation
Oculomotor	([1]/12)*100
Disorientation	([2]/15)*100
Total	(Oculomotor score + Disorientation score)/2

Table 9
Results of ANOVA analysis on VRSQ scores.

SSQ component	Selection method	Button size
Oculomotor	* < 0.001	*0.004
Disorientation	*0.007	0.075
Total	*0.01	* < 0.001

*p < .05.

The most prominent feature of the proposed VRSQ is that it is comprised of two components, namely the oculomotor and disorientation components. The nausea component contributed less to motion sickness than the oculomotor and disorientation components in a previous study (Drexler, 2006), and the same trend (Fig. 3) was observed in this study. Based on this trend, the nausea component was eliminated in this study when we analyzed the VRSQ by carrying out the exploratory and confirmatory factor analysis.

This can also be explained by the principle of motion sickness. Motion sickness can be described as inconsistencies between what you feel and what you see (Reason, 1978), which can be placed into three categories: 1) what I felt but did not see, 2) what I saw but did not feel, and 3) what I felt but did not match.

SSQ indicates motion sickness due to the simulators, including inertial motions; thus, it corresponds to all the three reasons. On the other hand, motion sickness resulting from a VR environment corresponds to the second reason; something that is seen but not felt. This includes visually perceived motion in the absence of inertial motion (Kennedy et al., 2010).

Therefore, simulator sickness is a concept that includes VR sickness, and VR sickness can be regarded as a subset of simulator sickness. Thus, it can be inferred that there are some characteristics included in the existing simulator sickness item, which do not apply to the VR environment.

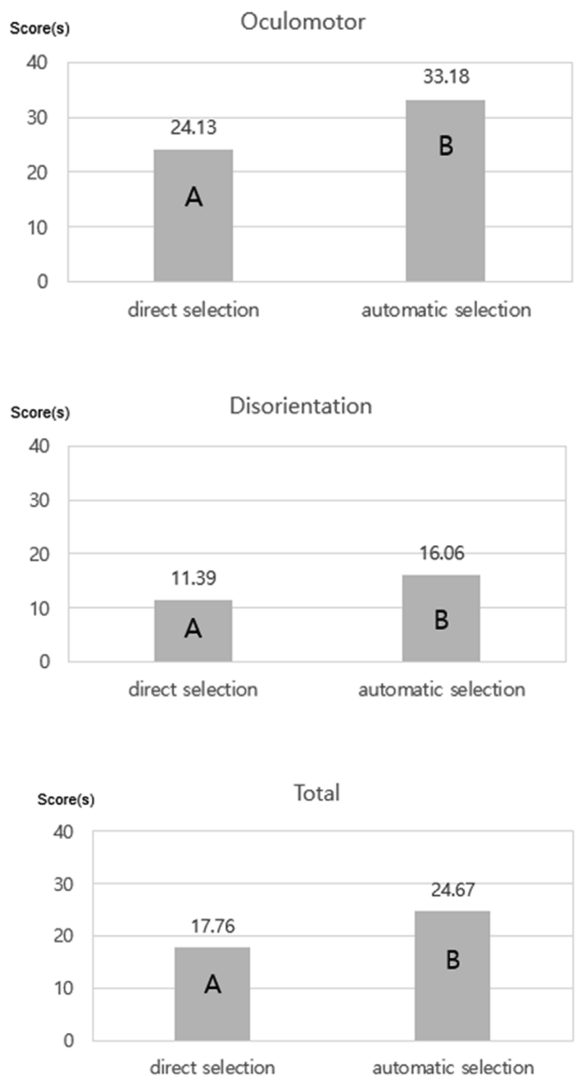

Fig. 5. VRSQ scores for each dimension according to selection methods (Different letters indicate a statistically significant difference).

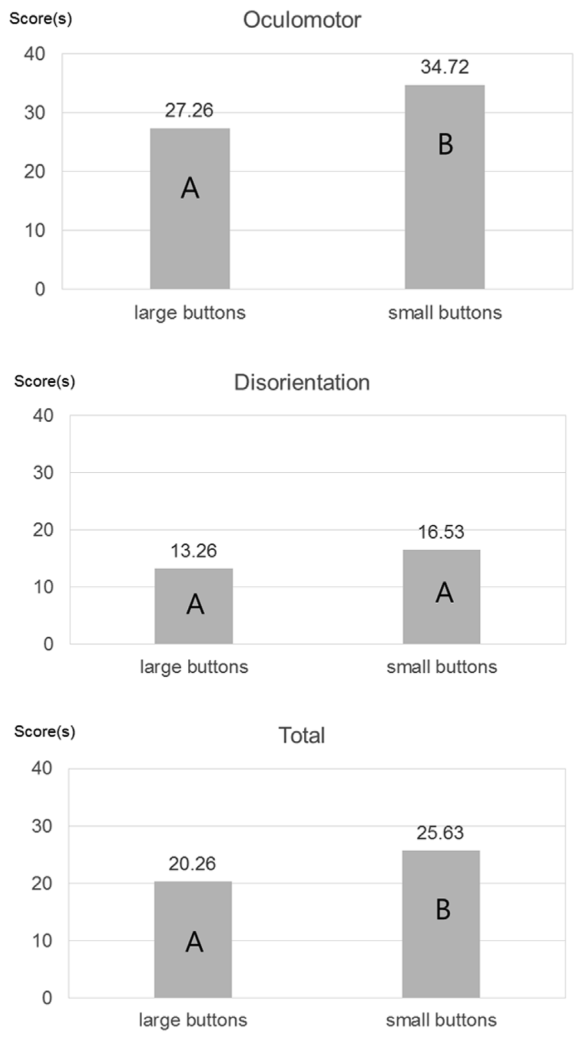

Fig. 6. VRSQ scores for each dimension according to button sizes (Different letters indicate a statistically significant difference).

4.2. Compatibility with SSQ

There are some differences between SSQ and VRSQ. VRSQ is a faster and more efficient questionnaire than SSQ as it contains fewer questionnaire items. In this study, VRSQ was derived to supplement SSQ; therefore, consistency between the two questionnaire results is an imperative. This is because if the results of both questionnaires are generally consistent, SSQ can be replaced with VRSQ; specifically, if the oculomotor and disorientation scores measured through the VRSQ are similar to those for the same component of the SSQ, then the SSQ can replace to VRSQ.

First, the ANOVA analysis results must be compared. It is possible to compare whether component scores in SSQ and VRSQ differ according to target selection methods and button size. In the case of the SSQ component, there were significant differences between all items. Alternatively, in the case of the VRSQ component, findings indicated that there was no significant difference in disorientation component concerning button size. In this study, a special task (i.e., target selection) was set up to search for new questionnaire items. This task is static and does not require sudden movement; these characteristics may have influenced results. For more precise analysis and confirmation, it is necessary to further test the task with rapid movement.

Second, we conducted correlation analysis based on the index scores derived from the two questionnaire items. Results showed that high numerical values of 0.952, 0.954, and 0.983 were obtained from the oculomotor, disorientation, and total component scores. Fundamentally, the two questionnaires appear to have a high correlation, reflecting the possibility of VRSQ as a viable alternative. Note that it is necessary to ensure that the existing SSQ is also extracted from the existing MSQ questionnaire. Where the VRSQs of this study were extracted from the SSQ, the SSQ was also extracted from the MSQ (Kennedy et al., 1993). The SSQ extracted the various types of motion sickness items from the MSQ related to the simulator, and the VRSQ extracted the types of motion sickness items from SSQ related to the VR headset.

4.3. Target selection methods in a VR environment

In this study, motion sickness in a VR environment was measured by changing the target selection method (i.e., direct selection, and automatic selection) and button size. Using both SSQ and VRSQ, it was discovered that these two factors (target selection and button size) affect motion sickness overall.

First, there was less motion sickness when using direct selection method compared to automatic selection. This indicates that a gaze itself affects the increase in motion sickness. This may be due to the actual length of time of the gaze, or the method itself may cause motion sickness.

Second, the button size was also a significant factor in influencing

motion sickness in a VR environment. Although this is specified by the button size, it can be generalized to the degree of difficulty of the target selection task; the more difficult the task, the more motion sickness occurs. This is consistent with a 2010 2D study, which found that difficulty affects usability (Park and Han, 2010). Thus, the better the usability, the less motion sickness can be analyzed. Further research must be conducted for generalization.

4.4. Limitations of VRSQ

There are some limitations to this model and the VRSQ proposed in this study. First, there is a lack of participants. For a very limited number of factors, experiments were conducted with the minimum number participants necessary to generalize the results of the factor analysis (Preacher and MacCallum, 2002). However, the mathematical analysis is sound, and it is significant enough to emphasize that the SSQ requires supplementation in a VR environment, and to suggest the procedure for developing the motion sickness measurement tool for VR. Note that this study used equal weights while combining the oculomotor and disorientation dimensions; further research can be conducted to extract accurate coefficients for the total score.

Second, the explanation of the deletion of the nausea component from the existing SSQ is not sufficient. SSQ is a motion sickness measurement tool designed for a simulator environment and may not be effective in a VR environment. That does not mean that the nausea component should be removed from the VR. However, when we summarize the interviews and the mathematical analyses of the participants' responses, it raises the question that the nausea component in the existing SSQ may pose a problem, as two or more axes are mixed. In the nausea component, cognitive reactions such as stomach awareness or burping, and physiological reaction such as difficulty concentrating were mixed. The discussion on this should be expanded in future research.

Third, various control tasks must be added to future studies. In this study, a simple control task with variation in target selection methods and button sizes was performed. Even if numerous input tasks occur, it is necessary to include more diverse tasks in the experiment as motion sickness may be caused simply listening music or watching the 360° video. In future research, we plan to reaffirm motion sickness in a VR environment while focusing on content viewing.

5. Conclusion

In this study, we attempted to verify and supplement an SSQ, which was used as a motion sickness measurement tool by using simple control tasks in a VR environment. The existing SSQ consisted of 16 items, and these items are included in three components of nausea, oculomotor, and disorientation. In this study, our newly derived VRSQ consisted of 9 items, which are included in two components, namely oculomotor and disorientation.

Experiments in a simple VR environment were performed to propose and verify the VRSQ. A total of 24 participants performed simple control tasks consisting of 9 buttons in the VR environment. In this process, there were two different button sizes, and two types of target selection methods; direct selection and automatic selection.

The proposed VRSQ is assumed to replace the SSQ to some extent, and the results of correlation analysis showed a high correlation between SSQ and VRSQ. The results of the ANOVA analysis are also very similar. VRSQ is highly recommended over SSQ in the VR environment, as the existing SSQ includes items not related to the VR environment, and uses more questionnaire items which reduce the efficiency of the tool.

In future studies, VRSQ could be updated through further experiments. Generalization is limited, however, as the number of participants was relatively small and the tasks performed in the experiment were constrained. Further discussion and experiments are needed to provide a deeper rationale for the elimination of nausea. The result of this study is expected to be used as a guideline for developing and delivering content when considering new interfaces in a VR environment.

Acknowledgments

Many thanks authors who invented SSQ (Kennedy, R.S., Lane, N.E., Berbaum, K.S., Lilienthal, M.G.), and note that this work was supported by the National Research Foundation of Korea (NRF) grant funded by the Korea government (MSIP) (No. 2015R1C1A1A01054148).

References

Alshaer, A., Regenbrecht, H., O'Hare, D., 2017. Immersion factors affecting perception and behaviour in a virtual reality power wheelchair simulator. Appl. Ergon. 58, 1–12.
Aslandere, T., Dreyer, D., Pankratz, F., 2015. Virtual hand-button interaction in a generic virtual reality flight simulator. In: 2015 IEEE Aerospace Conference, pp. 1–8.
Bababekova, Y., Rosenfield, M., Hue, J.E., Huang, R.R., 2011. Font size and viewing distance of handheld smart phones. Optom. Vis. Sci. 88 (7), 795–797.
Balk, S.A., Bertola, M.A., Inman, V.W., 2013. Simulator sickness questionnaire: twenty years later. In: Proceedings of the Seventh International Driving Symposium on Human Factors in Driver Assessment, Training, and Vehicle Design.
Blum, T., Wieczorek, M., Aichert, A., Tibrewal, R., Navab, N., 2010. The Effect of Out-of-focus Blur on Visual Discomfort when Using Stereo Displays, 9th IEEE International Symposium on Mixed and Augmented Reality 2010. vol.2010. Science and Technology, ISMAR, pp. 13–17.
Bolton, J., Lambert, M., Lirette, D., Unsworth, B., 2014. PaperDude: a virtual reality cycling exergame. In: CHI'14 Extended Abstracts on Human Factors in Computing Systems, pp. 475–478.
Brooks, J.O., Goodenough, R.R., Crisler, M.C., Klein, N.D., Alley, R.L., Koon, B.L., Logan Jr., W.C., Ogle, J.H., Tyrrell, R.A., Wills, R.F., 2013. Simulator sickness during driving simulation studies. Accid. Anal. Prev. 42 (3), 788–796.
Carnegie, K., Rhee, T., 2015. Reducing visual discomfort with hmds using dynamic depth of field. IEEE Comput. Graph. Appl. 35 (5), 34–41.
Chen, D.J., Bao, B., Zhao, Y., So, R.H., 2016. Visually induced motion sickness when viewing visual oscillations of different frequencies along the fore-and-aft axis: keeping velocity versus amplitude constant. Ergonomics 59 (4), 582–590.
Chryssolouris, G., Mavrikios, D., Fragos, D., Karabatsou, V., 2000. A virtual reality-based experimentation environment for the verification of human-related factors in assembly processes. Robot. Comput. Integrated Manuf. 16 (4), 267–276.
Costello, A.B., Osborne, J.W., 2005. Best practices in exploratory factor analysis: four recommendations for getting the most from your analysis. Practical Assess. Res. Eval. 10 (7), 173–178.
Cronbach, L.J., 1951. Coefficient alpha and the internal structure of tests. Psychometrika 16 (3), 297–334.
Drexler, J.M., 2006. Identification of System Design Features that Affect Sickness in Virtual Environments. Doctoral Dissertation. University of Central Florida, Orlando Available at. http://stars.library.ucf.edu/cgi/viewcontent.cgi?article=1796&context=etd.
Dziuda, Ł., Biernacki, M.P., Baran, P.M., Truszczyński, O.E., 2014. The effects of simulated fog and motion on simulator sickness in a driving simulator and the duration of after-effects. Appl. Ergon. 45 (3), 406–412.
Frank, L.H., Kennedy, R.S., Kellogg, R.S., McCauley, M.E., 1983. Simulator sickness: a reaction to a transformed perceptual world. 1. Scope of the Problem (NAVTRAEQUIPCEN TN-65). Naval Training Equipment Center, Orlando, FL.
Gianaros, P.J., Muth, E.R., Mordkoff, J.T., Levine, M.E., Stern, R.M., 2001. A questionnaire for the assessment of the multiple dimensions of motion sickness. Aviat Space Environ. Med. 72 (2), 115–119.
de Haan, G., Griffith, E.J., Koutek, M., Post, F.H., 2006. Hybrid interfaces in VEs: intent and interaction. In: EGVE, pp. 109–118.
Hwang, J., Jung, J., Kim, G.J., 2006. Hand-held virtual reality: a feasibility study. In: Proceedings of the ACM Symposium on Virtual Reality Software and Technology, pp. 356–363.
Jang, D.P., Kim, I.Y., Nam, S.W., Wiederhold, B.K., Wiederhold, M.D., Kim, S.I., 2002. Analysis of physiological response to two virtual environments: driving and flying simulation. Cyberpsychol. Behav. 5 (1), 11–18.
Jankowski, J., Hachet, M., 2015. Advances in interaction with 3d environments. In: Computer Graphics Forum. vol.34. pp. 152–190 No. 1.
Kaiser, H.F., 1960. The application of electronic computers to factor analysis. Educ. Psychol. Meas. 20, 141–151.
Kennedy, R.S., Lane, N.E., Berbaum, K.S., Lilienthal, M.G., 1993. Simulator sickness questionnaire: an enhanced method for quantifying simulator sickness. Int. J. Aviat. Psychol. 3 (3), 203–220.
Kennedy, R.S., Drexler, J., Kennedy, R.C., 2010. Research in visually induced motion sickness. Appl. Ergon. 41 (4), 494–503.
Kolasinski, E.M., 1995. Simulator Sickness in Virtual Environments (No. ARI-TR-1027). Army Research Inst. for the Behavioral and Social Sciences, Alexandria VA.
Lane, N.E., Kennedy, R.S., Lilienthal, M.G., 1988. Development of a New Scoring System for Measuring Simulator Sickness (Report No. NAVTRASYSCEN TR-87-009). Naval Training Systems Center, Orlando, FL.

LaValle, S.M., Yershova, A., Katsev, M., Antonov, M., 2014. Head tracking for the oculus rift. In: 2014 IEEE International Conference on Robotics and Automation (ICRA), pp. 187–194.
Lee, S., Kyung, G., Lee, J., Moon, S.K., Park, K.J., 2016. Grasp and index finger reach zone during one-handed smartphone rear interaction: effects of task type, phone width and hand length. Ergonomics 59 (11), 1462–1472.
Lo, W.T., So, R.H., 2001. Cybersickness in the presence of scene rotational movements along different axes. Appl. Ergon. 32 (1), 1–14.
Meehan, M., Razzaque, S., Whitton, M.C., 2003. Effect of latency on presence in stressful virtual environments. In: Proceedings of IEEE Virtual Reality.
Mon-Williams, M., Plooy, A., Burgess-Limerick, R., Wann, J., 1998. Gaze angle: a possible mechanism of visual stress in virtual reality headsets. Ergonomics 41 (3), 280–285.
Morimoto, K., Miyajima, C., Itou, K., Takeda, K., 2007. A virtual button interface using fingertip movements. In: 2007 International Conference on Machine Learning and Cybernetics, vol.4. pp. 2089–2093.
Moss, J.D., Muth, E.R., 2011. Characteristics of head-mounted displays and their effects on simulator sickness. Hum. Factors 53 (3), 308–319.
Munafo, J., Diedrick, M., Stoffregen, T.A., 2017. The virtual reality head-mounted display Oculus Rift induces motion sickness and is sexist in its effects. Exp. Brain Res. 235 (3), 889–901.
Park, Y.S., Han, S.H., 2010. Touch key design for one-handed thumb interaction with a mobile phone: effects of touch key size and touch key location. Int. J. Ind. Ergon. 40, 68–76.
Preacher, K.J., MacCallum, R.C., 2002. Exploratory factor analysis in behavior genetics research: factor recovery with small sample sizes. Behav. Genet. 32, 153–161.
Reason, J.T., 1978. Motion sickness adaptation: a neural mismatch model. J. Roy. Soc. Med. 71 (11), 819.
Snedecor, G.W., Cochran, W.G., 1989. Statistical Methods, eighth ed. Iowa State University Press, Ames, IA.
Stanney, K.M., Mourant, R.R., Kennedy, R.S., 1998. Human factors issues in virtual environments: a review of the literature. Presence 7 (4), 327–351.
Stanney, K.M., Mollaghasemi, M., Reeves, L., Breaux, R., Graeber, D.A., 2003a. Usability engineering of virtual environments (VEs): identifying multiple criteria that drive effective VE system design. Int. J. Hum. Comput. Stud. 58 (4), 447–481.
Stanney, K.M., Kingdon, K.S., Nahmens, I., Kennedy, R.S., 2003b. What to expect from immersive virtual environment exposure: influences of age, gender, body mass index, and past experience. Hum. Factors 45 (3), 504–520.
Statista, 2016. Virtual Reality Hardware Market Size Worldwide from 2016 to 2020. https://www.statista.com/statistics/550461/virtual-reality-market-size-worldwide/.
Steed, A., 2006. Towards a general model for selection in virtual environments. In: 3D User Interfaces (3DUI'06), pp. 103–110.
Steed, A., Julier, S., 2013. Design and implementation of an immersive virtual reality system based on a smartphone platform. In: 3D User Interfaces (3DUI), 2013 IEEE Symposium on, pp. 43–46.
Sun, H.M., Li, S.P., Zhu, Y.Q., Hsiao, B., 2015. The effect of user's perceived presence and promotion focus on usability for interacting in virtual environments. Appl. Ergon. 50, 126–132.
Sutherland, I.E., 1968. A head-mounted three dimensional display. In: Proceedings of the December 9-11, 1968, Fall Joint Computer Conference, Part I, pp. 757–764.
Webb, C.M., Bass, J.M., Johnson, D.M., Kelley, A.M., Martin, C.R., Wildzunas, R.M., 2009. Simulator sickness in a helicopter flight training school. Aviat Space Environ. Med. 80 (6), 541–545.
Westerman, S.J., Cribbin, T., Wilson, R., 2001. Virtual information space navigation: evaluating the use of head tracking. Behav. Inf. Technol. 20 (6), 419–426.
