# Supplementary material for: Influence of Virtual Reality Illusions on Balance Performance and Immersive User Experience in Young Adults: A Within-Subject Experimental Study
Source: JMIR Serious Games. 2025 Jun 27;13:e70376. doi: 10.2196/70376 (PMC12226963; doi:10.2196/70376)
Supplement: Multimedia Appendix 1 [file games-v13-e70376-s001.zip › Multimedia Appendix/Codes/Stats_of_Directional_Tendency.html]

Directionality\_Statistics


# Directionality\_Statistics

#### Achintha Abayasiri

#### 2024-12-20

---

```
# Load necessary library
library(tidyverse)
```

```
## ── Attaching core tidyverse packages ──────────────────────── tidyverse 2.0.0 ──
## ✔ dplyr     1.1.4     ✔ readr     2.1.5
## ✔ forcats   1.0.0     ✔ stringr   1.5.1
## ✔ ggplot2   3.5.1     ✔ tibble    3.2.1
## ✔ lubridate 1.9.3     ✔ tidyr     1.3.1
## ✔ purrr     1.0.2     
## ── Conflicts ────────────────────────────────────────── tidyverse_conflicts() ──
## ✖ dplyr::filter() masks stats::filter()
## ✖ dplyr::lag()    masks stats::lag()
## ℹ Use the conflicted package (<http://conflicted.r-lib.org/>) to force all conflicts to become errors
```

This is the best package for reading data into R it is incredibly
fast

```
library(data.table)
```

```
## 
## Attaching package: 'data.table'
```

```
## The following objects are masked from 'package:lubridate':
## 
##     hour, isoweek, mday, minute, month, quarter, second, wday, week,
##     yday, year
```

```
## The following objects are masked from 'package:dplyr':
## 
##     between, first, last
```

```
## The following object is masked from 'package:purrr':
## 
##     transpose
```

## Setting the Working Directory and Reading the Data

First, we need to make sure R can find the data file

```
setwd("Z:/Data_Collection/Study_1/Participant_Data/Biomechanics_Data/Directionality")

# Initialize an empty list to store data frames
data_list <- list()

for (i in 0:8) {
  dummy_name <- paste0("CoP_Data_All_Directional_Means_test", i)
  file_name <- paste0(dummy_name, ".csv")
  data <- fread(file_name)
  # Check if necessary columns exist
  if (!all(c("Mean_Aft_ML", "Mean_Aft_AP") %in% names(data))) {
    stop(paste("Missing necessary columns in", file_name))
  }
  data_list[[i+1]] <- data
}
```

```
# Combine all data.tables in the list into one data.table
combined_data <- rbindlist(data_list, use.names = TRUE, fill = TRUE)

# View the combined data frame
str(combined_data)
```

```
## Classes 'data.table' and 'data.frame':   135 obs. of  6 variables:
##  $ Participant_Number: chr  "PS1F03" "PS1M04" "PS1M05" "PS1M06" ...
##  $ Mean_Aft_ML       : num  -0.1743 0.497 0.3403 0.0175 0.5922 ...
##  $ Mean_Aft_AP       : num  0.00855 0.68705 0.10967 0.22108 0.35199 ...
##  $ Illusion          : chr  "Neutral" "Neutral" "Neutral" "Neutral" ...
##  $ Quadrant          : chr  "Q2" "Q1" "Q1" "Q1" ...
##  $ Magnitude         : chr  "None" "None" "None" "None" ...
##  - attr(*, ".internal.selfref")=<externalptr>
```

```
# Make Quadrant and Illusion as factors
combined_data$Quadrant <- as.factor(combined_data$Quadrant)
combined_data$Illusion <- as.factor(combined_data$Illusion)
str(combined_data)
```

```
## Classes 'data.table' and 'data.frame':   135 obs. of  6 variables:
##  $ Participant_Number: chr  "PS1F03" "PS1M04" "PS1M05" "PS1M06" ...
##  $ Mean_Aft_ML       : num  -0.1743 0.497 0.3403 0.0175 0.5922 ...
##  $ Mean_Aft_AP       : num  0.00855 0.68705 0.10967 0.22108 0.35199 ...
##  $ Illusion          : Factor w/ 5 levels "Anterior","Lateral",..: 4 4 4 4 4 4 4 4 4 4 ...
##  $ Quadrant          : Factor w/ 4 levels "Q1","Q2","Q3",..: 2 1 1 1 1 1 2 2 4 1 ...
##  $ Magnitude         : chr  "None" "None" "None" "None" ...
##  - attr(*, ".internal.selfref")=<externalptr>
```

```
# Create a contingency table
contingency_table <- table(combined_data$Illusion, combined_data$Quadrant)

# Print Contingency Table
print(contingency_table)
```

```
##            
##             Q1 Q2 Q3 Q4
##   Anterior   7  2 14  7
##   Lateral    7 12  9  2
##   Medial    11  4  2 13
##   Neutral    8  4  0  3
##   Posterior 13  5  8  4
```

```
# Perform chi-squared test
chi_squared_test <- chisq.test(contingency_table)
```

```
## Warning in chisq.test(contingency_table): Chi-squared approximation may be
## incorrect
```

```
# Set seed for reproducibility
set.seed(12345)

# Perform Fisher's Exact Test using Monte Carlo simulation with more replicates
fisher_test <- fisher.test(contingency_table, simulate.p.value = TRUE, B = 1e6)

# Print the test results
print(chi_squared_test)
```

```
## 
##  Pearson's Chi-squared test
## 
## data:  contingency_table
## X-squared = 39.081, df = 12, p-value = 0.000102
```

```
print(fisher_test)
```

```
## 
##  Fisher's Exact Test for Count Data with simulated p-value (based on
##  1e+06 replicates)
## 
## data:  contingency_table
## p-value = 7.5e-05
## alternative hypothesis: two.sided
```
