# Supplementary material for: Influence of Virtual Reality Illusions on Balance Performance and Immersive User Experience in Young Adults: A Within-Subject Experimental Study
Source: JMIR Serious Games. 2025 Jun 27;13:e70376. doi: 10.2196/70376 (PMC12226963; doi:10.2196/70376)
Supplement: Multimedia Appendix 1 [file games-v13-e70376-s001.zip › Multimedia Appendix/Codes/State_Comparisons.html]

State Comparisons


# State Comparisons

#### Achintha Abayasiri

#### 2024-12-20

## Loading the required libraries

```
if(!require(tidyr)) install.packages("tidyr")
```

```
## Loading required package: tidyr
```

```
library(tidyr)
```

This is the best package for reading data into R it is incredibly
fast

```
library(data.table)
```

This is a great package for summarizing data into tables

```
library(dplyr)
```

This is a great package for nice colors

```
library(RColorBrewer)
```

Run libraries for LME, note that you have to download first time
only

```
library(nlme)
require(multcomp)
library(lattice)
```

Other required Packages

```
library(tidyverse)
library(ggsignif)
```

## Setting the Working Directory and Reading the Data

First, we need to make sure R can find the data file

```
setwd("Z:/Data_Collection/Study_1/Participant_Data/Biomechanics_Data")
ex <- fread('CoP_Data_All_fields.csv')
```

This copy paste is only if function below does work

***ex<-read.table(pipe(“pbpaste”),header=T)***

‘fread’ is the function to read data into R using data.table

## Imported Data

Lets check we’ve read the data in properly

```
head(ex)
```

```
##    Participant_Number State_Number   Ill_Dir Ill_Mag B4_AP_Abs_Mean
##                <char>        <int>    <char>  <char>          <num>
## 1:             PS1F03            1  Anterior     Low      0.3284532
## 2:             PS1F03            2  Anterior    High      0.3036768
## 3:             PS1F03            3    Medial     Low      0.2597069
## 4:             PS1F03            4    Medial    High      0.5000495
## 5:             PS1F03            5 Posterior     Low      0.2958926
## 6:             PS1F03            6 Posterior    High      0.2925373
##    B4_ML_Abs_Mean Aft_AP_Abs_Mean Aft_ML_Abs_Mean B4_AP_Max B4_ML_Max
##             <num>           <num>           <num>     <num>     <num>
## 1:      0.2007189       0.6382157       0.4382867 0.8484953 0.6898747
## 2:      0.2667744       1.3080774       0.4977725 1.0183512 0.9638926
## 3:      0.1619572       0.3789147       0.2130429 0.8612730 0.6724250
## 4:      0.2734911       1.1413778       2.4666020 1.7640084 1.0529368
## 5:      0.2519057       1.2998413       0.9411277 0.8229062 0.9477333
## 6:      0.2879892       1.3399095       2.1865327 1.3827584 1.2222034
##    Aft_AP_Max Aft_ML_Max AP_Abs_Mean_All ML_Abs_Mean_All AP_Abs_Std_All
##         <num>      <num>           <num>           <num>          <num>
## 1:   2.427312  1.2572671       0.5497005       0.3704013      0.5497005
## 2:   6.212424  2.4954484       1.0210682       0.4317644      1.0210682
## 3:   1.324807  0.9828079       0.3448508       0.1984451      0.3448508
## 4:   2.929541  4.3602468       0.9581171       1.8399168      0.9581171
## 5:   3.429201  4.1016492       1.0129613       0.7441813      1.0129613
## 6:   4.218612  7.2963612       1.0406211       1.6440206      1.0406211
##    ML_Abs_Std_All
##             <num>
## 1:      0.3704013
## 2:      0.4317644
## 3:      0.1984451
## 4:      1.8399168
## 5:      0.7441813
## 6:      1.6440206
```

Checking internal structure of data frames.

```
str(ex)
```

```
## Classes 'data.table' and 'data.frame':   120 obs. of  16 variables:
##  $ Participant_Number: chr  "PS1F03" "PS1F03" "PS1F03" "PS1F03" ...
##  $ State_Number      : int  1 2 3 4 5 6 7 8 1 2 ...
##  $ Ill_Dir           : chr  "Anterior" "Anterior" "Medial" "Medial" ...
##  $ Ill_Mag           : chr  "Low" "High" "Low" "High" ...
##  $ B4_AP_Abs_Mean    : num  0.328 0.304 0.26 0.5 0.296 ...
##  $ B4_ML_Abs_Mean    : num  0.201 0.267 0.162 0.273 0.252 ...
##  $ Aft_AP_Abs_Mean   : num  0.638 1.308 0.379 1.141 1.3 ...
##  $ Aft_ML_Abs_Mean   : num  0.438 0.498 0.213 2.467 0.941 ...
##  $ B4_AP_Max         : num  0.848 1.018 0.861 1.764 0.823 ...
##  $ B4_ML_Max         : num  0.69 0.964 0.672 1.053 0.948 ...
##  $ Aft_AP_Max        : num  2.43 6.21 1.32 2.93 3.43 ...
##  $ Aft_ML_Max        : num  1.257 2.495 0.983 4.36 4.102 ...
##  $ AP_Abs_Mean_All   : num  0.55 1.021 0.345 0.958 1.013 ...
##  $ ML_Abs_Mean_All   : num  0.37 0.432 0.198 1.84 0.744 ...
##  $ AP_Abs_Std_All    : num  0.55 1.021 0.345 0.958 1.013 ...
##  $ ML_Abs_Std_All    : num  0.37 0.432 0.198 1.84 0.744 ...
##  - attr(*, ".internal.selfref")=<externalptr>
```

```
state_mapping <- c(
  "1" = "Anterior-Low",
  "2" = "Anterior-High",
  "3" = "Medial-Low",
  "4" = "Medial-High",
  "5" = "Posterior-Low",
  "6" = "Posterior-High",
  "7" = "Lateral-Low",
  "8" = "Lateral-High"
)

# Update State_Number with the character labels
ex[, State_Number := state_mapping[as.character(State_Number)]]

str(ex)
```

```
## Classes 'data.table' and 'data.frame':   120 obs. of  16 variables:
##  $ Participant_Number: chr  "PS1F03" "PS1F03" "PS1F03" "PS1F03" ...
##  $ State_Number      : chr  "Anterior-Low" "Anterior-High" "Medial-Low" "Medial-High" ...
##  $ Ill_Dir           : chr  "Anterior" "Anterior" "Medial" "Medial" ...
##  $ Ill_Mag           : chr  "Low" "High" "Low" "High" ...
##  $ B4_AP_Abs_Mean    : num  0.328 0.304 0.26 0.5 0.296 ...
##  $ B4_ML_Abs_Mean    : num  0.201 0.267 0.162 0.273 0.252 ...
##  $ Aft_AP_Abs_Mean   : num  0.638 1.308 0.379 1.141 1.3 ...
##  $ Aft_ML_Abs_Mean   : num  0.438 0.498 0.213 2.467 0.941 ...
##  $ B4_AP_Max         : num  0.848 1.018 0.861 1.764 0.823 ...
##  $ B4_ML_Max         : num  0.69 0.964 0.672 1.053 0.948 ...
##  $ Aft_AP_Max        : num  2.43 6.21 1.32 2.93 3.43 ...
##  $ Aft_ML_Max        : num  1.257 2.495 0.983 4.36 4.102 ...
##  $ AP_Abs_Mean_All   : num  0.55 1.021 0.345 0.958 1.013 ...
##  $ ML_Abs_Mean_All   : num  0.37 0.432 0.198 1.84 0.744 ...
##  $ AP_Abs_Std_All    : num  0.55 1.021 0.345 0.958 1.013 ...
##  $ ML_Abs_Std_All    : num  0.37 0.432 0.198 1.84 0.744 ...
##  - attr(*, ".internal.selfref")=<externalptr>
```

## Tag as numbers and factors

```
ex$State_Number <- as.factor(ex$State_Number)
```

Double check that they are now factors (need to understand if they
are factors or numbers)

```
str(ex)
```

```
## Classes 'data.table' and 'data.frame':   120 obs. of  16 variables:
##  $ Participant_Number: chr  "PS1F03" "PS1F03" "PS1F03" "PS1F03" ...
##  $ State_Number      : Factor w/ 8 levels "Anterior-High",..: 2 1 6 5 8 7 4 3 2 1 ...
##  $ Ill_Dir           : chr  "Anterior" "Anterior" "Medial" "Medial" ...
##  $ Ill_Mag           : chr  "Low" "High" "Low" "High" ...
##  $ B4_AP_Abs_Mean    : num  0.328 0.304 0.26 0.5 0.296 ...
##  $ B4_ML_Abs_Mean    : num  0.201 0.267 0.162 0.273 0.252 ...
##  $ Aft_AP_Abs_Mean   : num  0.638 1.308 0.379 1.141 1.3 ...
##  $ Aft_ML_Abs_Mean   : num  0.438 0.498 0.213 2.467 0.941 ...
##  $ B4_AP_Max         : num  0.848 1.018 0.861 1.764 0.823 ...
##  $ B4_ML_Max         : num  0.69 0.964 0.672 1.053 0.948 ...
##  $ Aft_AP_Max        : num  2.43 6.21 1.32 2.93 3.43 ...
##  $ Aft_ML_Max        : num  1.257 2.495 0.983 4.36 4.102 ...
##  $ AP_Abs_Mean_All   : num  0.55 1.021 0.345 0.958 1.013 ...
##  $ ML_Abs_Mean_All   : num  0.37 0.432 0.198 1.84 0.744 ...
##  $ AP_Abs_Std_All    : num  0.55 1.021 0.345 0.958 1.013 ...
##  $ ML_Abs_Std_All    : num  0.37 0.432 0.198 1.84 0.744 ...
##  - attr(*, ".internal.selfref")=<externalptr>
```

```
long_df_Max_AP <-  pivot_longer(ex,
                         cols = c(B4_AP_Max, Aft_AP_Max),
                         names_to = "Time_Max_AP",
                         values_to = "Max_Disp_AP")
long_df_Max_AP$Time_Max_AP <- factor(long_df_Max_AP$Time_Max_AP, levels = c("B4_AP_Max", "Aft_AP_Max"))

long_df_Mean_AP <-  pivot_longer(ex,
                         cols = c(B4_AP_Abs_Mean, Aft_AP_Abs_Mean),
                         names_to = "Time_Mean_AP",
                         values_to = "Mean_Disp_AP")
long_df_Mean_AP$Time_Mean_AP <- factor(long_df_Mean_AP$Time_Mean_AP, levels = c("B4_AP_Abs_Mean", "Aft_AP_Abs_Mean"))

long_df_Max_ML <-  pivot_longer(ex,
                         cols = c(B4_ML_Max, Aft_ML_Max),
                         names_to = "Time_Max_ML",
                         values_to = "Max_Disp_ML")
long_df_Max_ML$Time_Max_ML <- factor(long_df_Max_ML$Time_Max_ML, levels = c("B4_ML_Max", "Aft_ML_Max"))

long_df_Mean_ML <-  pivot_longer(ex,
                         cols = c(B4_ML_Abs_Mean, Aft_ML_Abs_Mean),
                         names_to = "Time_Mean_ML",
                         values_to = "Mean_Disp_ML")
long_df_Mean_ML$Time_Mean_ML <- factor(long_df_Mean_ML$Time_Mean_ML, levels = c("B4_ML_Abs_Mean", "Aft_ML_Abs_Mean"))

str(long_df_Max_AP)
```

```
## tibble [240 × 16] (S3: tbl_df/tbl/data.frame)
##  $ Participant_Number: chr [1:240] "PS1F03" "PS1F03" "PS1F03" "PS1F03" ...
##  $ State_Number      : Factor w/ 8 levels "Anterior-High",..: 2 2 1 1 6 6 5 5 8 8 ...
##  $ Ill_Dir           : chr [1:240] "Anterior" "Anterior" "Anterior" "Anterior" ...
##  $ Ill_Mag           : chr [1:240] "Low" "Low" "High" "High" ...
##  $ B4_AP_Abs_Mean    : num [1:240] 0.328 0.328 0.304 0.304 0.26 ...
##  $ B4_ML_Abs_Mean    : num [1:240] 0.201 0.201 0.267 0.267 0.162 ...
##  $ Aft_AP_Abs_Mean   : num [1:240] 0.638 0.638 1.308 1.308 0.379 ...
##  $ Aft_ML_Abs_Mean   : num [1:240] 0.438 0.438 0.498 0.498 0.213 ...
##  $ B4_ML_Max         : num [1:240] 0.69 0.69 0.964 0.964 0.672 ...
##  $ Aft_ML_Max        : num [1:240] 1.257 1.257 2.495 2.495 0.983 ...
##  $ AP_Abs_Mean_All   : num [1:240] 0.55 0.55 1.021 1.021 0.345 ...
##  $ ML_Abs_Mean_All   : num [1:240] 0.37 0.37 0.432 0.432 0.198 ...
##  $ AP_Abs_Std_All    : num [1:240] 0.55 0.55 1.021 1.021 0.345 ...
##  $ ML_Abs_Std_All    : num [1:240] 0.37 0.37 0.432 0.432 0.198 ...
##  $ Time_Max_AP       : Factor w/ 2 levels "B4_AP_Max","Aft_AP_Max": 1 2 1 2 1 2 1 2 1 2 ...
##  $ Max_Disp_AP       : num [1:240] 0.848 2.427 1.018 6.212 0.861 ...
```

```
str(long_df_Max_ML)
```

```
## tibble [240 × 16] (S3: tbl_df/tbl/data.frame)
##  $ Participant_Number: chr [1:240] "PS1F03" "PS1F03" "PS1F03" "PS1F03" ...
##  $ State_Number      : Factor w/ 8 levels "Anterior-High",..: 2 2 1 1 6 6 5 5 8 8 ...
##  $ Ill_Dir           : chr [1:240] "Anterior" "Anterior" "Anterior" "Anterior" ...
##  $ Ill_Mag           : chr [1:240] "Low" "Low" "High" "High" ...
##  $ B4_AP_Abs_Mean    : num [1:240] 0.328 0.328 0.304 0.304 0.26 ...
##  $ B4_ML_Abs_Mean    : num [1:240] 0.201 0.201 0.267 0.267 0.162 ...
##  $ Aft_AP_Abs_Mean   : num [1:240] 0.638 0.638 1.308 1.308 0.379 ...
##  $ Aft_ML_Abs_Mean   : num [1:240] 0.438 0.438 0.498 0.498 0.213 ...
##  $ B4_AP_Max         : num [1:240] 0.848 0.848 1.018 1.018 0.861 ...
##  $ Aft_AP_Max        : num [1:240] 2.43 2.43 6.21 6.21 1.32 ...
##  $ AP_Abs_Mean_All   : num [1:240] 0.55 0.55 1.021 1.021 0.345 ...
##  $ ML_Abs_Mean_All   : num [1:240] 0.37 0.37 0.432 0.432 0.198 ...
##  $ AP_Abs_Std_All    : num [1:240] 0.55 0.55 1.021 1.021 0.345 ...
##  $ ML_Abs_Std_All    : num [1:240] 0.37 0.37 0.432 0.432 0.198 ...
##  $ Time_Max_ML       : Factor w/ 2 levels "B4_ML_Max","Aft_ML_Max": 1 2 1 2 1 2 1 2 1 2 ...
##  $ Max_Disp_ML       : num [1:240] 0.69 1.257 0.964 2.495 0.672 ...
```

```
str(long_df_Mean_AP)
```

```
## tibble [240 × 16] (S3: tbl_df/tbl/data.frame)
##  $ Participant_Number: chr [1:240] "PS1F03" "PS1F03" "PS1F03" "PS1F03" ...
##  $ State_Number      : Factor w/ 8 levels "Anterior-High",..: 2 2 1 1 6 6 5 5 8 8 ...
##  $ Ill_Dir           : chr [1:240] "Anterior" "Anterior" "Anterior" "Anterior" ...
##  $ Ill_Mag           : chr [1:240] "Low" "Low" "High" "High" ...
##  $ B4_ML_Abs_Mean    : num [1:240] 0.201 0.201 0.267 0.267 0.162 ...
##  $ Aft_ML_Abs_Mean   : num [1:240] 0.438 0.438 0.498 0.498 0.213 ...
##  $ B4_AP_Max         : num [1:240] 0.848 0.848 1.018 1.018 0.861 ...
##  $ B4_ML_Max         : num [1:240] 0.69 0.69 0.964 0.964 0.672 ...
##  $ Aft_AP_Max        : num [1:240] 2.43 2.43 6.21 6.21 1.32 ...
##  $ Aft_ML_Max        : num [1:240] 1.257 1.257 2.495 2.495 0.983 ...
##  $ AP_Abs_Mean_All   : num [1:240] 0.55 0.55 1.021 1.021 0.345 ...
##  $ ML_Abs_Mean_All   : num [1:240] 0.37 0.37 0.432 0.432 0.198 ...
##  $ AP_Abs_Std_All    : num [1:240] 0.55 0.55 1.021 1.021 0.345 ...
##  $ ML_Abs_Std_All    : num [1:240] 0.37 0.37 0.432 0.432 0.198 ...
##  $ Time_Mean_AP      : Factor w/ 2 levels "B4_AP_Abs_Mean",..: 1 2 1 2 1 2 1 2 1 2 ...
##  $ Mean_Disp_AP      : num [1:240] 0.328 0.638 0.304 1.308 0.26 ...
```

```
str(long_df_Mean_ML)
```

```
## tibble [240 × 16] (S3: tbl_df/tbl/data.frame)
##  $ Participant_Number: chr [1:240] "PS1F03" "PS1F03" "PS1F03" "PS1F03" ...
##  $ State_Number      : Factor w/ 8 levels "Anterior-High",..: 2 2 1 1 6 6 5 5 8 8 ...
##  $ Ill_Dir           : chr [1:240] "Anterior" "Anterior" "Anterior" "Anterior" ...
##  $ Ill_Mag           : chr [1:240] "Low" "Low" "High" "High" ...
##  $ B4_AP_Abs_Mean    : num [1:240] 0.328 0.328 0.304 0.304 0.26 ...
##  $ Aft_AP_Abs_Mean   : num [1:240] 0.638 0.638 1.308 1.308 0.379 ...
##  $ B4_AP_Max         : num [1:240] 0.848 0.848 1.018 1.018 0.861 ...
##  $ B4_ML_Max         : num [1:240] 0.69 0.69 0.964 0.964 0.672 ...
##  $ Aft_AP_Max        : num [1:240] 2.43 2.43 6.21 6.21 1.32 ...
##  $ Aft_ML_Max        : num [1:240] 1.257 1.257 2.495 2.495 0.983 ...
##  $ AP_Abs_Mean_All   : num [1:240] 0.55 0.55 1.021 1.021 0.345 ...
##  $ ML_Abs_Mean_All   : num [1:240] 0.37 0.37 0.432 0.432 0.198 ...
##  $ AP_Abs_Std_All    : num [1:240] 0.55 0.55 1.021 1.021 0.345 ...
##  $ ML_Abs_Std_All    : num [1:240] 0.37 0.37 0.432 0.432 0.198 ...
##  $ Time_Mean_ML      : Factor w/ 2 levels "B4_ML_Abs_Mean",..: 1 2 1 2 1 2 1 2 1 2 ...
##  $ Mean_Disp_ML      : num [1:240] 0.201 0.438 0.267 0.498 0.162 ...
```

## Mean ML

```
# Create the boxplot
boxplot(Mean_Disp_ML ~ Time_Mean_ML, 
        data = long_df_Mean_ML, 
        col = c("lightblue", "pink"),     # Set two distinct colors
        ylab = " ", 
        xlab = " ")
```

```
# t test
t.test(long_df_Mean_ML$Mean_Disp_ML[long_df_Mean_ML$Time_Mean_ML == "Aft_ML_Abs_Mean"],
       long_df_Mean_ML$Mean_Disp_ML[long_df_Mean_ML$Time_Mean_ML == "B4_ML_Abs_Mean"],
       paired = TRUE)
```

```
## 
##  Paired t-test
## 
## data:  long_df_Mean_ML$Mean_Disp_ML[long_df_Mean_ML$Time_Mean_ML == "Aft_ML_Abs_Mean"] and long_df_Mean_ML$Mean_Disp_ML[long_df_Mean_ML$Time_Mean_ML == "B4_ML_Abs_Mean"]
## t = 6.1296, df = 119, p-value = 1.18e-08
## alternative hypothesis: true mean difference is not equal to 0
## 95 percent confidence interval:
##  1.096038 2.142062
## sample estimates:
## mean difference 
##         1.61905
```

```
pd = position_dodge(width = 0.5)

#Reference Plot
p <- ggplot(long_df_Mean_ML, aes(x = State_Number, y = Mean_Disp_ML, fill= Time_Mean_ML))
p <- p + stat_boxplot(geom = "errorbar", position=pd, width = 0.2)
p <- p + geom_boxplot(width=0.5, position=pd)
p <- p + labs(title = "Absolute Mean CoP Displacement in ML Direction Before and After Illusions ",
              y = "Absolute CoP Displacement (cm)",
              x = "Illusion Type")
p <- p + scale_fill_manual(name = "State", 
                    values = c("B4_ML_Abs_Mean" = "lightblue", "Aft_ML_Abs_Mean" = "pink"), 
                    labels = c("B4_ML_Abs_Mean" = "Before Illusion", "Aft_ML_Abs_Mean" = "After Illusion"))
p <- p + theme(panel.background = element_rect(fill = "white", color = NA),  # White background
               axis.line = element_line(color = "black"),  # Black axes lines
               axis.ticks.length = unit(0.2, "cm"),
               axis.ticks = element_line(color = "black"),
               panel.border = element_rect(color = "black", fill = NA, size = 1),  # Add border around the plot
               legend.position = c(0.8, 0.9),  # Position legend at the bottom
               axis.title.x = element_text(size = 16),  # Increase x-axis title font size
               axis.title.y = element_text(size = 16),  # Increase y-axis title font size
               axis.text.x = element_text(size = 14),   # Increase x-axis labels font size
               axis.text.y = element_text(size = 14),   # Increase y-axis labels font size
               legend.text = element_text(size = 14),   # Increase legend text font size
               legend.title = element_blank()           # Remove legend title
               )
```

```
## Warning: The `size` argument of `element_rect()` is deprecated as of ggplot2 3.4.0.
## ℹ Please use the `linewidth` argument instead.
## This warning is displayed once every 8 hours.
## Call `lifecycle::last_lifecycle_warnings()` to see where this warning was
## generated.
```

```
## Warning: A numeric `legend.position` argument in `theme()` was deprecated in ggplot2
## 3.5.0.
## ℹ Please use the `legend.position.inside` argument of `theme()` instead.
## This warning is displayed once every 8 hours.
## Call `lifecycle::last_lifecycle_warnings()` to see where this warning was
## generated.
```

```
print(p)
```

```
# Summary table for Mean ML Displacement
summary_table_mean_ML <- long_df_Mean_ML %>%
  group_by(State_Number, Time_Mean_ML) %>%
  summarise(
    Minimum_Error = min(Mean_Disp_ML),
    Q1 = quantile(Mean_Disp_ML, 0.25),
    Median = median(Mean_Disp_ML),
    Q3 = quantile(Mean_Disp_ML, 0.75),
    Maximum_Error = max(Mean_Disp_ML),
    Mean = mean(Mean_Disp_ML),
    SD = sd(Mean_Disp_ML)
  )
```

```
## `summarise()` has grouped output by 'State_Number'. You can override using the
## `.groups` argument.
```

```
print(summary_table_mean_ML)
```

```
## # A tibble: 16 × 9
## # Groups:   State_Number [8]
##    State_Number   Time_Mean_ML    Minimum_Error    Q1 Median    Q3 Maximum_Error
##    <fct>          <fct>                   <dbl> <dbl>  <dbl> <dbl>         <dbl>
##  1 Anterior-High  B4_ML_Abs_Mean         0.180  0.269  0.359 0.553          2.64
##  2 Anterior-High  Aft_ML_Abs_Mean        0.498  1.74   4.44  6.00          16.7 
##  3 Anterior-Low   B4_ML_Abs_Mean         0.177  0.218  0.326 0.586          3.38
##  4 Anterior-Low   Aft_ML_Abs_Mean        0.118  0.347  1.03  1.83          12.6 
##  5 Lateral-High   B4_ML_Abs_Mean         0.172  0.205  0.447 1.39           2.33
##  6 Lateral-High   Aft_ML_Abs_Mean        0.333  0.544  1.11  2.75          11.4 
##  7 Lateral-Low    B4_ML_Abs_Mean         0.141  0.174  0.237 0.631          3.27
##  8 Lateral-Low    Aft_ML_Abs_Mean        0.182  0.279  0.451 1.08           3.41
##  9 Medial-High    B4_ML_Abs_Mean         0.0994 0.270  0.361 0.851          3.95
## 10 Medial-High    Aft_ML_Abs_Mean        0.251  1.16   2.39  3.54          14.0 
## 11 Medial-Low     B4_ML_Abs_Mean         0.134  0.199  0.352 0.910          4.54
## 12 Medial-Low     Aft_ML_Abs_Mean        0.172  0.544  0.836 2.11           4.46
## 13 Posterior-High B4_ML_Abs_Mean         0.144  0.272  0.381 0.906          3.98
## 14 Posterior-High Aft_ML_Abs_Mean        0.766  1.30   1.92  3.69           9.28
## 15 Posterior-Low  B4_ML_Abs_Mean         0.0857 0.160  0.252 0.444          5.80
## 16 Posterior-Low  Aft_ML_Abs_Mean        0.183  0.579  1.01  2.39           6.15
## # ℹ 2 more variables: Mean <dbl>, SD <dbl>
```

## Mean AP

```
# Create the boxplot
boxplot(Mean_Disp_AP ~ Time_Mean_AP, 
        data = long_df_Mean_AP, 
        col = c("orange", "lightgreen"),     # Set two distinct colors
        ylab = " ", 
        xlab = " ")
```

```
# t test
t.test(long_df_Mean_AP$Mean_Disp_AP[long_df_Mean_AP$Time_Mean_AP == "Aft_AP_Abs_Mean"],
       long_df_Mean_AP$Mean_Disp_AP[long_df_Mean_AP$Time_Mean_AP == "B4_AP_Abs_Mean"],
       paired = TRUE)
```

```
## 
##  Paired t-test
## 
## data:  long_df_Mean_AP$Mean_Disp_AP[long_df_Mean_AP$Time_Mean_AP == "Aft_AP_Abs_Mean"] and long_df_Mean_AP$Mean_Disp_AP[long_df_Mean_AP$Time_Mean_AP == "B4_AP_Abs_Mean"]
## t = 8.2044, df = 119, p-value = 3.118e-13
## alternative hypothesis: true mean difference is not equal to 0
## 95 percent confidence interval:
##  0.3866170 0.6326013
## sample estimates:
## mean difference 
##       0.5096092
```

```
p <- ggplot(long_df_Mean_AP, aes(x = State_Number, y = Mean_Disp_AP, fill= Time_Mean_AP))
p <- p + stat_boxplot(geom = "errorbar", position=pd, width = 0.2)
p <- p + geom_boxplot(width=0.5, position=pd)
p <- p + labs(title = "Absolute Mean CoP Displacement in AP Direction Before and After Illusions ",
              y = "Absolute CoP Displacement (cm)",
              x = "Illusion Type")
p <- p + scale_fill_manual(name = "State", 
                    values = c("B4_AP_Abs_Mean" = "orange", "Aft_AP_Abs_Mean" = "lightgreen"), 
                    labels = c("B4_AP_Abs_Mean" = "Before Illusion", "Aft_AP_Abs_Mean" = "After Illusion"))
p <- p + theme(panel.background = element_rect(fill = "white", color = NA),  # White background
               axis.line = element_line(color = "black"),  # Black axes lines
               axis.ticks.length = unit(0.2, "cm"),
               axis.ticks = element_line(color = "black"),
               panel.border = element_rect(color = "black", fill = NA, size = 1),  # Add border around the plot
               legend.position = c(0.8, 0.9) , # Position legend at the bottom,
               axis.title.x = element_text(size = 16),  # Increase x-axis title font size
               axis.title.y = element_text(size = 16),  # Increase y-axis title font size
               axis.text.x = element_text(size = 14),   # Increase x-axis labels font size
               axis.text.y = element_text(size = 14),   # Increase y-axis labels font size
               legend.text = element_text(size = 14),   # Increase legend text font size
               legend.title = element_blank()           # Remove legend title
               )

print(p)
```

```
# Summary table for Mean AP Displacement
summary_table_mean_AP <- long_df_Mean_AP %>%
  group_by(State_Number, Time_Mean_AP) %>%
  summarise(
    Minimum_Error = min(Mean_Disp_AP),
    Q1 = quantile(Mean_Disp_AP, 0.25),
    Median = median(Mean_Disp_AP),
    Q3 = quantile(Mean_Disp_AP, 0.75),
    Maximum_Error = max(Mean_Disp_AP),
    Mean = mean(Mean_Disp_AP),
    SD = sd(Mean_Disp_AP)
  )
```

```
## `summarise()` has grouped output by 'State_Number'. You can override using the
## `.groups` argument.
```

```
print(summary_table_mean_AP)
```

```
## # A tibble: 16 × 9
## # Groups:   State_Number [8]
##    State_Number   Time_Mean_AP    Minimum_Error    Q1 Median    Q3 Maximum_Error
##    <fct>          <fct>                   <dbl> <dbl>  <dbl> <dbl>         <dbl>
##  1 Anterior-High  B4_AP_Abs_Mean          0.285 0.379  0.446 0.632          1.30
##  2 Anterior-High  Aft_AP_Abs_Mean         0.685 0.978  1.50  2.46           2.81
##  3 Anterior-Low   B4_AP_Abs_Mean          0.295 0.353  0.419 0.569          1.33
##  4 Anterior-Low   Aft_AP_Abs_Mean         0.249 0.466  1.00  1.33           4.18
##  5 Lateral-High   B4_AP_Abs_Mean          0.313 0.375  0.461 0.588          1.23
##  6 Lateral-High   Aft_AP_Abs_Mean         0.235 0.682  0.810 1.19           1.95
##  7 Lateral-Low    B4_AP_Abs_Mean          0.229 0.359  0.459 0.571          1.54
##  8 Lateral-Low    Aft_AP_Abs_Mean         0.282 0.436  0.555 0.862          1.41
##  9 Medial-High    B4_AP_Abs_Mean          0.299 0.410  0.627 0.797          1.90
## 10 Medial-High    Aft_AP_Abs_Mean         0.476 0.550  0.702 0.949          1.47
## 11 Medial-Low     B4_AP_Abs_Mean          0.248 0.351  0.413 0.548          1.29
## 12 Medial-Low     Aft_AP_Abs_Mean         0.217 0.439  0.621 0.768          1.37
## 13 Posterior-High B4_AP_Abs_Mean          0.232 0.325  0.492 0.678          1.85
## 14 Posterior-High Aft_AP_Abs_Mean         0.803 1.03   1.40  2.36           3.38
## 15 Posterior-Low  B4_AP_Abs_Mean          0.177 0.344  0.454 0.729          1.18
## 16 Posterior-Low  Aft_AP_Abs_Mean         0.357 0.632  0.844 1.09           2.82
## # ℹ 2 more variables: Mean <dbl>, SD <dbl>
```

## Max ML

```
# Create the boxplot
boxplot(Max_Disp_ML ~ Time_Max_ML, 
        data = long_df_Max_ML, 
        col = c("lightblue", "pink"),     # Set two distinct colors
        ylab = " ", 
        xlab = " ")
```

```
# t test
t.test(long_df_Max_ML$Max_Disp_ML[long_df_Max_ML$Time_Max_ML == "Aft_ML_Max"],
       long_df_Max_ML$Max_Disp_ML[long_df_Max_ML$Time_Max_ML == "B4_ML_Max"],
       paired = TRUE)
```

```
## 
##  Paired t-test
## 
## data:  long_df_Max_ML$Max_Disp_ML[long_df_Max_ML$Time_Max_ML == "Aft_ML_Max"] and long_df_Max_ML$Max_Disp_ML[long_df_Max_ML$Time_Max_ML == "B4_ML_Max"]
## t = 6.8458, df = 119, p-value = 3.537e-10
## alternative hypothesis: true mean difference is not equal to 0
## 95 percent confidence interval:
##  2.869383 5.204781
## sample estimates:
## mean difference 
##        4.037082
```

```
p <- ggplot(long_df_Max_ML, aes(x = State_Number, y = Max_Disp_ML, fill= Time_Max_ML))
p <- p + stat_boxplot(geom = "errorbar", position=pd, width = 0.2)
p <- p + geom_boxplot(width=0.5, position=pd)
p <- p + labs(title = "Absolute Maximum CoP Displacement in ML Direction Before and After Illusions ",
              y = "Absolute CoP Displacement (cm)",
              x = "Illusion Type")
p <- p + scale_fill_manual(name = "State", 
                    values = c("B4_ML_Max" = "lightblue", "Aft_ML_Max" = "pink"), 
                    labels = c("B4_ML_Max" = "Before Illusion", "Aft_ML_Max" = "After Illusion"))
p <- p + theme(panel.background = element_rect(fill = "white", color = NA),  # White background
               axis.line = element_line(color = "black"),  # Black axes lines
               axis.ticks.length = unit(0.2, "cm"),
               axis.ticks = element_line(color = "black"),
               panel.border = element_rect(color = "black", fill = NA, size = 1),  # Add border around the plot
               legend.position = c(0.8, 0.9),  # Position legend at the bottom
               axis.title.x = element_text(size = 16),  # Increase x-axis title font size
               axis.title.y = element_text(size = 16),  # Increase y-axis title font size
               axis.text.x = element_text(size = 14),   # Increase x-axis labels font size
               axis.text.y = element_text(size = 14),   # Increase y-axis labels font size
               legend.text = element_text(size = 14),   # Increase legend text font size
               legend.title = element_blank()           # Remove legend title
               )
print(p)
```

```
# Summary table for Max ML Displacement
summary_table_max_ML <- long_df_Max_ML %>%
  group_by(State_Number, Time_Max_ML) %>%
  summarise(
    Minimum_Error = min(Max_Disp_ML),
    Q1 = quantile(Max_Disp_ML, 0.25),
    Median = median(Max_Disp_ML),
    Q3 = quantile(Max_Disp_ML, 0.75),
    Maximum_Error = max(Max_Disp_ML),
    Mean = mean(Max_Disp_ML),
    SD = sd(Max_Disp_ML)
  )
```

```
## `summarise()` has grouped output by 'State_Number'. You can override using the
## `.groups` argument.
```

```
print(summary_table_max_ML)
```

```
## # A tibble: 16 × 9
## # Groups:   State_Number [8]
##    State_Number Time_Max_ML Minimum_Error    Q1 Median    Q3 Maximum_Error  Mean
##    <fct>        <fct>               <dbl> <dbl>  <dbl> <dbl>         <dbl> <dbl>
##  1 Anterior-Hi… B4_ML_Max           0.889 1.08   1.96   4.67          23.6  4.13
##  2 Anterior-Hi… Aft_ML_Max          2.50  7.48  11.2   18.9           46.0 14.8 
##  3 Anterior-Low B4_ML_Max           0.531 0.954  1.34   3.42          20.8  4.40
##  4 Anterior-Low Aft_ML_Max          0.484 1.42   2.54   9.41          32.0  7.06
##  5 Lateral-High B4_ML_Max           0.655 0.779  1.90   6.77          20.0  4.83
##  6 Lateral-High Aft_ML_Max          1.32  2.04   6.67  12.9           33.1  8.66
##  7 Lateral-Low  B4_ML_Max           0.482 0.655  0.859  5.15          31.1  4.88
##  8 Lateral-Low  Aft_ML_Max          0.906 1.23   2.43   5.07          27.9  5.65
##  9 Medial-High  B4_ML_Max           0.462 1.05   1.79   9.04          25.0  5.52
## 10 Medial-High  Aft_ML_Max          1.39  4.42   5.96  10.5           16.7  7.83
## 11 Medial-Low   B4_ML_Max           0.408 0.736  2.05   5.15          33.3  5.23
## 12 Medial-Low   Aft_ML_Max          0.850 1.74   3.94  11.6           32.1  7.69
## 13 Posterior-H… B4_ML_Max           0.549 0.966  1.22   7.52          13.5  4.50
## 14 Posterior-H… Aft_ML_Max          2.66  5.48   8.78  15.0           28.2 10.6 
## 15 Posterior-L… B4_ML_Max           0.351 0.548  1.25   2.15          31.9  4.55
## 16 Posterior-L… Aft_ML_Max          0.813 1.52   4.31   9.03          42.1  8.05
## # ℹ 1 more variable: SD <dbl>
```

## Max AP

```
# Create the boxplot
boxplot(Max_Disp_AP ~ Time_Max_AP, 
        data = long_df_Max_AP, 
        col = c("orange", "lightgreen"),     # Set two distinct colors
        ylab = " ", 
        xlab = " ")
```

```
# t test
t.test(long_df_Max_AP$Max_Disp_AP[long_df_Max_AP$Time_Max_AP == "Aft_AP_Max"],
       long_df_Max_AP$Max_Disp_AP[long_df_Max_AP$Time_Max_AP == "B4_AP_Max"],
       paired = TRUE)
```

```
## 
##  Paired t-test
## 
## data:  long_df_Max_AP$Max_Disp_AP[long_df_Max_AP$Time_Max_AP == "Aft_AP_Max"] and long_df_Max_AP$Max_Disp_AP[long_df_Max_AP$Time_Max_AP == "B4_AP_Max"]
## t = 9.9573, df = 119, p-value < 2.2e-16
## alternative hypothesis: true mean difference is not equal to 0
## 95 percent confidence interval:
##  1.500584 2.245533
## sample estimates:
## mean difference 
##        1.873058
```

```
p <- ggplot(long_df_Max_AP, aes(x = State_Number, y = Max_Disp_AP, fill= Time_Max_AP))
p <- p + stat_boxplot(geom = "errorbar", position=pd, width = 0.2)
p <- p + geom_boxplot(width=0.5, position=pd)
p <- p + labs(title = "Absolute Maximum CoP Displacement in AP Direction Before and After Illusions ",
              y = "Absolute CoP Displacement (cm)",
              x = "Illusion Type")
p <- p + scale_fill_manual(name = "State", 
                    values = c("B4_AP_Max" = "orange", "Aft_AP_Max" = "lightgreen"), 
                    labels = c("B4_AP_Max" = "Before Illusion", "Aft_AP_Max" = "After Illusion"))
p <- p + theme(panel.background = element_rect(fill = "white", color = NA),  # White background
               axis.line = element_line(color = "black"),  # Black axes lines
               axis.ticks.length = unit(0.2, "cm"),
               axis.ticks = element_line(color = "black"),
               panel.border = element_rect(color = "black", fill = NA, size = 1),  # Add border around the plot
               legend.position = c(0.6, 0.9),  # Position legend at the bottom
               axis.title.x = element_text(size = 16),  # Increase x-axis title font size
    axis.title.y = element_text(size = 16),  # Increase y-axis title font size
    axis.text.x = element_text(size = 14),   # Increase x-axis labels font size
    axis.text.y = element_text(size = 14),   # Increase y-axis labels font size
    legend.text = element_text(size = 14),   # Increase legend text font size
    legend.title = element_blank()           # Remove legend title
               )
print(p)
```

```
# Summary table for Max AP Displacement
summary_table_max_AP <- long_df_Max_AP %>%
  group_by(State_Number, Time_Max_AP) %>%
  summarise(
    Minimum_Error = min(Max_Disp_AP),
    Q1 = quantile(Max_Disp_AP, 0.25),
    Median = median(Max_Disp_AP),
    Q3 = quantile(Max_Disp_AP, 0.75),
    Maximum_Error = max(Max_Disp_AP),
    Mean = mean(Max_Disp_AP),
    SD = sd(Max_Disp_AP)
  )
```

```
## `summarise()` has grouped output by 'State_Number'. You can override using the
## `.groups` argument.
```

```
print(summary_table_max_AP)
```

```
## # A tibble: 16 × 9
## # Groups:   State_Number [8]
##    State_Number Time_Max_AP Minimum_Error    Q1 Median    Q3 Maximum_Error  Mean
##    <fct>        <fct>               <dbl> <dbl>  <dbl> <dbl>         <dbl> <dbl>
##  1 Anterior-Hi… B4_AP_Max           0.925  1.14   1.68  1.96          6.66  1.93
##  2 Anterior-Hi… Aft_AP_Max          2.04   3.90   5.53  7.13         10.7   5.76
##  3 Anterior-Low B4_AP_Max           0.848  1.35   1.53  2.24          5.74  1.97
##  4 Anterior-Low Aft_AP_Max          0.948  2.24   2.43  5.65          9.50  3.97
##  5 Lateral-High B4_AP_Max           1.12   1.40   1.74  2.31          5.45  2.10
##  6 Lateral-High Aft_AP_Max          1.16   2.72   3.26  4.31          8.46  3.69
##  7 Lateral-Low  B4_AP_Max           0.861  1.07   1.42  2.26          7.15  2.01
##  8 Lateral-Low  Aft_AP_Max          1.12   1.45   1.87  2.67          7.58  2.47
##  9 Medial-High  B4_AP_Max           0.731  1.45   2.46  3.60          6.51  2.63
## 10 Medial-High  Aft_AP_Max          1.60   2.13   2.93  4.03          6.99  3.38
## 11 Medial-Low   B4_AP_Max           0.703  1.14   1.32  1.83          8.87  2.17
## 12 Medial-Low   Aft_AP_Max          1.08   1.48   2.04  2.90          8.64  2.75
## 13 Posterior-H… B4_AP_Max           0.862  1.05   1.62  2.63          5.83  2.02
## 14 Posterior-H… Aft_AP_Max          2.93   4.29   6.25  8.16         10.6   6.22
## 15 Posterior-L… B4_AP_Max           0.605  1.21   1.56  2.76          6.68  2.23
## 16 Posterior-L… Aft_AP_Max          1.09   3.04   3.45  4.20          9.73  3.83
## # ℹ 1 more variable: SD <dbl>
```

```
# Initialize a data frame to store the results
t_mean_summary_table_ML <- data.frame(State_Number = character(),
                           mean_P_Value_ML = numeric(),
                           mean_Diff_ML = numeric(),
                            stringsAsFactors = FALSE)

t_mean_summary_table_AP <- data.frame(State_Number = character(),
                            mean_P_Value_AP = numeric(),
                            mean_Diff_AP = numeric(),
                            stringsAsFactors = FALSE)

t_max_summary_table_ML <- data.frame(State_Number = character(),
                            max_P_Value_ML = numeric(),
                            max_Diff_ML = numeric(),
                            stringsAsFactors = FALSE)

t_max_summary_table_AP <- data.frame(State_Number = character(),
                            max_P_Value_AP = numeric(),
                            max_Diff_AP = numeric(),
                            stringsAsFactors = FALSE)

# List to store the results
t_test_mean_results_ML <- list()
t_test_mean_results_AP <- list()
t_test_max_results_ML <- list()
t_test_max_results_AP <- list()

##### Mean ML
# Loop through each State_Number
for (state in unique(long_df_Mean_ML$State_Number)) {
  # Subset the data for the current state
  mean_state_data_ML <- subset(long_df_Mean_ML, State_Number == state)
  
  # Perform the paired t-test
  mean_t_test_ML <- t.test(mean_state_data_ML$Mean_Disp_ML[mean_state_data_ML$Time_Mean_ML == "Aft_ML_Abs_Mean"],
                   mean_state_data_ML$Mean_Disp_ML[mean_state_data_ML$Time_Mean_ML == "B4_ML_Abs_Mean"],
                   paired = TRUE)
  
  # Store the result in the list with the state as the name
  t_test_mean_results_ML[[state]] <- mean_t_test_ML
  
  # Print the result
  print(paste("State:", state))
  print(mean_t_test_ML)
  
  # Add the result to the summary table
  t_mean_summary_table_ML <- rbind(t_mean_summary_table_ML, data.frame(State_Number = state,
                                                   mean_P_Value_ML = mean_t_test_ML$p.value,
                                                   mean_Diff_ML = mean_t_test_ML$estimate))
}
```

```
## [1] "State: Anterior-Low"
## 
##  Paired t-test
## 
## data:  mean_state_data_ML$Mean_Disp_ML[mean_state_data_ML$Time_Mean_ML == "Aft_ML_Abs_Mean"] and mean_state_data_ML$Mean_Disp_ML[mean_state_data_ML$Time_Mean_ML == "B4_ML_Abs_Mean"]
## t = 1.7986, df = 14, p-value = 0.09366
## alternative hypothesis: true mean difference is not equal to 0
## 95 percent confidence interval:
##  -0.2406803  2.7419205
## sample estimates:
## mean difference 
##         1.25062 
## 
## [1] "State: Anterior-High"
## 
##  Paired t-test
## 
## data:  mean_state_data_ML$Mean_Disp_ML[mean_state_data_ML$Time_Mean_ML == "Aft_ML_Abs_Mean"] and mean_state_data_ML$Mean_Disp_ML[mean_state_data_ML$Time_Mean_ML == "B4_ML_Abs_Mean"]
## t = 3.845, df = 14, p-value = 0.001785
## alternative hypothesis: true mean difference is not equal to 0
## 95 percent confidence interval:
##  1.945249 6.853076
## sample estimates:
## mean difference 
##        4.399162 
## 
## [1] "State: Medial-Low"
## 
##  Paired t-test
## 
## data:  mean_state_data_ML$Mean_Disp_ML[mean_state_data_ML$Time_Mean_ML == "Aft_ML_Abs_Mean"] and mean_state_data_ML$Mean_Disp_ML[mean_state_data_ML$Time_Mean_ML == "B4_ML_Abs_Mean"]
## t = 2.1959, df = 14, p-value = 0.04545
## alternative hypothesis: true mean difference is not equal to 0
## 95 percent confidence interval:
##  0.00975277 0.82895851
## sample estimates:
## mean difference 
##       0.4193556 
## 
## [1] "State: Medial-High"
## 
##  Paired t-test
## 
## data:  mean_state_data_ML$Mean_Disp_ML[mean_state_data_ML$Time_Mean_ML == "Aft_ML_Abs_Mean"] and mean_state_data_ML$Mean_Disp_ML[mean_state_data_ML$Time_Mean_ML == "B4_ML_Abs_Mean"]
## t = 2.514, df = 14, p-value = 0.02479
## alternative hypothesis: true mean difference is not equal to 0
## 95 percent confidence interval:
##  0.3155599 3.9814414
## sample estimates:
## mean difference 
##        2.148501 
## 
## [1] "State: Posterior-Low"
## 
##  Paired t-test
## 
## data:  mean_state_data_ML$Mean_Disp_ML[mean_state_data_ML$Time_Mean_ML == "Aft_ML_Abs_Mean"] and mean_state_data_ML$Mean_Disp_ML[mean_state_data_ML$Time_Mean_ML == "B4_ML_Abs_Mean"]
## t = 3.5665, df = 14, p-value = 0.003098
## alternative hypothesis: true mean difference is not equal to 0
## 95 percent confidence interval:
##  0.3261309 1.3101423
## sample estimates:
## mean difference 
##       0.8181366 
## 
## [1] "State: Posterior-High"
## 
##  Paired t-test
## 
## data:  mean_state_data_ML$Mean_Disp_ML[mean_state_data_ML$Time_Mean_ML == "Aft_ML_Abs_Mean"] and mean_state_data_ML$Mean_Disp_ML[mean_state_data_ML$Time_Mean_ML == "B4_ML_Abs_Mean"]
## t = 2.7828, df = 14, p-value = 0.01467
## alternative hypothesis: true mean difference is not equal to 0
## 95 percent confidence interval:
##  0.4510418 3.4837384
## sample estimates:
## mean difference 
##         1.96739 
## 
## [1] "State: Lateral-Low"
## 
##  Paired t-test
## 
## data:  mean_state_data_ML$Mean_Disp_ML[mean_state_data_ML$Time_Mean_ML == "Aft_ML_Abs_Mean"] and mean_state_data_ML$Mean_Disp_ML[mean_state_data_ML$Time_Mean_ML == "B4_ML_Abs_Mean"]
## t = 0.77635, df = 14, p-value = 0.4505
## alternative hypothesis: true mean difference is not equal to 0
## 95 percent confidence interval:
##  -0.2311579  0.4934406
## sample estimates:
## mean difference 
##       0.1311413 
## 
## [1] "State: Lateral-High"
## 
##  Paired t-test
## 
## data:  mean_state_data_ML$Mean_Disp_ML[mean_state_data_ML$Time_Mean_ML == "Aft_ML_Abs_Mean"] and mean_state_data_ML$Mean_Disp_ML[mean_state_data_ML$Time_Mean_ML == "B4_ML_Abs_Mean"]
## t = 2.1672, df = 14, p-value = 0.04796
## alternative hypothesis: true mean difference is not equal to 0
## 95 percent confidence interval:
##  0.01877302 3.61741287
## sample estimates:
## mean difference 
##        1.818093
```

```
##### Mean AP
# Loop through each State_Number
for (state in unique(long_df_Mean_AP$State_Number)) {
  # Subset the data for the current state
  mean_state_data_AP <- subset(long_df_Mean_AP, State_Number == state)
  
  # Perform the paired t-test
  mean_t_test_AP <- t.test(mean_state_data_AP$Mean_Disp_AP[mean_state_data_AP$Time_Mean_AP == "Aft_AP_Abs_Mean"],
                   mean_state_data_AP$Mean_Disp_AP[mean_state_data_AP$Time_Mean_AP == "B4_AP_Abs_Mean"],
                   paired = TRUE)
  
  # Store the result in the list with the state as the name
  t_test_mean_results_AP[[state]] <- mean_t_test_AP
  
  # Print the result
 # print(paste("State:", state))
 # print(mean_t_test_AP)
  
  # Add the result to the summary table
  t_mean_summary_table_AP <- rbind(t_mean_summary_table_AP, data.frame(State_Number = state,
                                                   mean_P_Value_AP = mean_t_test_AP$p.value,
                                                   mean_Diff_AP = mean_t_test_AP$estimate))
}

##### Max ML
# Loop through each State_Number
for (state in unique(long_df_Max_ML$State_Number)) {
  # Subset the data for the current state
  max_state_data_ML <- subset(long_df_Max_ML, State_Number == state)
  
  # Perform the paired t-test
  max_t_test_ML <- t.test(max_state_data_ML$Max_Disp_ML[max_state_data_ML$Time_Max_ML == "Aft_ML_Max"],
                   max_state_data_ML$Max_Disp_ML[max_state_data_ML$Time_Max_ML == "B4_ML_Max"],
                   paired = TRUE)
  
  # Store the result in the list with the state as the name
  t_test_max_results_ML[[state]] <- max_t_test_ML
  
  # Print the result
  #print(paste("State:", state))
 # print(max_t_test_ML)
  
  # Add the result to the summary table
  t_max_summary_table_ML <- rbind(t_max_summary_table_ML, data.frame(State_Number = state,
                                                   max_P_Value_ML = max_t_test_ML$p.value,
                                                   max_Diff_ML = max_t_test_ML$estimate))
}

##### Max AP
# Loop through each State_Number
for (state in unique(long_df_Max_AP$State_Number)) {
  # Subset the data for the current state
  max_state_data_AP <- subset(long_df_Max_AP, State_Number == state)
  
  # Perform the paired t-test
  max_t_test_AP <- t.test(max_state_data_AP$Max_Disp_AP[max_state_data_AP$Time_Max_AP == "Aft_AP_Max"],
                   max_state_data_AP$Max_Disp_AP[max_state_data_AP$Time_Max_AP == "B4_AP_Max"],
                   paired = TRUE)
  
  # Store the result in the list with the state as the name
  t_test_max_results_AP[[state]] <- max_t_test_AP
  
  # Print the result
 # print(paste("State:", state))
 # print(max_t_test_AP)
  
  # Add the result to the summary table
  t_max_summary_table_AP <- rbind(t_max_summary_table_AP, data.frame(State_Number = state,
                                                   max_P_Value_AP = max_t_test_AP$p.value,
                                                   max_Diff_AP = max_t_test_AP$estimate))
}

#### Access the results for a specific state, e.g., "Anterior-Low"
t_test_mean_results_ML[["Anterior-Low"]]
```

```
## 
##  Paired t-test
## 
## data:  mean_state_data_ML$Mean_Disp_ML[mean_state_data_ML$Time_Mean_ML == "Aft_ML_Abs_Mean"] and mean_state_data_ML$Mean_Disp_ML[mean_state_data_ML$Time_Mean_ML == "B4_ML_Abs_Mean"]
## t = 1.7986, df = 14, p-value = 0.09366
## alternative hypothesis: true mean difference is not equal to 0
## 95 percent confidence interval:
##  -0.2406803  2.7419205
## sample estimates:
## mean difference 
##         1.25062
```

```
# Print the summarized tables
print(t_mean_summary_table_ML)
```

```
##                    State_Number mean_P_Value_ML mean_Diff_ML
## mean difference    Anterior-Low     0.093664155    1.2506201
## mean difference1  Anterior-High     0.001785103    4.3991624
## mean difference2     Medial-Low     0.045451999    0.4193556
## mean difference3    Medial-High     0.024785091    2.1485006
## mean difference4  Posterior-Low     0.003097879    0.8181366
## mean difference5 Posterior-High     0.014669571    1.9673901
## mean difference6    Lateral-Low     0.450461379    0.1311413
## mean difference7   Lateral-High     0.047957308    1.8180929
```

```
print(t_mean_summary_table_AP)
```

```
##                    State_Number mean_P_Value_AP mean_Diff_AP
## mean difference    Anterior-Low    1.161440e-02    0.6063538
## mean difference1  Anterior-High    8.814118e-05    1.1388937
## mean difference2     Medial-Low    4.422894e-04    0.1339865
## mean difference3    Medial-High    1.742753e-01    0.1093475
## mean difference4  Posterior-Low    6.871543e-03    0.4534846
## mean difference5 Posterior-High    5.819394e-05    1.1533227
## mean difference6    Lateral-Low    7.319175e-02    0.1029542
## mean difference7   Lateral-High    1.958646e-03    0.3785303
```

```
print(t_max_summary_table_ML)
```

```
##                    State_Number max_P_Value_ML max_Diff_ML
## mean difference    Anterior-Low   0.0779162331   2.6572409
## mean difference1  Anterior-High   0.0012585567  10.6970876
## mean difference2     Medial-Low   0.0475201528   2.4621094
## mean difference3    Medial-High   0.0737130342   2.3145226
## mean difference4  Posterior-Low   0.0030980287   3.4996557
## mean difference5 Posterior-High   0.0008170172   6.0664788
## mean difference6    Lateral-Low   0.4956967485   0.7701692
## mean difference7   Lateral-High   0.0416270168   3.8293916
```

```
print(t_max_summary_table_AP)
```

```
##                    State_Number max_P_Value_AP max_Diff_AP
## mean difference    Anterior-Low   4.287317e-03   1.9971540
## mean difference1  Anterior-High   3.713341e-05   3.8282138
## mean difference2     Medial-Low   8.875593e-04   0.5785388
## mean difference3    Medial-High   1.443017e-02   0.7452396
## mean difference4  Posterior-Low   1.749533e-05   1.5930047
## mean difference5 Posterior-High   3.122305e-06   4.1963301
## mean difference6    Lateral-Low   2.352457e-02   0.4561417
## mean difference7   Lateral-High   3.690489e-04   1.5898422
```
