# Supplementary material for: Influence of Virtual Reality Illusions on Balance Performance and Immersive User Experience in Young Adults: A Within-Subject Experimental Study
Source: JMIR Serious Games. 2025 Jun 27;13:e70376. doi: 10.2196/70376 (PMC12226963; doi:10.2196/70376)
Supplement: Multimedia Appendix 1 [file games-v13-e70376-s001.zip › Multimedia Appendix/Codes/LME_Abs_Mean.html]

Absolute Mean CoP Displacement after Illusions


# Absolute Mean CoP Displacement after Illusions

#### Achintha Abayasiri

#### 2024-06-30

## Loading the required libraries

This is the best package for reading data into R it is incredibly
fast

```
library(data.table)
```

This is a great package for summarizing data into tables

```
library(dplyr)
```

This is a great package for nice colors

```
library(RColorBrewer)
```

Run libraries for LME, note that you have to download first time
only

```
library(nlme)
require(multcomp)
library(lattice)
```

Other required Packages

```
library(tidyverse)
library(ggsignif)
```

## Setting the Working Directory and Reading the Data

First, we need to make sure R can find the data file

```
setwd("Z:/Data_Collection/Study_1/Participant_Data/Biomechanics_Data")
ex <- fread('CoP_Data_All_fields.csv')
```

This copy paste is only if function below does work

***ex<-read.table(pipe(“pbpaste”),header=T)***

‘fread’ is the function to read data into R using data.table

## Imported Data

Lets check we’ve read the data in properly

```
head(ex)
```

```
##    Participant_Number State_Number   Ill_Dir Ill_Mag B4_AP_Abs_Mean
##                <char>        <int>    <char>  <char>          <num>
## 1:             PS1F03            1  Anterior     Low      0.3284532
## 2:             PS1F03            2  Anterior    High      0.3036768
## 3:             PS1F03            3    Medial     Low      0.2597069
## 4:             PS1F03            4    Medial    High      0.5000495
## 5:             PS1F03            5 Posterior     Low      0.2958926
## 6:             PS1F03            6 Posterior    High      0.2925373
##    B4_ML_Abs_Mean Aft_AP_Abs_Mean Aft_ML_Abs_Mean B4_AP_Max B4_ML_Max
##             <num>           <num>           <num>     <num>     <num>
## 1:      0.2007189       0.6382157       0.4382867 0.8484953 0.6898747
## 2:      0.2667744       1.3080774       0.4977725 1.0183512 0.9638926
## 3:      0.1619572       0.3789147       0.2130429 0.8612730 0.6724250
## 4:      0.2734911       1.1413778       2.4666020 1.7640084 1.0529368
## 5:      0.2519057       1.2998413       0.9411277 0.8229062 0.9477333
## 6:      0.2879892       1.3399095       2.1865327 1.3827584 1.2222034
##    Aft_AP_Max Aft_ML_Max AP_Abs_Mean_All ML_Abs_Mean_All AP_Abs_Std_All
##         <num>      <num>           <num>           <num>          <num>
## 1:   2.427312  1.2572671       0.5497005       0.3704013      0.5497005
## 2:   6.212424  2.4954484       1.0210682       0.4317644      1.0210682
## 3:   1.324807  0.9828079       0.3448508       0.1984451      0.3448508
## 4:   2.929541  4.3602468       0.9581171       1.8399168      0.9581171
## 5:   3.429201  4.1016492       1.0129613       0.7441813      1.0129613
## 6:   4.218612  7.2963612       1.0406211       1.6440206      1.0406211
##    ML_Abs_Std_All
##             <num>
## 1:      0.3704013
## 2:      0.4317644
## 3:      0.1984451
## 4:      1.8399168
## 5:      0.7441813
## 6:      1.6440206
```

Checking internal structure of data frames.

```
str(ex)
```

```
## Classes 'data.table' and 'data.frame':   120 obs. of  16 variables:
##  $ Participant_Number: chr  "PS1F03" "PS1F03" "PS1F03" "PS1F03" ...
##  $ State_Number      : int  1 2 3 4 5 6 7 8 1 2 ...
##  $ Ill_Dir           : chr  "Anterior" "Anterior" "Medial" "Medial" ...
##  $ Ill_Mag           : chr  "Low" "High" "Low" "High" ...
##  $ B4_AP_Abs_Mean    : num  0.328 0.304 0.26 0.5 0.296 ...
##  $ B4_ML_Abs_Mean    : num  0.201 0.267 0.162 0.273 0.252 ...
##  $ Aft_AP_Abs_Mean   : num  0.638 1.308 0.379 1.141 1.3 ...
##  $ Aft_ML_Abs_Mean   : num  0.438 0.498 0.213 2.467 0.941 ...
##  $ B4_AP_Max         : num  0.848 1.018 0.861 1.764 0.823 ...
##  $ B4_ML_Max         : num  0.69 0.964 0.672 1.053 0.948 ...
##  $ Aft_AP_Max        : num  2.43 6.21 1.32 2.93 3.43 ...
##  $ Aft_ML_Max        : num  1.257 2.495 0.983 4.36 4.102 ...
##  $ AP_Abs_Mean_All   : num  0.55 1.021 0.345 0.958 1.013 ...
##  $ ML_Abs_Mean_All   : num  0.37 0.432 0.198 1.84 0.744 ...
##  $ AP_Abs_Std_All    : num  0.55 1.021 0.345 0.958 1.013 ...
##  $ ML_Abs_Std_All    : num  0.37 0.432 0.198 1.84 0.744 ...
##  - attr(*, ".internal.selfref")=<externalptr>
```

## Tag as numbers and factors

```
ex$Participant_Number <- as.factor(ex$Participant_Number)
ex$State_Number <- as.factor(ex$State_Number)
ex$Ill_Dir <- as.factor(ex$Ill_Dir)
ex$Ill_Mag <- as.factor(ex$Ill_Mag)
```

Double check that they are now factors (need to understand if they
are factors or numbers)

```
str(ex)
```

```
## Classes 'data.table' and 'data.frame':   120 obs. of  16 variables:
##  $ Participant_Number: Factor w/ 15 levels "PS1F03","PS1F07",..: 1 1 1 1 1 1 1 1 7 7 ...
##  $ State_Number      : Factor w/ 8 levels "1","2","3","4",..: 1 2 3 4 5 6 7 8 1 2 ...
##  $ Ill_Dir           : Factor w/ 4 levels "Anterior","Lateral",..: 1 1 3 3 4 4 2 2 1 1 ...
##  $ Ill_Mag           : Factor w/ 2 levels "High","Low": 2 1 2 1 2 1 2 1 2 1 ...
##  $ B4_AP_Abs_Mean    : num  0.328 0.304 0.26 0.5 0.296 ...
##  $ B4_ML_Abs_Mean    : num  0.201 0.267 0.162 0.273 0.252 ...
##  $ Aft_AP_Abs_Mean   : num  0.638 1.308 0.379 1.141 1.3 ...
##  $ Aft_ML_Abs_Mean   : num  0.438 0.498 0.213 2.467 0.941 ...
##  $ B4_AP_Max         : num  0.848 1.018 0.861 1.764 0.823 ...
##  $ B4_ML_Max         : num  0.69 0.964 0.672 1.053 0.948 ...
##  $ Aft_AP_Max        : num  2.43 6.21 1.32 2.93 3.43 ...
##  $ Aft_ML_Max        : num  1.257 2.495 0.983 4.36 4.102 ...
##  $ AP_Abs_Mean_All   : num  0.55 1.021 0.345 0.958 1.013 ...
##  $ ML_Abs_Mean_All   : num  0.37 0.432 0.198 1.84 0.744 ...
##  $ AP_Abs_Std_All    : num  0.55 1.021 0.345 0.958 1.013 ...
##  $ ML_Abs_Std_All    : num  0.37 0.432 0.198 1.84 0.744 ...
##  - attr(*, ".internal.selfref")=<externalptr>
```

## LMEs

Runs Linear mixed effects model to determine the effect of magnitude
and direction (onset timing and step) on XX variable of choice.

### ML Direction

Linear Mixed Effects Model for the variable

```
m1_MADis_ML_aft = lme(Aft_ML_Abs_Mean ~ Ill_Mag*Ill_Dir, random = ~1|Participant_Number, data=ex,   method ='ML', na.action = "na.omit")
```

Anova on the built model

```
anova(m1_MADis_ML_aft )
```

```
##                 numDF denDF   F-value p-value
## (Intercept)         1    98 26.263257  <.0001
## Ill_Mag             1    98 18.235121  <.0001
## Ill_Dir             3    98  3.124124  0.0293
## Ill_Mag:Ill_Dir     3    98  0.809346  0.4917
```

Linear Mixed Effects Model for the variable without interaction

```
m2_MADis_ML_aft  = lme(Aft_ML_Abs_Mean ~ Ill_Mag+Ill_Dir, random = ~1|Participant_Number, data=ex,   method ='ML', na.action = "na.omit")
```

Anova for the new model with only main effects

```
anova(m2_MADis_ML_aft)
```

```
##             numDF denDF   F-value p-value
## (Intercept)     1   101 26.966737  <.0001
## Ill_Mag         1   101 18.270885  <.0001
## Ill_Dir         3   101  3.130252   0.029
```

Plotting the effect of each factor separately

```
par(mfrow=c(1,2))

boxplot(Aft_ML_Abs_Mean~Ill_Mag, data=ex, col=brewer.pal(5, "Reds") ,ylab="Absolute Mean Displacement after Illusions in ML Direction (cm)",xlab="Illusion Magnitude")

boxplot(Aft_ML_Abs_Mean~Ill_Dir, data=ex, col=brewer.pal(5, "Blues"),ylab="Absolute Mean Displacement after Illusions in ML Direction (cm)",xlab="Illusion Direction")
```

plotting them all together

```
par(mfrow=c(1,1))
boxplot(Aft_ML_Abs_Mean~Ill_Mag+Ill_Dir, data=ex, col=sample(colors(), 8),ylab="Absolute Mean Displacemeng after Illusions in ML Direction (cm)",xlab="Illusion Type")
```

Fitting the Direction effect to a new model

```
m3_MADis_ML_aft  = lme(Aft_ML_Abs_Mean~ Ill_Dir, random = ~1|Participant_Number, data=ex,   method ='ML', na.action = "na.omit")
```

Anova on model for direction only

```
anova(m3_MADis_ML_aft)
```

```
##             numDF denDF   F-value p-value
## (Intercept)     1   102 27.201230  <.0001
## Ill_Dir         3   102  2.672259  0.0514
```

Tukey post hoc

```
summary(glht(m2_MADis_ML_aft, linfct=mcp(Ill_Dir = "Tukey")), test = adjusted(type = "bonferroni"))
```

```
## 
##   Simultaneous Tests for General Linear Hypotheses
## 
## Multiple Comparisons of Means: Tukey Contrasts
## 
## 
## Fit: lme.formula(fixed = Aft_ML_Abs_Mean ~ Ill_Mag + Ill_Dir, data = ex, 
##     random = ~1 | Participant_Number, method = "ML", na.action = "na.omit")
## 
## Linear Hypotheses:
##                           Estimate Std. Error z value Pr(>|z|)  
## Lateral - Anterior == 0    -1.8316     0.6062  -3.021   0.0151 *
## Medial - Anterior == 0     -1.3173     0.6062  -2.173   0.1786  
## Posterior - Anterior == 0  -1.1938     0.6062  -1.969   0.2935  
## Medial - Lateral == 0       0.5143     0.6062   0.848   1.0000  
## Posterior - Lateral == 0    0.6378     0.6062   1.052   1.0000  
## Posterior - Medial == 0     0.1235     0.6062   0.204   1.0000  
## ---
## Signif. codes:  0 '***' 0.001 '**' 0.01 '*' 0.05 '.' 0.1 ' ' 1
## (Adjusted p values reported -- bonferroni method)
```

### AP Direction

Linear Mixed Effects Model for the variable

```
m1_MADis_AP_aft  = lme(Aft_AP_Abs_Mean ~ Ill_Mag*Ill_Dir, random = ~1|Participant_Number, data=ex,   method ='ML', na.action = "na.omit")
```

Anova on the built model

```
anova(m1_MADis_AP_aft)
```

```
##                 numDF denDF   F-value p-value
## (Intercept)         1    98 111.28304  <.0001
## Ill_Mag             1    98  19.30428  <.0001
## Ill_Dir             3    98  14.03574  <.0001
## Ill_Mag:Ill_Dir     3    98   1.78566  0.1549
```

Linear Mixed Effects Model for the variable without interaction

```
m2_MADis_AP_aft  = lme(Aft_AP_Abs_Mean ~ Ill_Mag+Ill_Dir, random = ~1|Participant_Number, data=ex,   method ='ML', na.action = "na.omit")
```

Anova for the new model with only main effects

```
anova(m2_MADis_AP_aft)
```

```
##             numDF denDF   F-value p-value
## (Intercept)     1   101 114.26383  <.0001
## Ill_Mag         1   101  18.79402  <.0001
## Ill_Dir         3   101  13.66475  <.0001
```

Plotting the effect of each factor separately

```
par(mfrow=c(1,2))

boxplot(Aft_AP_Abs_Mean~Ill_Mag, data=ex, col=brewer.pal(5, "Reds") ,ylab="Absolute Mean Displacement after Illusions in AP Direction (cm)",xlab="Illusion Magnitude")

boxplot(Aft_AP_Abs_Mean~Ill_Dir, data=ex, col=brewer.pal(5, "Blues"),ylab="Absolute Mean Displacement after Illusions in AP Direction (cm)",xlab="Illusion Direction")
```

plotting them all together

```
par(mfrow=c(1,1))
boxplot(Aft_AP_Abs_Mean~Ill_Mag+Ill_Dir, data=ex, col=sample(colors(), 8),ylab="Absolute Mean Displacemeng after Illusions in AP Direction (cm)",xlab="Illusion Type")
```

Fitting the Direction effect to a new model

```
m3_MADis_AP_aft  = lme(Aft_AP_Abs_Mean~ Ill_Dir, random = ~1|Participant_Number, data=ex,   method ='ML', na.action = "na.omit")
```

Anova on model for direction only

```
anova(m3_MADis_AP_aft)
```

```
##             numDF denDF   F-value p-value
## (Intercept)     1   102 115.25742  <.0001
## Ill_Dir         3   102  11.61433  <.0001
```

Tukey post hoc

```
summary(glht(m2_MADis_AP_aft, linfct=mcp(Ill_Dir = "Tukey")), test = adjusted(type = "bonferroni"))
```

```
## 
##   Simultaneous Tests for General Linear Hypotheses
## 
## Multiple Comparisons of Means: Tukey Contrasts
## 
## 
## Fit: lme.formula(fixed = Aft_AP_Abs_Mean ~ Ill_Mag + Ill_Dir, data = ex, 
##     random = ~1 | Participant_Number, method = "ML", na.action = "na.omit")
## 
## Linear Hypotheses:
##                           Estimate Std. Error z value Pr(>|z|)    
## Lateral - Anterior == 0   -0.60134    0.13636  -4.410 6.20e-05 ***
## Medial - Anterior == 0    -0.67662    0.13636  -4.962 4.19e-06 ***
## Posterior - Anterior == 0 -0.02156    0.13636  -0.158 1.000000    
## Medial - Lateral == 0     -0.07528    0.13636  -0.552 1.000000    
## Posterior - Lateral == 0   0.57978    0.13636   4.252 0.000127 ***
## Posterior - Medial == 0    0.65506    0.13636   4.804 9.34e-06 ***
## ---
## Signif. codes:  0 '***' 0.001 '**' 0.01 '*' 0.05 '.' 0.1 ' ' 1
## (Adjusted p values reported -- bonferroni method)
```

```
# t test

#ML
t.test(ex$Aft_ML_Abs_Mean[ex$Ill_Mag == "High"],
       ex$Aft_ML_Abs_Mean[ex$Ill_Mag == "Low"],
       paired = TRUE)
```

```
## 
##  Paired t-test
## 
## data:  ex$Aft_ML_Abs_Mean[ex$Ill_Mag == "High"] and ex$Aft_ML_Abs_Mean[ex$Ill_Mag == "Low"]
## t = 3.9269, df = 59, p-value = 0.0002278
## alternative hypothesis: true mean difference is not equal to 0
## 95 percent confidence interval:
##  0.9179114 2.8252696
## sample estimates:
## mean difference 
##        1.871591
```

```
#AP
t.test(ex$Aft_AP_Abs_Mean[ex$Ill_Mag == "High"],
       ex$Aft_AP_Abs_Mean[ex$Ill_Mag == "Low"],
       paired = TRUE)
```

```
## 
##  Paired t-test
## 
## data:  ex$Aft_AP_Abs_Mean[ex$Ill_Mag == "High"] and ex$Aft_AP_Abs_Mean[ex$Ill_Mag == "Low"]
## t = 4.4749, df = 59, p-value = 3.548e-05
## alternative hypothesis: true mean difference is not equal to 0
## 95 percent confidence interval:
##  0.2360618 0.6179370
## sample estimates:
## mean difference 
##       0.4269994
```
