# Supplementary material for: Influence of Virtual Reality Illusions on Balance Performance and Immersive User Experience in Young Adults: A Within-Subject Experimental Study
Source: JMIR Serious Games. 2025 Jun 27;13:e70376. doi: 10.2196/70376 (PMC12226963; doi:10.2196/70376)
Supplement: Multimedia Appendix 1 [file games-v13-e70376-s001.zip › Multimedia Appendix/Codes/Directional_Tendency.html]

Directional Tendancy


# Directional Tendancy

#### Achintha Abayasiri

#### 2024-12-20

```
# Load necessary libraries
library(ggplot2)
library(gridExtra)
library(data.table)

# Set the working directory
setwd("Z:/Data_Collection/Study_1/Participant_Data/Biomechanics_Data/Directionality")

# Initialize an empty list to store data frames
data_list <- list()

for (i in 0:8) {
  dummy_name <- paste0("CoP_Data_All_Directional_Means_test", i)
  file_name <- paste0(dummy_name, ".csv")
  data <- fread(file_name)
  # Check if necessary columns exist
  if (!all(c("Mean_Aft_ML", "Mean_Aft_AP", "Illusion", "Magnitude") %in% names(data))) {
    stop(paste("Missing necessary columns in", file_name))
  }
  data_list[[i+1]] <- data
}

# Titles for each plot
titles <- c(
  "No illusion state",
  "Anterior-Low Illusion",
  "Anterior-High Illusion",
  "Medial-Low Illusion",
  "Medial-High Illusion",
  "Posterior-Low Illusion",
  "Posterior-High Illusion",
  "Lateral-Low Illusion",
  "Lateral-High Illusion"
)

# Create a list to store plots and a data frame to store summary table
plot_list <- list()
plot_list1 <- list()
summary_table <- data.frame(i = 1:9, Q1 = numeric(9), Q2 = numeric(9), Q3 = numeric(9), Q4 = numeric(9))
# Define custom shape mapping for Magnitude
shape_mapping <- c(
  "None" = 19, # Solid circle
  "Low" = 17, # Triangle
  "High" = 15 # Square
)

# Define custom color mapping for Illusion
color_mapping <- c(
  "Neutral" = "black",
  "Anterior" = "blue",
  "Medial" = "red",
  "Posterior" = "green",
  "Lateral" = "darkorange"
)

for (i in 1:9) {  
  New_title <- paste0("Mean CoP Displacement ", titles[i])
  
  # Create a new column for the combined Illusion and Magnitude
  data_list[[i]]$Illusion_Magnitude <- paste(data_list[[i]]$Illusion, data_list[[i]]$Magnitude, sep = "_")
  
  # Calculate percentages for each quadrant
  data_list[[i]]$Quadrant <- as.factor(data_list[[i]]$Quadrant)
  quadrant_counts <- table(data_list[[i]]$Quadrant)
  quadrant_percentages <- round(prop.table(quadrant_counts) * 100, 1)
  
  # Store the quadrant percentages in the summary table
  summary_table[i, "Q1"] <- quadrant_percentages["Q1"]
  summary_table[i, "Q2"] <- quadrant_percentages["Q2"]
  summary_table[i, "Q3"] <- quadrant_percentages["Q3"]
  summary_table[i, "Q4"] <- quadrant_percentages["Q4"]
  
  p <- ggplot(data_list[[i]], aes(x = Mean_Aft_ML, y = Mean_Aft_AP, color = Illusion, shape = Magnitude)) +
    geom_point(size = 3) +
    geom_vline(xintercept = 0, color = "black", linetype = "dashed") +
    geom_hline(yintercept = 0, color = "black", linetype = "dashed") +
    scale_x_continuous(expand = c(0,0), limits = c(min(data_list[[i]]$Mean_Aft_ML)-1, max(data_list[[i]]$Mean_Aft_ML)+1)) +
    scale_y_continuous(expand = c(0,0), limits = c(min(data_list[[i]]$Mean_Aft_AP)-1, max(data_list[[i]]$Mean_Aft_AP)+1)) +
    scale_shape_manual(values = shape_mapping) +
    scale_color_manual(values = color_mapping) +
    labs(title = New_title,
         y = "CoP Displacement in AP Direction (cm)",
         x = "CoP Displacement in ML Direction (cm)") +
    theme(panel.background = element_rect(fill = "white", color = NA),
          panel.border = element_rect(color = "black", fill = NA, size = 1),
          axis.title = element_text(size = 10),
          axis.text = element_text(size = 10),
          plot.title = element_text(size = 10, hjust = 0.5),
          legend.position = "none")  # Remove legend
  
  plot_list[[i]] <- p
}
```

```
## Warning: The `size` argument of `element_rect()` is deprecated as of ggplot2 3.4.0.
## ℹ Please use the `linewidth` argument instead.
## This warning is displayed once every 8 hours.
## Call `lifecycle::last_lifecycle_warnings()` to see where this warning was
## generated.
```

```
# Arrange individual plots in a grid layout (with 3 columns to fit 9 plots)
grid.arrange(grobs = plot_list, ncol = 3)
```

```
# Combine all data into a single data frame
combined_data <- rbindlist(data_list)

# Create the plot for all combined data
combined_plot <- ggplot(combined_data, aes(x = Mean_Aft_ML, y = Mean_Aft_AP, color = Illusion, shape = Magnitude)) +
  geom_point(size = 3) +
  geom_vline(xintercept = 0, color = "black", linetype = "dashed") +
  geom_hline(yintercept = 0, color = "black", linetype = "dashed") +
  scale_x_continuous(expand = c(0,0), limits = c(min(combined_data$Mean_Aft_ML)-1, max(combined_data$Mean_Aft_ML)+1)) +
  scale_y_continuous(expand = c(0,0), limits = c(min(combined_data$Mean_Aft_AP)-1, max(combined_data$Mean_Aft_AP)+1)) +
  scale_shape_manual(values = shape_mapping) +
  scale_color_manual(values = color_mapping) +
  labs(
       y = "Mean CoP Displacement in AP (cm)",
       x = "Mean CoP Displacement in ML (cm)") +
  theme(panel.background = element_rect(fill = "white", color = NA),
        panel.border = element_rect(color = "black", fill = NA, size = 1),
        axis.title = element_text(size = 8),
        axis.text = element_text(size = 8),
        plot.title = element_text(size = 8, hjust = 0.5),
        legend.position = "bottom", # Place the legend at the bottom
        legend.title = element_blank(), # Remove legend title
        legend.text = element_text(size = 14),
        legend.box.spacing = unit(0.5, "cm")) # Adjust spacing around the legend box

# Print the combined plot separately
print(combined_plot)
```

```
# Print the summary table
print(summary_table)
```

```
##   i   Q1   Q2   Q3   Q4
## 1 1 53.3 26.7   NA 20.0
## 2 2 26.7  6.7 40.0 26.7
## 3 3 20.0  6.7 53.3 20.0
## 4 4 33.3 26.7  6.7 33.3
## 5 5 40.0   NA  6.7 53.3
## 6 6 40.0 20.0 33.3  6.7
## 7 7 46.7 13.3 20.0 20.0
## 8 8 33.3 46.7 13.3  6.7
## 9 9 13.3 33.3 46.7  6.7
```
