# Supplementary material for: Influence of Virtual Reality Illusions on Balance Performance and Immersive User Experience in Young Adults: A Within-Subject Experimental Study
Source: JMIR Serious Games. 2025 Jun 27;13:e70376. doi: 10.2196/70376 (PMC12226963; doi:10.2196/70376)
Supplement: Multimedia Appendix 1 [file games-v13-e70376-s001.zip › Multimedia Appendix/Codes/Performance_n_CoP_Stat.html]

Performance vs CoP


# Performance vs CoP

#### Achintha Abayasiri

#### 2025-04-06

### This code runs Chi-Square tests, LMEs for performance data and CoP data

Loading the required libraries

Setting the Working Directory and Reading the Data

```
setwd("Z:/Data_Collection/Study_1/Participant_Data/Performance_Data")
ex <- fread('ALL_CoP_n_Perform_Data_New.csv')
```

Tag as numbers and factors

```
ex$Participant_Number <- as.factor(ex$Participant_Number)
ex$State_Number <- as.factor(ex$State_Number)
ex$Ill_Dir <- as.factor(ex$Ill_Dir)
ex$Ill_Mag <- as.factor(ex$Ill_Mag)
ex$Hit_Success <- as.factor(ex$Hit_Success)
ex$Ill_Mag <- factor(ex$Ill_Mag, levels = c("Low", "High"))
ex$Ill_Dir <- factor(ex$Ill_Dir, levels = c("Anterior", "Posterior", "Medial","Lateral"))
```

## Comparison of CoP in Success vs Unsucess

```
# We'll compute mean CoP_AP and CoP_ML per Participant and Hit_Success
participant_means <- ex %>%
  group_by(Participant_Number, Hit_Success) %>%
  summarise(
    Mean_CoP_AP = mean(CoP_AP, na.rm = TRUE),
    Mean_CoP_ML = mean(CoP_ML, na.rm = TRUE),
    .groups = "drop"
  )

# Spread the data to wide format for paired comparison
cop_wide <- participant_means %>%
  tidyr::pivot_wider(
    names_from = Hit_Success,
    values_from = c(Mean_CoP_AP, Mean_CoP_ML)
  )

# Optional: remove participants who don't have both Hit and Miss
cop_wide <- cop_wide %>%
  filter(!is.na(Mean_CoP_AP_Hit), !is.na(Mean_CoP_AP_Miss),
         !is.na(Mean_CoP_ML_Hit), !is.na(Mean_CoP_ML_Miss))

# Paired t-test for CoP_AP
t_test_ap <- t.test(cop_wide$Mean_CoP_AP_Hit, cop_wide$Mean_CoP_AP_Miss, paired = TRUE)
print(t_test_ap)
```

```
## 
##  Paired t-test
## 
## data:  cop_wide$Mean_CoP_AP_Hit and cop_wide$Mean_CoP_AP_Miss
## t = -6.5158, df = 12, p-value = 2.869e-05
## alternative hypothesis: true mean difference is not equal to 0
## 95 percent confidence interval:
##  -0.6862896 -0.3423318
## sample estimates:
## mean difference 
##      -0.5143107
```

```
# Paired t-test for CoP_ML
t_test_ml <- t.test(cop_wide$Mean_CoP_ML_Hit, cop_wide$Mean_CoP_ML_Miss, paired = TRUE)
print(t_test_ml)
```

```
## 
##  Paired t-test
## 
## data:  cop_wide$Mean_CoP_ML_Hit and cop_wide$Mean_CoP_ML_Miss
## t = -2.4372, df = 12, p-value = 0.03132
## alternative hypothesis: true mean difference is not equal to 0
## 95 percent confidence interval:
##  -2.3619128 -0.1321934
## sample estimates:
## mean difference 
##       -1.247053
```

```
par(mfrow=c(1,2))

boxplot(cop_wide$Mean_CoP_AP_Hit, cop_wide$Mean_CoP_AP_Miss, col=brewer.pal(5, "Blues") ,ylab="Absolute CoP Displacement in AP Direction (cm)",xlab="Gameplay Status")

boxplot(cop_wide$Mean_CoP_ML_Hit, cop_wide$Mean_CoP_ML_Miss, col=brewer.pal(5, "Reds") ,ylab="Absolute CoP Displacement in ML Direction (cm)",xlab="Gameplay Status")
```

```
#Calculate mean and SD of participant-level means
# CoP_AP
mean_hit_ap  <- mean(cop_wide$Mean_CoP_AP_Hit, na.rm = TRUE)
sd_hit_ap    <- sd(cop_wide$Mean_CoP_AP_Hit, na.rm = TRUE)

mean_miss_ap <- mean(cop_wide$Mean_CoP_AP_Miss, na.rm = TRUE)
sd_miss_ap   <- sd(cop_wide$Mean_CoP_AP_Miss, na.rm = TRUE)

# CoP_ML
mean_hit_ml  <- mean(cop_wide$Mean_CoP_ML_Hit, na.rm = TRUE)
sd_hit_ml    <- sd(cop_wide$Mean_CoP_ML_Hit, na.rm = TRUE)

mean_miss_ml <- mean(cop_wide$Mean_CoP_ML_Miss, na.rm = TRUE)
sd_miss_ml   <- sd(cop_wide$Mean_CoP_ML_Miss, na.rm = TRUE)

# Step 4: Print results
cat("Participant-level CoP_AP (Hit):   Mean =", round(mean_hit_ap, 3), ", SD =", round(sd_hit_ap, 3), "\n")
```

```
## Participant-level CoP_AP (Hit):   Mean = 0.961 , SD = 0.405
```

```
cat("Participant-level CoP_AP (Miss):  Mean =", round(mean_miss_ap, 3), ", SD =", round(sd_miss_ap, 3), "\n")
```

```
## Participant-level CoP_AP (Miss):  Mean = 1.475 , SD = 0.49
```

```
cat("Participant-level CoP_ML (Hit):   Mean =", round(mean_hit_ml, 3), ", SD =", round(sd_hit_ml, 3), "\n")
```

```
## Participant-level CoP_ML (Hit):   Mean = 2.257 , SD = 1.826
```

```
cat("Participant-level CoP_ML (Miss):  Mean =", round(mean_miss_ml, 3), ", SD =", round(sd_miss_ml, 3), "\n")
```

```
## Participant-level CoP_ML (Miss):  Mean = 3.504 , SD = 2.611
```

Plotting the comparisons of each direction separately

```
par(mfrow=c(1,2))

boxplot(CoP_AP~Hit_Success, data=ex, col=brewer.pal(5, "Blues") ,ylab="Absolute CoP Displacement in AP Direction (cm)",xlab="Gameplay Status")

boxplot(CoP_ML~Hit_Success, data=ex, col=brewer.pal(5, "Reds") ,ylab="Absolute CoP Displacement in ML Direction (cm)",xlab="Gameplay Status")
```

## CHI Squared test

```
# Create a contingency table
contingency_table <- table(ex$Ill_Dir, ex$Hit_Success)

# Print Contingency Table
print(contingency_table)
```

```
##            
##             Hit Miss
##   Anterior  249  401
##   Posterior 360  290
##   Medial    561   89
##   Lateral   545  105
```

```
# Calculate percentages within each Ill_Dir (row-wise)
percent_table <- prop.table(contingency_table, margin = 1) * 100

# Round for better readability
percent_table <- round(percent_table, 1)

# Print the percentage table
print(percent_table)
```

```
##            
##              Hit Miss
##   Anterior  38.3 61.7
##   Posterior 55.4 44.6
##   Medial    86.3 13.7
##   Lateral   83.8 16.2
```

```
# Perform chi-squared test
chi_squared_test <- chisq.test(contingency_table)

# Print the test results
print(chi_squared_test)
```

```
## 
##  Pearson's Chi-squared test
## 
## data:  contingency_table
## X-squared = 466.22, df = 3, p-value < 2.2e-16
```

## LMEs

Linear Mixed Effects Model for the score

```
m1_Score = lme(Score ~ Ill_Mag*Ill_Dir, random = ~1|Participant_Number, data=ex,   method ='ML', na.action = "na.omit")
m2_Score = lme(Score ~ Ill_Dir, random = ~1|Participant_Number, data=ex,   method ='ML', na.action = "na.omit")
```

Anova on the built model

```
anova(m1_Score)
```

```
##                 numDF denDF  F-value p-value
## (Intercept)         1    84 507.0057  <.0001
## Ill_Mag             1    84  28.6443  <.0001
## Ill_Dir             3    84  31.9931  <.0001
## Ill_Mag:Ill_Dir     3    84   8.0728   1e-04
```

Plotting the effect of each factor separately

```
par(mfrow=c(1,3))

boxplot(Score~Ill_Mag, data=ex, col=brewer.pal(5, "Greens") ,ylab="Number of Successful Returns out of 25 Turns",xlab="Illusion Magnitude")

boxplot(Score~Ill_Dir, data=ex, col=brewer.pal(5, "Blues"),ylab="Number of Successful Returns out of 25 Turns",xlab="Illusion Direction")

boxplot(Score~Ill_Mag+Ill_Dir, data=ex, col=brewer.pal(9, "Reds"),ylab="Number of Successful Returns out of 25 Turns",xlab="Illusion Type")
```

plotting them all together

```
par(mfrow=c(1,1))
boxplot(Score~Ill_Mag+Ill_Dir, data=ex, col=brewer.pal(9, "Reds"),ylab="Number of Successful Returns out of 25 Turns",xlab="Illusion Type")
```

Tukey post hoc to tell you which directions are significantly
different (Since only two factors for magnitude, Tukey post hoc is not
performed for Magnitude)

```
summary(glht(m2_Score, linfct=mcp(Ill_Dir = "Tukey")), test = adjusted(type = "bonferroni"))
```

```
## 
##   Simultaneous Tests for General Linear Hypotheses
## 
## Multiple Comparisons of Means: Tukey Contrasts
## 
## 
## Fit: lme.formula(fixed = Score ~ Ill_Dir, data = ex, random = ~1 | 
##     Participant_Number, method = "ML", na.action = "na.omit")
## 
## Linear Hypotheses:
##                           Estimate Std. Error z value Pr(>|z|)    
## Posterior - Anterior == 0   4.2692     1.7761   2.404  0.09737 .  
## Medial - Anterior == 0     12.0000     1.7761   6.757 8.48e-11 ***
## Lateral - Anterior == 0    11.3846     1.7761   6.410 8.73e-10 ***
## Medial - Posterior == 0     7.7308     1.7761   4.353 8.07e-05 ***
## Lateral - Posterior == 0    7.1154     1.7761   4.006  0.00037 ***
## Lateral - Medial == 0      -0.6154     1.7761  -0.346  1.00000    
## ---
## Signif. codes:  0 '***' 0.001 '**' 0.01 '*' 0.05 '.' 0.1 ' ' 1
## (Adjusted p values reported -- bonferroni method)
```
